# ***imply***: improving cell-type deconvolution accuracy using personalized reference profiles

**DOI:** 10.1186/s13073-024-01338-z

**Published:** 2024-04-29

**Authors:** Guanqun Meng, Yue Pan, Wen Tang, Lijun Zhang, Ying Cui, Fredrick R. Schumacher, Ming Wang, Rui Wang, Sijia He, Jeffrey Krischer, Qian Li, Hao Feng

**Affiliations:** 1 Department of Population and Quantitative Health Sciences, Case Western Reserve University, Cleveland, 44106 OH USA; 2 Department of Biostatistics, St. Jude Children’s Research Hospital, Memphis, 38105 TN USA; 3 Department of Biomedical Data Science, Stanford University, Stanford, 94305 CA USA; 4 Department of Surgery, Division of Surgical Oncology, University Hospitals Cleveland Medical Center, Cleveland, 44106 OH USA; 5 Department of Biostatistics, University of Michigan, Ann Arbor, 48109 MI USA; 6 Health Informatics Institute, University of South Florida, Tampa, 38105 FL USA

**Keywords:** Deconvolution, Bulk RNA-seq, Personalized reference, Admixed samples, Cell-type-specific

## Abstract

Using computational tools, bulk transcriptomics can be deconvoluted to estimate the abundance of constituent cell types. However, existing deconvolution methods are conditioned on the assumption that the whole study population is served by a single reference panel, ignoring person-to-person heterogeneity. Here, we present ***imply***, a novel algorithm to deconvolute cell type proportions using personalized reference panels. Simulation studies demonstrate reduced bias compared with existing methods. Real data analyses on longitudinal consortia show disparities in cell type proportions are associated with several disease phenotypes in Type 1 diabetes and Parkinson’s disease. ***imply*** is available through the R/Bioconductor package *ISLET* at https://bioconductor.org/packages/ISLET/.

**Supplementary Information:**

The online version contains supplementary material available at 10.1186/s13073-024-01338-z.

## Background

Tissues are complex samples composed of different cell types, and real bulk transcriptomic data are often weighted sums of multiple signals over several different cell types [19]. In large-scale and population-level clinical studies, like Parkinson’s Disease Biomarkers Program (PDBP) and The Cancer Genome Atlas (TCGA), transcriptomic samples are often collected from complex tissues. For admixed tissue samples, differentially expressed transcriptional profiles from different phenotypical groups can be caused by either cell-type composition disparities or underlying cell-type-specific (CTS) gene expression heterogeneity. Studies have shown that cell type proportions are confounders with other phenotypical covariates like age, sex, or clinical outcomes, for bulk transcriptomic data analysis [5, 6]. As a result, ignoring CTS compositions in gene expression analysis would cause inflated false positive rates of identifying relevant genetic features. An accurate cell type proportion deconvolution is thus vital, especially for cell types with low abundance and weak biological signals, where the real biological differences could be shadowed by technical noises [5, 30, 39].

Recently, several statistical and deep learning methods have been proposed to deconvolute cell type abundance from bulk transcriptome data. These methods utilize linear least squares regression [12, 49, 55], quadratic programming [25], support vector regression [11, 43], non-negative matrix factorization [21, 45], and deep neural networks (DNNs) [9, 38]. These methods share the same goal of quantifying the unknown abundances of various cell types and can be broadly summarized into two categories: Reference-Based (RB) and Reference-Free (RF). The RB deconvolution relies on a cell-type-specific (CTS) gene expression signature matrix (reference panel) composed of the pre-selected features known to differentiate cell types, while the RF deconvolution estimates cell type proportions in the absence of a reference panel. Naturally, the accuracy of cell type abundance inference is dependent on the quality of signature matrices, and a more accurate reference panel is beneficial for improving cell type abundance estimations [6]. RF deconvolution, in contrast, offers flexibility where reference panels are hard to obtain.

Currently, all RB deconvolution methods require a reference panel as the input across all subjects. For example, CIBERSORT [43], an RB deconvolution method [5, 6], provides a verified signature panel LM22. It is useful for leukocyte deconvolution and includes 547 marker genes which could distinguish 22 hematopoietic cell types. xCell [4] combines the gene set enrichment with deconvolution techniques and introduces curated gene signatures representing 64 distinct cell types, including a wide range of both adaptive and innate immune cells. However, it is a very strong assumption to use a single reference panel across the whole population. This assumption ignores person-to-person heterogeneity for CTS gene expression, and deviates from the biological fact that the gene expression profile could vary, even for one purified cell type, depending on environmental influences, age, sex, subject’s health status, and treatment paradigms [1, 8, 15, 18, 23, 27, 28, 40, 48]. Mismatched reference signatures can impact the deconvolution accuracy [22, 47]. The problem is even exacerbated when handling longitudinally observed and repeatedly-measured data, when intra-subject samples share information and inter-subject heterogeneities are relatively strong. Recent research shows that models incorporating personalized effects can accurately retrieve cell type reference panels on an individual-basis [16]. However, to date, no method is available to take advantage of personalized reference panels to precisely deconvolute cell type proportions, especially when longitudinal samples are available.

**Fig. 1:**
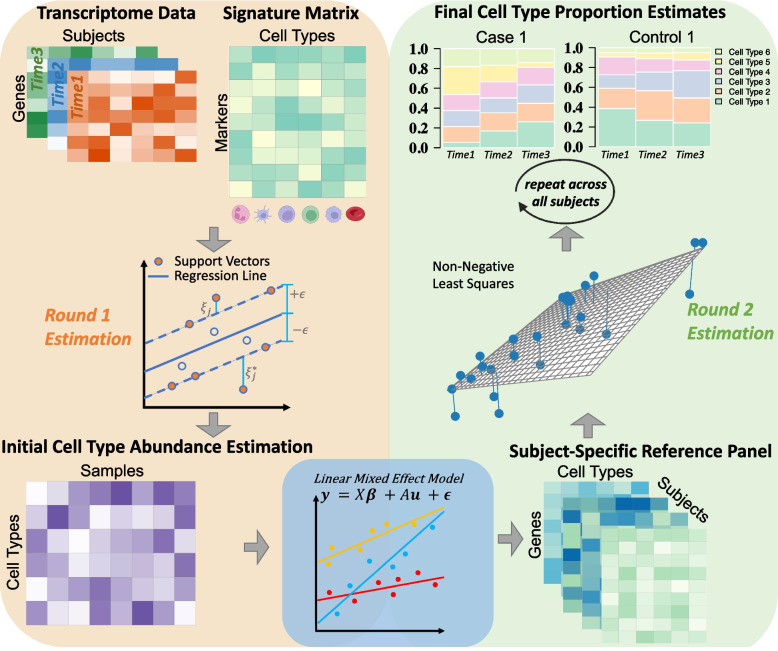
Overview of ***imply***’s personalized deconvolution. The **top-left** shows two inputs: repetitively measured transcriptome data and a signature matrix containing CTS marker genes. In *Stage I*, depicted in the **middle left**, the initial step adopts support vector regression to derive a preliminary cell type abundance, as shown in the **bottom-left**. Next, for *Stage II*, as shown in the **bottom-center**, linear mixed effect models are utilized to reconstruct personalized references, which are shown in the **bottom-right**. In *Stage III*, as illustrated in the **middle-right**, by employing *non-negative least squares* and using personalized references generated from the previous step, repeatedly across all subjects, ***imply*** enables personalized deconvolution to produce cell type proportion estimates, shown on the **top-right**

Here we develop a new deconvolution algorithm ***imply*** (***imp****roving ce* ***l****l-t* ***y****pe deconvolution using personalized reference*) as depicted in Fig. 1. ***imply*** can utilize personalized reference panels to precisely deconvolute cell type proportions using longitudinal or repeatedly measured data. It borrows information across the repeatedly measured transcriptome samples within each subject, to recover personalized reference panels. The personalized references are further adopted to improve cell type deconvolution. The method consists of three stages. In the first stage, using a commonly shared reference panel across the population, we deconvolute the bulk transcriptomic data and estimate initial cell type proportions. The first stage is based on support vector regression (SVR), as it has been shown to be a leading framework for conventional deconvolution problems [43]. In the second stage, we use a mixed-effect modeling framework to retrieve personalized reference panels based on subjects’ phenotypical information, observed bulk transcriptomic data, and the initial cell type proportions from the first stage. In the third and final stage, we use the recovered personalized reference panels, together with repeated measurement of bulk transcriptomic data for each subject, to estimate cell type proportions. The rationale for using this three-stage approach is straightforward: the personalized reference panel is more accurate compared with the population-level signature. Naturally, using this more accurate reference panel can consequently lead to a more precise deconvolution.

We conducted extensive *in silico* simulations and real data analyses to test the performance of ***imply***. The simulation results showed significantly increased accuracies in cell type proportion estimation compared with existing approaches. Our method ***imply*** reduced bias in deconvolution, and increased the correlation between the estimated and the ground-truth cell type abundance. Real data analyses on two large longitudinal consortia, The Environmental Determinants of Diabetes in the Young (TEDDY study) [29] and Parkinson’s Disease Biomarkers Program (PDBP study), showed more realistic deconvolution results that align with low-throughput experiments. The results suggested that disparities in cell type proportions of certain cell types are associated with several disease phenotypes in Type 1 diabetes and Parkinson’s disease. Our method ***imply*** has been implemented and integrated into the Bioconductor package *ISLET* [17] and is available at https://bioconductor.org/packages/ISLET/.

## Methods

### Overview of ***imply***

The primary objective of ***imply*** is to improve the accuracy of cell abundance estimations through the usage of a “subject- and cell-type specific reference panel”, which is a personalized CTS reference panel unique to each study participant. The algorithm is structured into three stages. In *Stage I*, the initial cell type compositions will be estimated using a population-level CTS reference panel. This first stage is very much alike existing deconvolution frameworks, but provides a valid initial estimation for downstream stages. The core component of ***imply*** lies in *Stage II*, where we optimize the usage of repeatedly-measured samples within each subject. Multi-measured samples, within each subject, are assumed to share the same reference panel but have cell type composition variations. Here, mixed-effect modeling is naturally adopted to capture the group-level average (fixed effect) and subject-level deviations (random effect). The output from this stage, for each subject, is a personalized reference panel. In *Stage III*, the personalized deconvolution can be easily conducted by adopting the personalized reference panel from *Stage II*, for each subject.

#### Model notations

We use *g* to index the features (e.g., genes), where $g=1,2,3,\dots , G$, and *n* to index the study subjects, where $n=1,2,3,\dots , N$. For each subject *n*, the repeated or longitudinal samples are indexed by *i*, where $i=1,2,\dots , t_n$. For each subject *n*, the observed bulk transcriptome dataset can be represented by $\varvec{y_n}$ below, which is of dimension $G \times t_n$:

$$\begin{aligned} \varvec{y_n}=\left[ \begin{array}{cccc} y_{1n1} &{} y_{1n2} &{}\dots &{} y_{1nt_n} \\ y_{2n1} &{} y_{2n2} &{}\dots &{} y_{2nt_n} \\ \vdots &{} \vdots &{} y_{gni} &{}\vdots \\ y_{Gn1} &{} y_{Gn2} &{}\dots &{} y_{Gnt_n} \end{array} \right] \end{aligned}$$

Here, each element $y_{gni}$ in $\varvec{y_n}$ has three indexes: *g* to index the feature, *n* to index the subject, and *i* to index the sample. The whole bulk transcriptome dataset from all subjects, which can be represented as $\varvec{Y} = (\varvec{y_1}, \varvec{y_2}, \cdots , \varvec{y_N})$, is of dimension $G \times T$. Here, *T* is the total number of samples across *N* subjects, and thus $T= \sum _{n=1}^{N}t_n$. We use *k* to index cell types, where $k=1,2,\dots , K$.

#### Stage I: Initial cell type proportion estimation

Initially, similar to existing deconvolution methods, it is easy to obtain a single reference panel $\varvec{E}$ of dimension $J \times K(J < G)$ for the study population, where *J* indicates the total number of usable and discriminative signature genes for deconvolution. Previous studies have demonstrated the feasibility of constructing a reference panel from pure cell line data or annotated single-cell RNA-seq (scRNA-seq) data [44, 49], and thus $\varvec{E}$ can be treated as known. With the observed bulk data $\varvec{y_n}$ and the initial reference panel $\varvec{E}$, as illustrated in the top-left of Fig. 1, the first-round reference-based ‘coarse’ deconvolution is conducted using a $\nu$-Support Vector Regression algorithm ($\nu$-SVR) based on a linearity assumption. Such strategy was already proven to be a successful choice in leading deconvolution algorithms. To be specific, this stage requires both the signature matrix $\varvec{E}$ and the feature-overlapped RNA-sequencing data $\varvec{y_n}$, comprising only the overlapping features filtered by marker genes from the signature matrix. For each subject *n* and sample *i*, the deconvolution is thus a regression problem: $\varvec{y}_{\cdot ni} = \varvec{E} \varvec{\theta }_{E,ni\cdot } + \varvec{b}$, where $\varvec{b}\in R^J$ is the error term. Our initial deconvolution parameter-of-interest is $\varvec{\theta }_{E,ni\cdot }$, and can be estimated by minimizing the following objective function:

$$\begin{aligned} \frac{1}{2} \Vert \varvec{\theta }_{E,ni\cdot } \Vert ^2 + C\sum \limits _{j=1}^{J}{ \left(\xi _j+\xi _j^* \right)}, \ \ \xi _j, \xi _j^* >0 \end{aligned}$$

The solved $\hat{\varvec{\theta }}_{E,ni\cdot }=\left(\hat{\theta }_{E,ni1}, \hat{\theta }_{E,ni2},\dots \hat{\theta }_{E,niK}\right)'$, for each sample, is a cell type abundance vector of dimension $K \times 1$. The constraints of the objective function and parameters of $\epsilon$, *C*, $\xi _j$, and $\xi _j^*$ are detailed in the Additional file 1: Method Details. Negative estimates in $\hat{\varvec{\theta }}_{E,ni\cdot }$ are set to 0, and the remaining coefficients are normalized to sum-to-one, which is the general practice in proportion deconvolution. Repeating this process for all *T* samples across all subjects, we obtain the deconvoluted cell compositions. It is worth noting that although this deconvolution stage has little difference from existing methods, it provides a valid initial estimation for downstream steps.

#### Stage II: Personalized reference panel recovery

In this stage, the inputs are the original bulk transcriptome data $\varvec{y_n}$, and the solved cell type compositions $\hat{\varvec{\theta }}_{E,ni\cdot }$, for all samples from subject *n*. The goal is to solve for “subject- and cell-type-specific” reference panels. The key is to optimize the usage of repeatedly-measured samples within each subject. In this stage, we make an assumption that the multi-measured samples, within each subject, would share the same CTS reference panel. In other words, the transcriptome variations in observed bulk samples, within each subject, are primarily caused by cell type composition discrepancies. This is a moderate assumption, considering the compositional nature of multiple samples from the same tissue (for example, samples from multiple regions per brain). Here, mixed-effect regression would be a natural choice to capture the group-wise transcriptome average (fixed effect) and subject-level deviations (random effect) from the group average. Such modeling also allows for the consideration of additional covariates (Additional file 1: Method Details). Using the original bulk transcriptome data $\varvec{y_n}$ and the cell-type-specific and sample-specific compositions $\hat{\theta }_{E,nik}$ from *Stage I*, the following linear mixed-effect regression can be formulated for each gene *g*. Here, we drop the gene index *g* to simplify notation, but note this framework can be applied in parallel to all genes-of-interest to solve for “subject- and cell-type-specific” references.

$$\begin{aligned} E(y_{ni}) = \sum \limits _{k=1}^K \left( m_{k}+ \beta _kz_n +u_{nk}\right) \hat{\theta }_{E,nik} \end{aligned}$$

Here, the known independent variables are a group label $z_n$ for each subject *n*, and estimated cell type compositions $\hat{\theta }_{E,nik}$ from *Stage I* for all samples. The coefficients-of-interest include group-level fixed effects $m_{k}$, $\beta _k$, and subject-level random effect $u_{nk}$. The interpretation is straightforward: $m_{k}$ is the average gene expression level in cell type *k* for the control group ($z_n=0$), and $m_{k}+\beta _k$ is the average gene expression in cell type *k* for the case group ($z_n=1$). Apparently, $\beta _k$ is the differential expression across the two groups for a cell type *k*. Most importantly, the random effect $u_{nk}$ represents a subject-specific deviation from the group-wise mean expression in cell type *k*. Note this modeling example above reflects the most basic scenario where study subjects originate from two groups (for example, cancer versus normal), where a binary scalar $z_n$ is adopted to indicate group labels. This modeling can be extended naturally to incorporate additional covariates, either at the cell-type level or the subject-level. Modeling details and design matrices setup specified in the Additional file 1: Method Details. $\hat{m}_k$, $\hat{\beta }_k$ and $\hat{u}_{nk}$ are obtained by penalized least square algorithm with restricted maximum likelihood. The subject- and cell-type-specific reference panel is obtained by addition (fixed effect + random effect), with respect to each corresponding condition, cell type, and subject. To be specific, for subject *n* and cell type *k*, its purified reference expression is $r_{nk} = \hat{m}_{k}+z_n\hat{\beta }_{k} + \hat{u}_{nk}$. After repeating the same model for all *G* genes and adding gene index *g* back to $r_{gnk}$, the personalized reference panel for each subject *n* can be represented by a matrix $\varvec{R}_n$ of dimension $G\times K$:

$$\begin{aligned} \varvec{R}_n = \left[ \begin{array}{cccc} r_{1n1} &{}r_{1n2} &{} \dots &{} r_{1nK} \\ r_{2n1} &{}r_{2n2} &{} \dots &{} r_{2nK} \\ \vdots &{} &{} \ddots &{} \\ r_{Gn1} &{}r_{Gn2} &{} \dots &{} r_{GnK} \end{array}\right] \end{aligned}$$

#### Stage III: Personalized deconvolution

With the personalized reference panel $\varvec{R}_n$ available for each subject *n*, and the original bulk mixture transcriptome data, as shown in the lower-right corner of Fig. 1, we use *non-negative least squares* to deconvolute the cell type abundance $\varvec{\Theta }_{I,n}$. Here, we solve for $\varvec{\Theta }_{I,n}$ by optimizing the following objective function, for each subject *n*, under the constraint $\varvec{\Theta }_{I,n} \ge 0$:

$$\begin{aligned} \Vert \varvec{R}_n \otimes \varvec{I}_{tn} vec\left(\varvec{\Theta }'_{I,n}\right)- vec\left(\varvec{y}'_{n}\right) \Vert _2 \end{aligned}$$

$\varvec{\Theta }_{I,n}$ is of dimension $K\times t_n$ for each subject *n*. This is a joint optimization across all the samples per subject simultaneously instead of sample-wise optimization, using the subject-specific signature matrix $\varvec{R}_n$ and quadratic programming. The subscript *I* stands for the ***imply***-estimated cell type abundance, in contrast to the coarse deconvolution abundance $\varvec{\theta }_{E}$ from *Stage I*. Note that $\nu$-SVR with sample-wise optimization can also be utilized in this stage as an alternative approach, and we name this variant as ***imply-s***. Overall, instead of using the population-level signature matrix $\varvec{E}$, the key of ***imply*** is to adopt a personalized $\varvec{R}_n$ to serve in the cell type abundance inference.

### Simulations

#### Pure cell-type-specific expression profiles

Notations of gene *g*, subject *n*, sample *i*, and cell type *k* are borrowed from the previous section. The simulation scheme is borrowed and adapted from on our prior benchmark study [39], offering a comprehensive and flexible simulation framework. We utilized a set of true cell line RNA-seq dataset [34] to obtain the distribution of gene expression parameters in a genome-wide scale. This study has six immune cell types (neutrophils, monocytes, B-cells, CD4 T cells, CD8 T cells, and natural killer cells). For each cell type, the CTS gene expression parameters, expression means ($\mu _{gk}$) and biological dispersion ($\phi _{gk}$), are obtained by using the *PROPER* [53] package. There are correlations across cell type for both expression means and dispersion, as expected. Therefore, for the reference panel simulation, we use *Multivariate Normal Distribution* (MVN) to capture correlations for both expression mean and dispersion, in the log scale. We use $\hat{\varvec{\Sigma }}_{m}$ ($\bar{\varvec{\mu }}_m$) and $\hat{\varvec{\Sigma }}_{\phi }$ ($\bar{\varvec{\mu }}_\phi$) to denote variance-covariance matrices of expression mean and dispersion, respectively. The dimensions match the number of cell types and the details of $\hat{\varvec{\Sigma }}_{m}$, $\bar{\varvec{\mu }}_m$, $\hat{\varvec{\Sigma }}_{\phi }$, and $\bar{\varvec{\mu }}_\phi$ can be found in the Additional file 1: Simulation Details. We conduct 30 iterations for each simulation scenario, with six cell types and 1,000 genes:

$$\begin{aligned} \varvec{M}{} & {} \sim MVN\left(\bar{\varvec{\mu }}_m,\hat{\varvec{\Sigma }}_{m}\right)\\ \varvec{\Phi }{} & {} \sim MVN\left(\bar{\varvec{\mu }}_\phi ,\hat{\varvec{\Sigma }}_{\phi }\right) \end{aligned}$$

Note that the mean expression $\varvec{M}$, and the biological dispersion $\varvec{\Phi }$ are still parameter matrices for downstream usage. The case and control groups share the same $\varvec{\Phi }$, but distinct mean expressions. The effect size of differential expression is defined by Log-Fold-Changes (LFC) denoted by $\Delta$. The means for control and case are denoted by $\varvec{M}_{Ctrl}=\varvec{M}$ and $\varvec{M}_{Case}=\varvec{M}+\Delta$. We introduce 10% of differentially expressed (DE) genes on cell types 1, 2, 3, and 4, respectively. The true CTS gene expression matrix $\varvec{P}$ is derived from a *Gamma Distribution* for both case and control:

$$\begin{aligned} \varvec{P}_{case/ctrl} \sim \Gamma \left( \frac{1}{\exp (\Phi )}, \exp (\varvec{M}_{case/ctrl}) \times \exp (\varvec{\Phi }) \right) \end{aligned}$$

Subject-to-subject variations (SSV) are also introduced, implemented as the expression change percentages over the baseline in $\varvec{P}_{case/ctrl}$. Variations are then added to $\varvec{P}_{case/ctrl}$ to obtain subject-specific underlying gene expression matrices $\varvec{P}_n$. To reflect the various levels of variations, the level of SSV can take the following ranges: 0-5%, 5%-10%, 10%-20%, and 20%-50%. The total subject count per case/control group can take value in 25, 50, 75, and 100. The subject-level cell-type-specific underlying gene expression is shared across multiple samples, and each subject is measured 3 times.

**Fig. 2:**
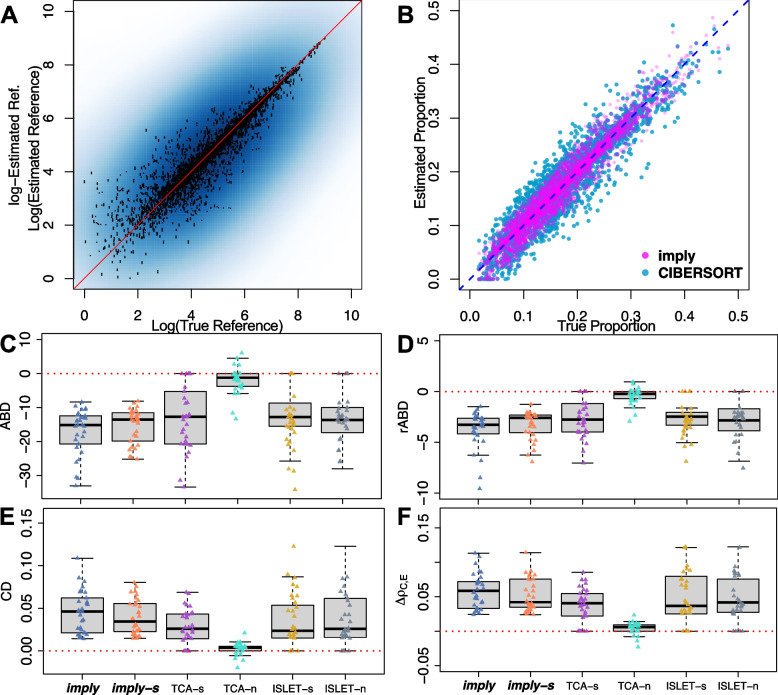
***imply*** can improve cell type deconvolution accuracy. **A** Scatterplot showing ***imply*** estimated gene expression reference panel versus the true reference panel values. **B** Superimposed scatterplot of the ***imply***-estimated cell type proportion over the CIBERSORT-estimates, which are the results from the current state-of-art method. ***imply*** shows better concordance with the ground truth. **C**-**F** Boxplots displaying evaluation metrics and each point representing one simulation iteration: *ABD*, *rABD*, *CD*, and $\Delta \rho _{\mathrm{C,E}}$. Five additional modeling frameworks are benchmarked. The red dashed line (value of 0) represents no improvement in proportion estimation. For (**C**) and (**D**), lower values indicate better deconvolution accuracy. For (**E**) and (**F**), higher the better

#### Cell type proportions and observed read counts

To generate the cell type proportions, we borrow information from multiple well-labeled single cell RNA-seq studies. We mix and bootstrap cell labels from a combined pool and obtain the empirical cell proportions from this resampling. We use *Dirichlet Distribution* to estimate $\varvec{\alpha }$ parameters and simulate cell type proportions. The detailed procedures for generating cell proportions are outlined in the Additional file 1: Simulation Details. The simulated sample-specific cell proportions are:

$$\begin{aligned} \varvec{\theta _{T,ni\cdot }} \sim Dirichlet(\varvec{\alpha }_{ctrl/case}) \end{aligned}$$

$\varvec{\theta _{T,ni\cdot }}$ are reorganized into cell composition matrix, $\varvec{\Theta }_T$. The sample-specific underlying gene expression reference panel is the weighted average across cell types in $\varvec{P}_n$ by $\varvec{\theta }_{T,ni\cdot }$, denoted as $\varvec{\lambda }_{ni} = \varvec{P}_n\times \varvec{\theta }_{T,ni\cdot }^{'}$, and will follow a *Gamma Distribution* as well [41]. $\varvec{\lambda }_{ni}$ is further assessed by the *Poisson Distribution* to generate observed RNA-sequencing counts data, denoted as:

$$\begin{aligned} \varvec{y}_{ni} \sim Pois(\varvec{\lambda }_{ni}), \end{aligned}$$

for subject *n* at measurement *i* across all *G* genes. Overall, the *Gamma Distribution* models biological variations, the *Dirichlet Distribution* regulates cell type proportion variations, and *Poisson Distribution* mimics technical noise related to the randomness in the sequencing experiments. This multi-step simulation design enables the separation of biological and technical noise [16, 39], among other factors, to facilitate a comprehensive simulation study for our model testing.

$$\begin{aligned} \varvec{\Theta }_T= \left[ \begin{array}{cccc} \theta _{T,111} &{}\theta _{T,112}&{}\dots &{} \theta _{T,11K}\\ \theta _{T,121} &{}\theta _{T,122}&{}\dots &{} \theta _{T,12K}\\ \vdots &{} &{} \ddots &{} \\ \theta _{T,1t_{1}1} &{}\theta _{T,1t_{1}2}&{}\dots &{} \theta _{T,1t_{1}K}\\ \vdots &{} &{} \ddots &{} \\ \theta _{T,N11} &{}\theta _{T,N12}&{}\dots &{} \theta _{T,N1K}\\ \theta _{T,N21} &{}\theta _{T,N22}&{}\dots &{} \theta _{T,N2K}\\ \vdots &{} &{} \ddots &{} \\ \theta _{T,Nt_{N}1} &{}\theta _{T,Nt_{N}2}&{}\dots &{} \theta _{T,Nt_{N}K} \end{array}\right] \end{aligned}$$

#### Input signature matrix

The signature matrix is required by the algorithm as an input. To obtain it, we first take the average across all $\varvec{P}_n$ matrices to get CTS gene expression mean matrix. Then 300 or 500 pseudo-marker genes are selected by *findRefinx* function (ordered by coefficients of variation) from TOAST [32] to establish a signature matrix as the input for ***imply***.

### Evaluation metrics

We use $\varvec{\Theta }$ and $\hat{\varvec{\Theta }}$ to denote the ground truth and estimated cellular abundances, which has the unique property of unit-sum and bounded by zero and one. Naturally, a central goal here is to assess how good the cellular abundances estimator $\hat{\varvec{\Theta }}$ is. Specifically, we denote ***imply***’s deconvolution values as $\hat{\varvec{\Theta }}_I$, and existing method’s deconvolution results as $\hat{\varvec{\Theta }}_E$. The existing methods include currently available deconvolution approaches and those do not consider personalized reference panels. The following evaluation metrics are adopted for benchmarking:

#### Absolute bias differences (ABD) and relative absolute bias differences (rABD)

 

$$\begin{aligned} ABD{} & {} := \sum | \hat{\varvec{\Theta }}_I - \varvec{\Theta } | - \sum | \hat{\varvec{\Theta }}_E - \varvec{\Theta } |, \\ rABD{} & {} := \left[ \varvec{Avg}\left( \frac{|\hat{\varvec{\Theta }}_I - \varvec{\Theta }|}{\varvec{\Theta }}\right) - \varvec{Avg}\left( \frac{|\hat{\varvec{\Theta }}_E - \varvec{\Theta }|}{\varvec{\Theta }}\right) \right] \times 100\% \end{aligned}$$

Here, for both *ABD* and *rABD*, if they are smaller than zero, it means the ***imply*** successfully reduces the estimation bias. A smaller value further indicates better performance.

#### Correlation differences (CD)

 

$$\begin{aligned} CD := corr\left( \hat{\varvec{\Theta }}_I,\varvec{\Theta }\right) - corr\left( \hat{\varvec{\Theta }}_E,\varvec{\Theta }\right) \end{aligned}$$

Here, if *CD*>0, then ***imply*** increases the correlation between the estimation and the ground truth. A larger value indicates favorable performance.

#### Lin’s concordance correlation coefficient (CCC) and its variations

Lin’s concordance correlation coefficient (Lin’s CCC) [31], denoted as $\rho _{\mathrm{C}}$, has been extensively used to evaluate the concordance between a new measure and a gold standard measurement, and is defined as:

$$\begin{aligned} \rho _{\textrm{C}} \left( \varvec{\Theta }, \hat{\varvec{\Theta }}\right) = 1-\frac{E\left[ \left( \varvec{\Theta } - \hat{\varvec{\Theta }}\right) ^2\right] }{E_I\left[ \left( \varvec{\Theta } - \hat{\varvec{\Theta }}\right) ^2\right] }, \end{aligned}$$

where $E_I$ indicates the expectation under the assumption that $\varvec{\Theta }$ and $\hat{\varvec{\Theta }}$ are independent. Lin’s CCC is bounded between 1 (perfect agreement) and -1 (disagreement), and the concordance improves as $\rho _{\textrm{C}} (\varvec{\Theta }, \hat{\varvec{\Theta }})$ approaches 1. Additionally, we adopt a Euclidean distance-based variation of Lin’s CCC, by substituting the expected squared difference to Euclidean distance, denoted as $\rho _{\textrm{C,E}}$, defined below:

$$\begin{aligned} \rho _{\textrm{C,E}} \left( \varvec{\Theta }, \hat{\varvec{\Theta }}\right) = 1-\frac{ E\left[ \sum _{k=1}^{K} \left( \varvec{\Theta }^{(k)} - \hat{\varvec{\Theta }}^{(k)}\right) ^2\right] }{ E_I\left[ \sum _{k=1}^{K} \left( \varvec{\Theta }^{(k)} - \hat{\varvec{\Theta }}^{(k)}\right) ^2\right] } \end{aligned}$$

Another option is to employ the Aitchison [2] distance-based Concordance Correlation Coefficient (CCC), which is explained in detail in the Additional file 1: Evaluation Metric, with the results provided in the Additional file 1: Simulation Results. These metrics are adopted because they have been shown to be statistically more rigorous in dependent measures that are subject to the positiveness and unit-sum constraints [13], as is often the case in compositional proportion outcome. If ***imply*** yields increased concordance and improved precision, we would expect positive values in the differences of CCC. These metrics are respectively defined below:

$$\begin{aligned} \Delta \rho _{\textrm{C}}{} & {} = \rho _{\textrm{C}} \left( \varvec{\Theta }, \hat{\varvec{\Theta }}_I\right) - \rho _{\textrm{C}} \left( \varvec{\Theta }, \hat{\varvec{\Theta }}_E\right) \\ \Delta \rho _{\textrm{C,E}}{} & {} = \rho _{\textrm{C,E}} \left( \varvec{\Theta }, \hat{\varvec{\Theta }}_I\right) - \rho _{\textrm{C,E}} \left( \varvec{\Theta }, \hat{\varvec{\Theta }}_E\right) \end{aligned}$$

### Overview of the PDBP and TEDDY cohorts

Real data analysis was conducted on two cohorts: the Parkinson’s disease Biomarker Program (PDBP) and The Environmental Determinants of Diabetes in the Young (TEDDY) [29]. The PDBP consortium has the repeatedly measured RNA-seq datasets, demographic and clinical information collected from patients with or without Parkinson’s Disease (PD) recruited from multiple medical centers and research institutions in the United States between November 2012 and August 2018. The PDBP cohort data were collected longitudinally overtime for each subject, allowing us to track changes in cell type composition and disease progression over time. In our study de-identified participants with at least three observations over time were retained. A total of 399 PD patients and 173 controls, with 2599 longitudinal samples over 2 years, were included. Longitudinal RNA samples in PDBP were extracted from the whole blood. Clinical data includes information about patients’ medical history, symptoms, disease status, total Montreal Cognitive Assessment (MoCA) scores, and MDS UPDRS part III motor scores. The TEDDY cohort is a multi-center pediatric study of Type 1 diabetes (T1D). TEDDY cohort screened and enrolled participants with susceptibility of T1D based on the Human Leukocyte Antigen (HLA) genotypes from six clinical centers in four countries (U.S., Finland, Germany, and Sweden). A total of 8,676 high-risk infants were enrolled from birth and followed every 3 months for blood sample collection and islet autoantibody (IAbs) measurement up to 4 years of age. Details of sample collection, RNA sequencing procedures, bioinformatics processing, and quality control are described in the Additional file 1: Method Details and [54]. The longitudinal whole blood transcriptome data enable the ***imply*** deconvolution.

## Results

We first evaluate ***imply***’s deconvolution accuracy using synthetic data generated through the steps described earlier. ***imply*** is the only method that re-estimates cell type proportions using subject-specific reference panels from longitudinal bulk data; therefore, a direct comparison with existing deconvolution methods is not directly available. Nevertheless, we designed the benchmark to be inclusive of existing methods. TCA [46], designed for csDE genes detection, integrates a re-estimation feature for refining initially noisy cell proportion inputs. Specifically, TCA takes a maximum-likelihood (ML) approach to derive model parameters given initial cell proportion, and then the proportions are subsequently updated based on these estimated parameters. TCA requires preliminary cell proportions for effective re-estimation. We employ the *non-negative least squares* and $\nu$-SVR to acquire the initial inputs for TCA, and label them as TCA-n and TCA-s, respectively, which could be benchmarked with ***imply***. ISLET [16] is the first method to retrieve individual-specific reference estimation in repeated samples based on the Expectation-Maximization (EM) algorithm. ISLET can be an alternative approach to our mixed-effect model to solve subject-specific reference panels. Here, we consider ISLET-s and ISLET-n, respectively, representing ISLET variants that the final personalized deconvolution is conducted by SVR or *non-negative least squares*, respectively. We also introduce a variant of ***imply***, where *Stage III* is achieved by SVR instead of the *non-negative least squares*. This variant is denoted as ***imply-s***. We comprehensively benchmark our proposed personalized deconvolution methods, ***imply*** and its variant ***imply-s***, against other algorithms: TCA-n, TCA-s, ISLET-n, and ISLET-s. Additionally, we compare ***imply*** with representative deep learning-based algorithms, Scaden [38] and TAPE [9], as well as popular statistical modeling methods, CIBERSORTx [44] and MuSiC [51], under a baseline simulation setting detailed in the Additional file 1: Figs. S27 and S28.

### ***imply*** increases precision in cell-type deconvolution

We start with a baseline simulation scenario with six cell types, two disease groups, and 100 subjects per group with 3 replicates per subject. The subject-specific variation (SSV) in the underlying CTS gene expression panels is up to 5%. To simulate csDE genes, we introduce 10% of DE genes respectively to cell types 1, 2, 3, and 4. The effect size is characterized by the Log-Fold-Change (LFC) set to 0.5. Figure 2A shows the estimated reference panels by ***imply*** versus the ground truth. Overall, we observe good accuracy in personalized reference panel recovery, especially among high-expression genes. This result demonstrates the fidelity of *Stage II* and lays a foundation for personalized deconvolution in *Stage III*. Next, we evaluate if ***imply***’s final cell type deconvolution, from *Stage III*, could reduce bias. Here, there are mainly two aspects to consider for accuracy benchmarking: one is to compare with alternative frameworks that do not use personalized reference panels; the other one is to benchmark with existing methods. Figure 2B shows the scatterplot of the estimated cell type proportions versus the true proportions. Our result is overlaid on top of the result from CIBERSORT, one of the state-of-the-art methods. ***imply*** yields higher precision in deconvolution as its estimates aggregate closer to the diagonal line. In Fig. 2C-F, the bias reductions are quantitatively assessed and compared using metrics introduced previously: *ABD*, *rABD*, *CD*, and $\Delta \rho _{\textrm{C,E}}$. Each point in a boxplot represents one simulation iteration, with the red dotted lines of zero indicating the basis for not using personalized reference panels. Thus, the zero line represents the existing deconvolution method, such as CIBERSORT, which did not consider personalized reference panels. For *ABD* and *rABD*, lower values indicate a greater increase in deconvolution accuracy; while for *CD* and $\Delta \rho _{\textrm{C,E}}$, higher values indicate improved concordance with the true values. Notably, ***imply*** consistently demonstrates the most substantial reduction in deconvolution bias and highest concordance with the truth. In contrast, TCA performs poorly, especially when the initial proportion inputs are estimated through *non-negative least squares* (TCA-n). Even when the initial proportion input is derived from CIBERSORT, the bias reduction achieved by TCA (TCA-s) is not as significant as that achieved by ***imply***. Furthermore, we notice that subject-specific reference panels estimated by ISLET also yield benefits for personalized deconvolution, illustrated by ISLET-s and ISLET-n. However, the improvements are not as pronounced as those achieved by ***imply***. The Wilcoxon signed-rank test was conducted to demonstrate the statistical significance of the superiority of ***imply*** compared to other models. Detailed test results can be found in the Additional file 1: Table S3-S6. Moreover, the Additional file 1: Figs. S27 and S28 present further benchmarking analysis conducted under slightly different setups, showing that ***imply*** outperforms CIBERSORTx [44], MuSiC [51], and two deep learning-based methods, Scaden [38] and TAPE [9], under the baseline simulation setup.

We also explore the methods’ performance under various simulation scenarios and summarize the results in Table 1. The table shows averaged *ABD*s across simulation replicates, with each standard error, at exhaustive combinations of subject-specific variations (SSV=0-5%, 5%-10%, 10%-20%), effect sizes (LFC = 0.5, 1, 1.25), and sample sizes (N=25, 50, 100). Bold fonts highlight the algorithm with the most amount of bias reduction for each scenario. ***imply*** and ***imply-s*** consistently demonstrate exceptional performance in reducing deconvolution bias across all scenarios.

**Table 1:** Benchmarking ***imply*** across various simulation scenarios. The table shows *Absolute Bias Difference* *(ABD)* at various subject-specific variations (SSV), effect sizes (LFC), and sample sizes (N). *ABD* values are shown, along with their standard error in parentheses. A lower value indicates better deconvolution estimation improvement. The bold font indicates the best method in each scenario

SSV	LFC	N	***imply***	***imply-s***	TCA-s	TCA-n	ISLET-s	ISLET-n
0%$\sim$5%	0.5	25	**-3.95 (1.92)**	-3.51 (1.56)	-2.21 (1.96)	-0.33 (0.85)	1.21 (20.15)	1.03 (20.25)
		50	**-8.97 (3.86)**	-7.97 (3.44)	-6.6 (4.92)	-0.8 (2.2)	5.76 (52.49)	7.75 (57.2)
		100	**-17.08 (6.66)**	-15.38 (5.11)	-13.1 (9.43)	-1.88 (3.97)	25.71 (148.83)	15.11 (116.56)
	1	25	**-3.39 (2.98)**	-3.17 (2.43)	-0.89 (2.57)	-0.29 (0.64)	14.95 (40.81)	14.51 (36.8)
		50	**-8.59 (4.88)**	-7.63 (4.78)	-1.64 (4.03)	-1.11 (1.92)	45.7 (84.85)	35.33 (70.75)
		100	-16.49 (10.45)	**-16.54 (11.56)**	-4.17 (10.17)	-2.29 (3.78)	22.55 (121.26)	19.26 (115.44)
	1.25	25	**-4.9 (3.11)**	-4.28 (3.48)	-1.38 (2.75)	-0.35 (0.8)	4.55 (22.45)	2.22 (18.2)
		50	**-9.39 (5.56)**	-9.09 (8.35)	-4.47 (8.67)	-0.96 (1.79)	31.72 (80.43)	24.19 (73.19)
		100	-21.73 (13.28)	**-21.91 (20.18)**	-9.56 (16.92)	-2.13 (3.36)	52.94 (144.77)	30.96 (102.83)
5%$\sim$10%	0.5	25	**-3.53 (1.36)**	-3.28 (1.18)	-1.88 (2.47)	-0.33 (1.52)	8.84 (37.92)	6.4 (31.77)
		50	**-7.6 (3.36)**	-6.79 (2.69)	-4.53 (5.13)	-0.51 (3.99)	2.88 (47.91)	-0.95 (29.74)
		100	**-15.64 (5.85)**	-15.42 (8.12)	-8.06 (11.59)	-1.9 (7.37)	7.15 (117.46)	5.51 (107.12)
	1	25	**-4.03 (2.45)**	-3.75 (2.39)	-0.22 (1.42)	0.06 (1.02)	15.35 (41.93)	16.46 (45.14)
		50	-7.93 (4.29)	**-8.01 (7.12)**	-0.45 (5.5)	-0.19 (2.94)	29.12 (79.78)	21.14 (68.21)
		100	**-15.86 (10.45)**	-13.97 (9.44)	-1.4 (11.86)	-0.35 (3.87)	57.17 (176.1)	58.45 (172.78)
	1.25	25	-4.21 (2.68)	**-6.55 (9.34)**	-0.65 (2.52)	-0.08 (0.61)	0.99 (23.14)	1.49 (13.97)
		50	**-9.85 (6.61)**	-8.83 (7.24)	-1.81 (6.96)	0.05 (2.35)	36.08 (83.23)	24.17 (63.91)
		100	-20.3 (12.05)	**-22.76 (24.38)**	-6.36 (18.9)	-1.44 (4.28)	28.14 (137.05)	21.45 (125.49)
10%$\sim$20%	0.5	25	**-3.27 (1.64)**	-2.83 (1.34)	-0.1 (4.84)	0.65 (3.79)	13.74 (41.37)	13.22 (40.18)
		50	**-6.48 (2.86)**	-6.14 (2.27)	1.63 (15.12)	2.07 (10.22)	2.26 (38.29)	2.66 (40.29)
		100	**-13.98 (5.5)**	-12.73 (5.14)	1.32 (31.49)	9.86 (29.37)	7.05 (94.67)	7.02 (103.21)
	1	25	-2.6 (3.46)	**-3.49 (4.91)**	0.75 (3.1)	2.01 (4.04)	28.61 (44.79)	27.28 (45.55)
		50	**-7.75 (4)**	-7.43 (3.35)	1.86 (7.61)	5.18 (11.03)	19.09 (64.13)	17.76 (64.18)
		100	-14.52 (8.4)	**-14.8 (8.49)**	6.67 (19.22)	11.3 (26.41)	46.52 (124.95)	51.84 (140)
	1.25	25	**-4.77 (1.99)**	-4.22 (1.59)	0.41 (3.07)	1.98 (3.19)	12.72 (35.35)	6.78 (23.43)
		50	**-9.49 (4.57)**	-8.68 (4.45)	0.17 (6.14)	4.08 (7.69)	27.71 (73.1)	30.77 (84.76)
		100	**-19.13 (10.37)**	-16.49 (12.37)	0.89 (18.5)	8.69 (18.58)	-8.27 (16.34)	-9.42 (13.32)

**Fig. 3:**
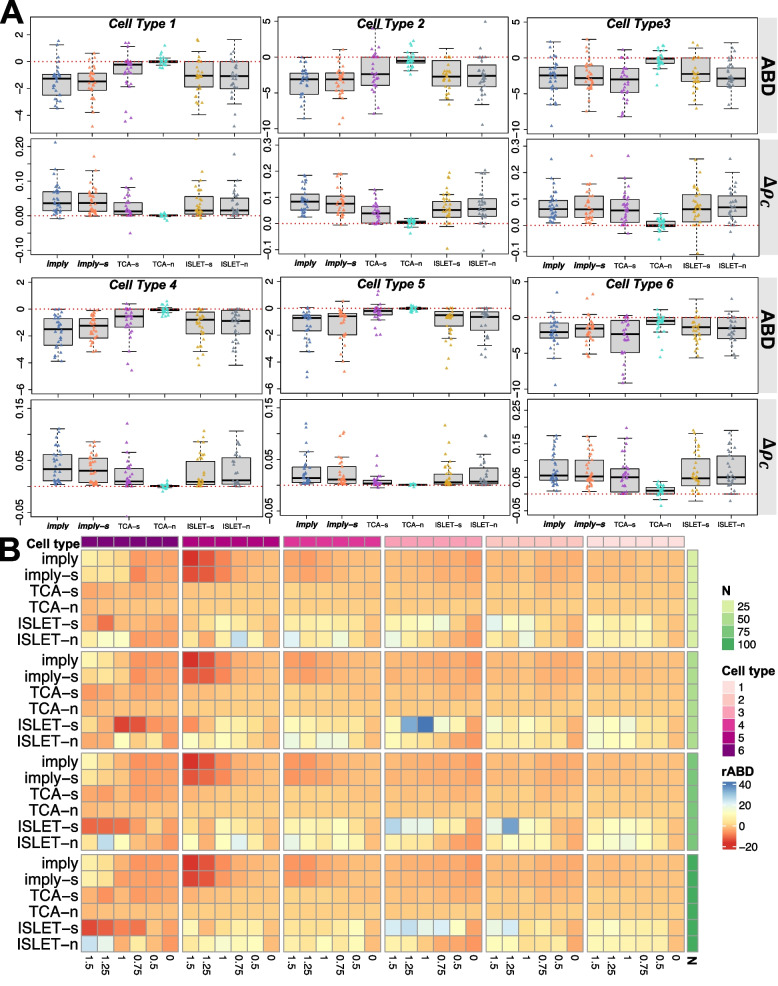
Cell-type resolution improvements in proportions estimated by ***imply***. **A** Boxplots showing *ABD* (upper panel) and $\Delta \rho _{\textrm{C}}$ (lower panel), for cell types 1 to 6. **B** Heatmap showing the deconvolution improvement using the *rABD* metric, aggregated by cell types (top row) and sample sizes (right column), for various effect sizes (bottom row)

### Benchmarking at cell-type resolution

We next investigate the deconvolution accuracy at each cell type. Figure 3A shows the *ABD* and $\Delta \rho _{\textrm{C}}$ outcomes of 30 replicates of each cell type under the condition of the SSV range of 0-5%, the sample size of 75, and the effect size of 0.5. Across all cell types, we can see a discernible reduction in bias when personalized reference panels are adopted. ***imply*** and ***imply-s*** consistently stand out, yielding a significant enhancement in concordance within each cell type compared to other models. The heatmap in Fig. 3B shows the average *rABD* at various combinations of sample sizes and effect sizes, separated by cell types. At large effect sizes, improvements in cell deconvolution accuracies facilitated by ***imply*** are notably more profound. However, *rABD* exhibits limited alterations to variations in sample sizes. The simulation results also suggest a connection between bias reduction and cell type abundances; specifically, deconvolution accuracies for more abundant cells are highly sensitive to LFC changes (see the Additional file 1: Simulation Results for additional details). In contrast, for minor cell types, the small amount of contribution makes deconvolution an even more challenging task, where the sequencing noise could easily dominate underlying biological variations. The Additional file 1: Fig. S31 contains additional simulation results specifically addressing minor cell types.

### Influential factors in deconvolution accuracy

**Fig. 4:**
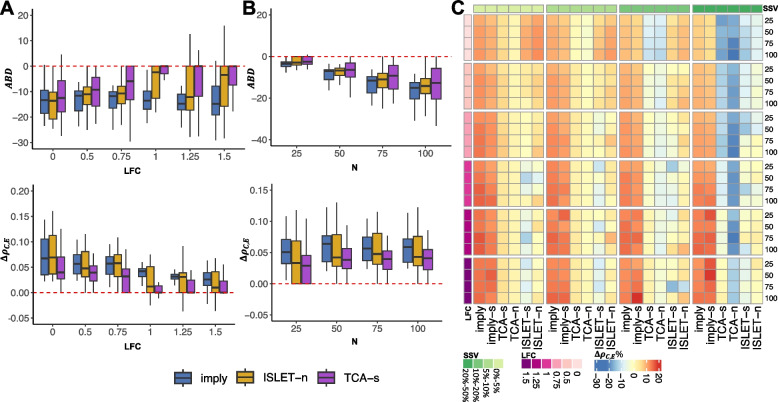
Effect size, sample size, and subject-specific variation affect deconvolution accuracy. **A** Boxplots of *ABD* (upper panel) and $\Delta \rho _{\textrm{C,E}}$ (lower panel) across three methods: ***imply***, ISLET-n, TCA-s, under different effect sizes (LFC): 0, 0.5, 0.75, 1, 1.25, and 1.5. **B** Similar to **A** but across various sample sizes per group: 25, 50, 75, 100. **C** Heatmap showing the relative $\Delta \rho _{\textrm{C,E}}$ across various combinations of sample sizes, effect sizes, and SSV. The color bars on the left and the top indicate the LFC and SSV, respectively. The number on the right indicates the sample size per group

We further zoom in to study how sample size, effect size, and subject-specific variation would affect personalized deconvolution. In Fig. 4A, *ABD* and $\Delta \rho _{\textrm{C,E}}$ for ***imply***, together with ISLET-n and TCA-s, are presented across LFC ranging from 0 (null) to 1.5. ***imply*** consistently exhibits the lowest *ABD* in all scenarios and the highest $\Delta \rho _{\textrm{C,E}}$ in most settings. These results indicate the advantage of adopting personalized reference panels. In addition, ***imply*** provides the most stable (i.e., smallest variation) among the three methods as the effect size increases. Figure 4B shows the same metrics across various sample sizes. As expected, *ABD* decreases as the sample size increases. ***imply*** consistently maintains the highest $\Delta \rho _{\textrm{C,E}}$ across various sample sizes. In Fig. 4C, we further investigate the $\Delta \rho _{\textrm{C,E}}$ alteration percentages, which are defined as $\Delta \rho _{\textrm{C,E}}\% = \frac{\Delta \rho _{\textrm{C,E}}}{\rho _{\textrm{C,E}}(\Theta _E,\Theta )}\times 100\%$, at different levels of SSV, which are annotated by the top row. We observe a robust pattern across different effect sizes, samples sizes, and SSVs, and conclude that ***imply*** and ***imply-s*** consistently provide the most outstanding concordance improvement.

### Application of ***imply*** to longitudinal transcriptomic datasets

**Fig. 5:**
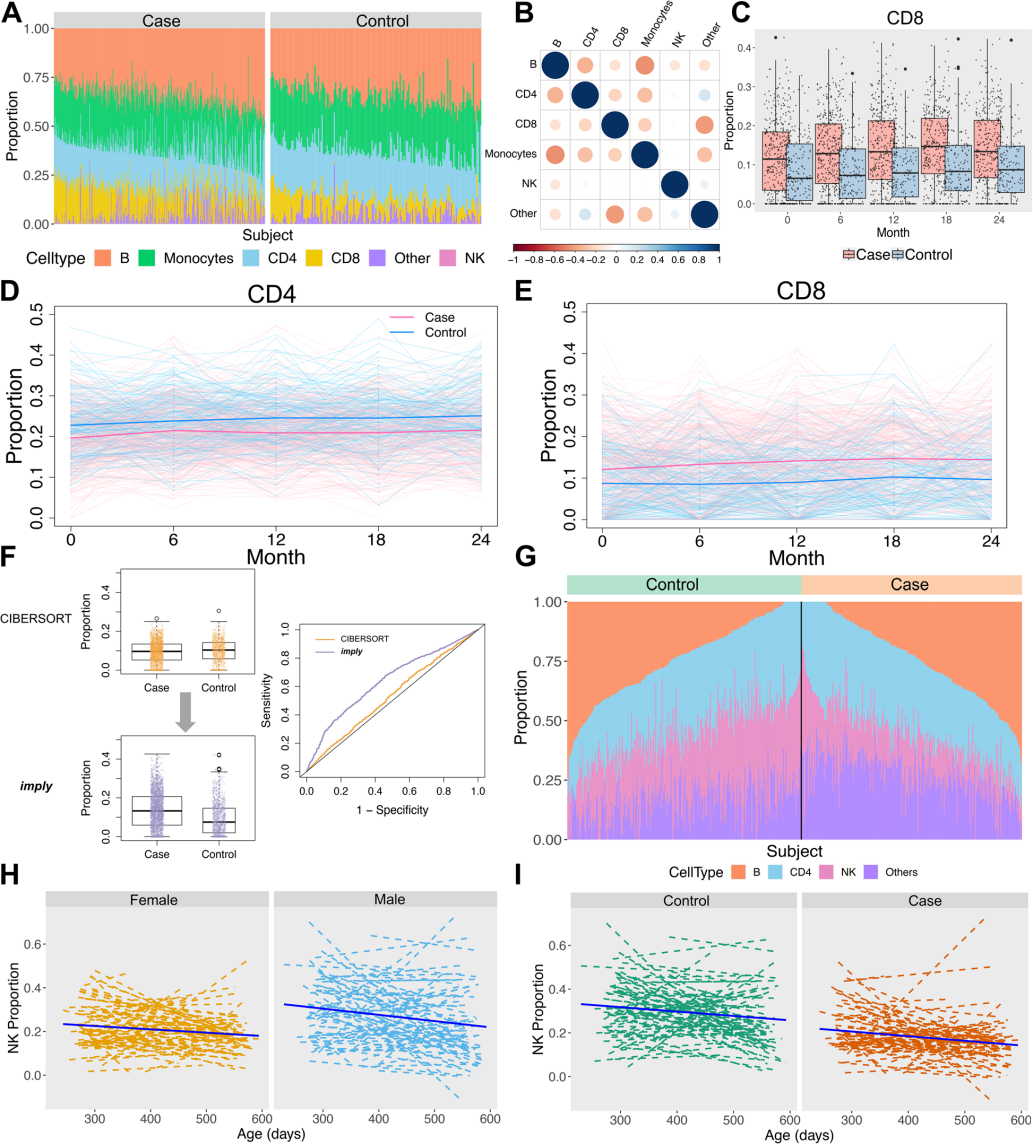
Phenotype-associated cell type disparities from PDBP and TEDDY consortia. PDBP: Parkinson’s Disease Biomarker Program. TEDDY: The Environmental Determinants of Diabetes in the Young. **A** Cell type proportions for all subjects, separated by Parkinson’s disease (PD) status in PDBP dataset. The bar represents the mean cell type proportions across all visit times, for each subject. **B** Pearson correlations of cell type proportions between six cell types, among all individuals. **C** Distribution comparisons of CD8 cell type proportions between PD cases and controls, at each visit time. **D** Deconvoluted CD4 cell type proportions along with study participants’ visit time. PD cases (pink) and controls (blue) are illustrated by both individual background lines (thin) and foreground lines (thick). **E** Same as in (**D**) but for CD8 cell type proportions. **F** Grand comparison of CD8 cell type proportions between PD cases and healthy controls, using CIBERSORT and our method ***imply***. Results from ***imply*** show a larger effect size, more significant test statistics, and increased discriminative capacity. **G** Cell type proportions for all subjects, separated by pancreatic islet autoantibodies (IA) status, in TEDDY dataset. The bar represents the mean cell type proportions across all visit times, for each subject. **H** NK cell proportions along infant’s age (in days) at sample collection, for female and male subjects. Average fitted lines (solid) overlay individual-specific lines (dashed). **I** Same as in (**H**) but separated by IA case and control status

We applied ***imply*** to analyze the longitudinal transcriptomic datasets from both the PDBP and TEDDY [29] consortia. For PDBP dataset, the mean proportions across all visit times of six cell types, including B cell, Monocyte, CD4, CD8, NK cell, and other cells, are shown for cases and controls in Fig. 5A. Here, B cell contributes the most among all six cell types, while NK contributes the least. The visualization suggests a higher CD8 proportions in the PD group than in the control group, while CD4 proportions in the PD groups are lower. Figure 5B displays the heatmap of Pearson correlations among the six cell types. B cells, monocytes, and CD4 all show negative pairwise correlations. Figure 5C shows boxplots of CD8 cell type proportions comparing case and control, at each time point. The median value of CD8 proportion in case is higher than that in control group at each time point. The CD4 and CD8 cell type proportions, broken down by the participant’s visit time of each subject, are shown in Fig. 5D and E, respectively. For CD4 cell type, the mean proportions in case group are lower than those in control group for each visit time. For CD8 cell type, the mean proportions among cases are higher than those among controls, for each visit time. These findings are well-aligned with previous studies where the PD patients showed elevated CD8 proportions and reduced CD4 proportions than controls [7, 20, 26, 52]. We also benchmarked ***imply*** with the existing method CIBERSORT as shown in Fig. 5F. Using CIBERSORT, the *p*-value of the Wilcoxon Rank Sum test is 0.0111 and the median difference is $-0.007$ for CD8 proportions, between cases and controls. It incorrectly suggests that the CD8 cell type proportion of cases are lower than controls. In contrast, ***imply*** yields a *p*-value less than $10^{-16}$ and the median difference is 0.58, which shows the correct effect size direction. It also increases differential power between cases and controls, as shown in the ROC plot. We also explored the associations between the various cell type proportions and clinical outcomes, including total UPSIT score, total scores of Montreal Cognitive Assessment (MoCA), and MDS UPDRS part III motor scores, which provide additional assessments of patient’s cognitive and motor function in PD. Additionally, association studies with Cerebrospinal fluid (CSF) were conducted (the Additional file 1: Fig. S21-S25). For the T1D study of TEDDY, the disease status (i.e., cases) of interest is the onset of pancreatic islet autoantibodies (IA). The longitudinal analysis of re-quantified cellular composition identifies NK cell abundance as higher in males than females ($p<0.0001$), as illustrated in Fig. 5H. Previous research in TEDDY reported a higher risk of IA being associated with viral infection during the first 6 months of life [50]. The sex difference in NK cell fraction in Fig. 5H could be a consequence of early-life vaccination or viral infection [10], since infants are exposed to exogenous antigens and have a high susceptibility to infections. In this analysis, we use longitudinal samples of IA cases and controls collected at the age of 9-21 months, and compare deconvoluted cell fractions between groups by ***imply***. Figure 5I shows that the NK cell proportions are significantly lower ($p<0.0001$) in the participants who developed IA at a young age compared to controls, while this trend is not observed in the initial cell abundance estimated by CIBERSORT ($p=0.77$, the Additional file 1: Fig. S26). The relative higher NK cell abundance in males (vs. females) and controls (vs. cases) among TEDDY participants is consistent with the previous finding that males have a lower risk of autoimmunity than females [37].

Furthermore, we perform a downstream csDE genes analysis on IA status based on the ***imply***-deconvoluted cell type fraction, using ISLET [16] with FDR$<0.1$. The cell type proportions improved by ***imply*** enabled the detection of DE genes in CD4 T cells and identified more NK-cell-specific DE genes ($n>300$) compared to a previous csDE genes testing result ($n=30$) based on the proportions deconvoluted by AutoGeneS [3]. The IA-csDE genes based on the improved cell fractions include the markers for multiple T cell receptors (e.g., TRBV, TRDV, TRGV, TRJV) and the genes regulating immune responses such as *CAMP* and *CRK*. The *CAMP* gene expression was found to be associated with serum levels of vitamin D in the studies of innate immunity [14, 24, 36], while the TEDDY cohort also reported a strong linkage between vitamin D and the risk of IA [33]. Protein *CRK* is involved in NK cells inhibitory receptor signaling and modulates the signaling of activating receptors, which may function as a two-way molecular switch to control NK cell-mediated cytotoxicity [35, 42].

## Discussion

The computational deconvolution of admixed bulk tissue samples is drawing substantial interest in *-omics*. The interest is growing as deconvolution methodologies are being developed, and as increasingly large datasets are becoming available with and without repeated measures. We are among the first to consider personalized reference panels in deconvolution. Our computational framework optimizes the usage of shared information in longitudinal samples from each subject. Alternative machine learning approaches, such as Expectation-Maximization (EM) and non-negative matrix factorization algorithms, could also extract personalized reference panels and have been implemented in ISLET [16] and CIBERSORTx [44]. Nevertheless, these methods lack the conciseness and computational efficiency exhibited by the proposed linear mixed-effects modeling framework.

A limitation of ***imply*** is the requirement of an initial signature matrix as the input in *Stage I*, which could affect the initial cell type abundance estimation as the input for downstream. An alternative approach is to initialize cell fractions by external multi-subject reference cell count data, such as single-cell profiling and labeling, flow cytometry, or imaging. For some genes, the random effect variance estimation may shrink towards zero, likely due to the adoption of penalized MLE. For such scenarios, the CTS heterogeneity between individuals would not be fully recovered. Furthermore, the intra-individual heterogeneity was not considered in reference panel recovery. This is because our present work was motivated by the bulk transcriptome of longitudinal blood samples, many of which were collected from healthy controls. In those scenarios, the underlying pure gene expression panel for each subject is relatively stable over time. Our previous work [16] suggests that the intra-individual CTS heterogeneity, when assessing using longitudinal PBMC scRNA-seq data, is trivial when compared with inter-individual variation. Hence, our future work will include the curation of longitudinal scRNA-seq data from distinct tissue types or disease populations and the incorporation of potential variations between time points at cell type resolution.

## Conclusions

In this work, we present our statistical framework ***imply*** to conduct cell-type deconvolution in bulk data using personalized panels. Our method ***imply*** leverages the repeated bulk RNA-seq samples to purify personalized reference transcriptome, and then jointly quantifies the cell abundances across multiple samples per individual. We show the advantage of using personalized reference panels by extensively *in silico* simulation studies and the analytical results of two large-scale longitudinal consortia. ***imply*** can produce more accurate and realistic deconvolution results.

### Supplementary Information

**Additional file 1.** ***imply***: improving cell-type deconvolution accuracy using personalized reference profiles, Supplementary Materials. It includes detailed methodological descriptions, simulation details, evaluation metrics, and results, as well as analyses on real data sets.

## Data Availability

1. ***imply*** is implemented and integrated into a R/Bioconductor package ISLET [17], which is available at https://bioconductor.org/packages/ISLET. 2. The PDBP bulk transcriptome and related clinical data are publicly available on request to AMP-PD at https://amp-pd.org 3. The TEDDY [29] bulk transcriptome dataset has been deposited in NCBI’s database of Genotypes and Phenotypes (dbGaP) with the primary accession code phs001442.v3.p2

## Abbreviations

CTS: Cell-Type-Specific
PDBP: Parkinson’s Disease Biomarkers Program
TEDDY: The Environmental Determinants of Diabetes in the Young
TCGA: The Cancer Genome Atlas
RB/RF: Reference-Based/Reference-Free
(cs)DE: (cell-type-specific) Differentially Expressed
scRNA-seq: single-cell RNA-seq
SSV: Subject-to-Subject Variations
(r)ABD: (Relative) Absolute Bias Differences
CD: Correlation Differences
Lin’s CCC: Lin’s Concordance Correlation Coefficient
LFC: Log-Fold-Change
MLE: Maximum Likelihood Estimation
SVR: Support Vector Regression
EM: Expectation-Maximization
