# Supplementary material for: imply: improving cell-type deconvolution accuracy using personalized reference profiles
Source: Genome Med. 2024 Apr 29;16:65. doi: 10.1186/s13073-024-01338-z (PMC11057104; doi:10.1186/s13073-024-01338-z)
Supplement: Supplementary file 1 — Additional file 1. imply: improving cell-type deconvolution accuracy using personalized reference profiles, Supplementary Materials. It includes detailed methodological descriptions, simulation details, evaluation metrics, and results, as well as analyses on real data sets. [file 13073_2024_1338_MOESM1_ESM.pdf]

imply: improving cell-type deconvolution accuracy using personalized reference profiles

Supplementary Materials

Guanqun Meng, Yue Pan, Wen Tang, Lijun Zhang, Ying Cui,
Fredrick R. Schumacher, Ming Wang, Rui Wang, Sijia He, Jeffrey Krischer,
Qian Li*, and Hao Feng*

Contents

1 Method Details **3**
1.1 ν-SVR Objective Function Constrains . . . 3
1.2 Mixed Effect Model Design Matrices . . . 3
1.3 PDBP and TEDDY Consortia Overview . . . 5

2 Simulation Details **7**
2.1 Real Data Selection and Cell-Specific Underlying Parameters' Estimation . . . 7
2.2 Cell mixture proportion generation . . . 8

3 Evaluation Metric **9**

4 Simulation Results **10**
4.1 Absolute Bias Difference (ABD) . . . 11
4.1.1 Cell-type-specific ABD . . . 14
4.2 Relative absolute bias difference ($rABD\%$) . . . 15
4.2.1 Cell-type-specific $rABD\%$ . . . 18
4.3 Correlation Differences (CD) . . . 19
4.4 Lin's CCC ($\Delta\rho_{\mathrm{C}}$) . . . 22
4.5 Euclidean Based-CCC ($\Delta\rho_{\mathrm{C,E}}$) . . . 25
4.6 Aitchison Based-CCC ($\Delta\rho_{\mathrm{C,A}}$) . . . 28

5 Real Data Analysis **31**
5.1 PDBP: Parkinson's Disease Biomarker Program . . . 31
5.2 TEDDY: The Environmental Determinants of Diabetes in the Young. . . . 36

6 Additional Benchmark **37**

7 Statistical Test **39**

8 Impact of Absent Cell Type on *imply*'s Performance **41**

9 Iterate *imply* to Further Reduce Bias **42**

10 Impact of Cellular Abundance on *imply*'s Performance **43**

11 Impact of Subject Sample Size on *imply*'s Performance **44**

12 Benchmark Algorithms on PBDP and TEDDY Data **45**

13 Benchmark Computational Time and Resources **47**

1 Method Details

1.1 ν-SVR Objective Function Constrains

The ν-SVR has been shown to work well in well-received deconvolution algorithms (Newman et al. [2015, 2019]) and the proportion of support vectors could be manually tuned by ϵ rooted into the objective function (main Figure 1). The support vectors are those observations outside the ϵ-tube (main Figure 1), which determine the hyperplane boundaries fitting the maximum number of observations (Schölkopf et al. [2000]). Also, observations within the ϵ-tube receive no penalty in the loss function. The objective function (in main text) is subject to the constraints, $\forall j$:

$$\begin{aligned} \boldsymbol{y}_{\cdot ni} - f(\boldsymbol{\theta}_{E,ni\cdot}) &\leq \epsilon + \xi_j \\ f(\boldsymbol{\theta}_{E,ni\cdot}) - \boldsymbol{y}_{\cdot ni} &\leq \epsilon + \xi_j^* \end{aligned}$$

Here, ϵ and C are tunable margin and positive constant which help to prevent overfitting by controlling the penalties caused by observations outside the ϵ-tube (Figure 1). ξ_j and ξ_j^* are slack variables denoting deviance from the margin and introducing extra error spaces to satisfy the potential infeasible but mandatory constraints. The concept is like a soft-margin concept in support vector machine (SVM) classification. The loss function ignores the prediction errors within ϵ-tube and is defined:

$$L_\epsilon = \begin{cases} 0 & \text{, if } |(\boldsymbol{y}_{\cdot ni})'_j - f(\boldsymbol{\theta}_{E,ni\cdot})_j| < \epsilon \\ |(\boldsymbol{y}_{\cdot ni})'_j - f(\boldsymbol{\theta}_{E,ni\cdot})_j| - \epsilon & \text{, otherwise} \end{cases}$$

1.2 Mixed Effect Model Design Matrices

The linear mixed effect modeling is employed to retrieve a subject- and cell-type-specific reference panel in *Stage II*. The modeling set up in the Additional file 1 uses a vectorized approach across all samples for each gene g, which slightly different from the main manuscript. In the main manuscript, the model is configured for a specific sample per gene g and the estimates are scalar rather than vector. Using the cell-type-specific and sample-specific proportions $\hat{\theta}_{E,nik}$ from *Stage I*, we can set up the following linear mixed-effect regression for each gene g:

$$\boldsymbol{y}_{g\cdot\cdot} = \boldsymbol{X}\boldsymbol{\beta}_g + \boldsymbol{A}\boldsymbol{u}_g + \boldsymbol{\epsilon}_g$$

$\boldsymbol{\epsilon}_g \sim N(\boldsymbol{0}, \sigma_0^2\boldsymbol{I})$ are the residuals and $\boldsymbol{y}_{g\cdot\cdot}$ stands for the vector of observed expression data for a specific gene g for all T samples. Here $\boldsymbol{X}$ and $\boldsymbol{A}$ are the design matrices (shown below, each with dimension $T \times 2K$ and $T \times NK$) for the fixed-effect $\boldsymbol{\beta}_g$ and the random-effect $\boldsymbol{u}_g$, respectively. The initial cell type abundance information is further reorganized into vectors $\boldsymbol{a}_{nk} = (\hat{\theta}_{E,n1k}, \hat{\theta}_{E,n2k}, \ldots \hat{\theta}_{E,nt_nk})'$. The fixed-effect $\boldsymbol{\beta}_g = (m_1, m_2, \ldots m_K, \beta_1, \beta_2, \ldots \beta_K)'$ has two components: $(m_1, m_2, \ldots m_K)$ are the baseline average cell-type-specific gene expression in the control group, and $(\beta_1, \beta_2, \ldots \beta_K)$ are the '*difference*' between the case group and the control group at cell type level. Note that our modeling allows for the incorporation of subject-level covariates such as disease status, for example, $z_n = 1$ for disease versus $z_n = 0$ for normal. The random-effect $\boldsymbol{u}_g = (u_{11}, u_{21}, \ldots u_{N1}, u_{12}, u_{22}, \ldots u_{N2}, \ldots, u_{1K}, u_{2K}, \ldots u_{NK})'$ captures the subject-level and cell-type-specific gene expression deviation from the group-level average. $\hat{\boldsymbol{\beta}}_g$ and $\hat{\boldsymbol{u}}_g$ can be solved by penalized least square algorithm with restricted maximum likelihood.

The subject- and cell-type-specific reference panel is obtained by combining $\hat{\boldsymbol{\beta}}_g$ and $\hat{\boldsymbol{u}}_g$ (fixed effect + random effect), with respect to each corresponding condition, cell type, and subject, as detailed in the main manuscript. We further reorganize reference panel into a subject-level structure and use $\boldsymbol{R}_n$ with dimension $G \times K$ to represent the subject- and cell-type-specific reference panel. Each element in $\boldsymbol{R}_n$ could be retrieved as: $r_{gnk} = \hat{m}_{k,g} + z_n\hat{\beta}_{k,g} + \hat{u}_{n,k,g}$.

As shown below, $\boldsymbol{X}$ and $\boldsymbol{A}$ each represents design matrix for fixed effect and random effect. The design matrix represents a typical and basic scenario where subjects originates from two groups in a case-control comparison setting. We are also interested in modeling gene expression difference between these two groups, at each cell type. Therefore, $\boldsymbol{X}$ and $\boldsymbol{A}$ each has dimension $T \times 2K$ and $T \times NK$. These matrices are employed in the linear mixed-effect regression to model the fixed effect $\boldsymbol{\beta}_g$ and the random effect $\boldsymbol{u}_g$. Additionally, $\boldsymbol{a}_{nk} = (\hat{\theta}_{E,n1k}, \hat{\theta}_{E,n2k}, \ldots \hat{\theta}_{E,nt_nk})'$ denotes the initial cell type abundance information.

If there is no covariate at all, the dimension of the design matrix for the fixed effect is T by K, where K is the total number of cell types. When adding more covariate(s), this type of design matrix offers flexibility for the inclusion of them at either "cell-type-level" or "bulk-level". The inclusion of "cell-type-level" effect, for each covariate, leads to an expansion of design matrix column by another K. The inclusion of "bulk -level" effect, for each covariate, leads to an expansion of design matrix column by one. For example, when an additional binary covariate (i.e. gender) needs to be considered and the investigator suspect it has impact on each cell type's expression, the number of columns in the design matrix will be expanded by another K (thus dimension of T by $3K$). If gender is believed to only impact at the "bulk-level" without effects at each cell type, the number of columns in the design matrix will be expanded by one (thus dimension of T by $2K + 1$). Similar rules would apply as well to the inclusions of continuous variables. Note that the interpretation of the coefficient(s) would depend on the way that covariate(s) are implemented.

$$\boldsymbol{X} =$$

$$\begin{bmatrix}
\hat{\theta}_{E,111} & \hat{\theta}_{E,112} & \cdots & \hat{\theta}_{E,11K} & z_1\hat{\theta}_{E,111} & z_1\hat{\theta}_{E,112} & \cdots & z_1\hat{\theta}_{E,11K} \\
\hat{\theta}_{E,121} & \hat{\theta}_{E,122} & \cdots & \hat{\theta}_{E,12K} & z_1\hat{\theta}_{E,121} & z_1\hat{\theta}_{E,112} & \cdots & z_1\hat{\theta}_{E,12K} \\
\vdots & & \ddots & & & & & \\
\hat{\theta}_{E,1t_11} & \hat{\theta}_{E,1t_12} & \cdots & \hat{\theta}_{E,1t_1K} & z_1\hat{\theta}_{E,1t_11} & z_1\hat{\theta}_{E,1t_12} & \cdots & z_1\hat{\theta}_{E,1t_1K} \\
\vdots & & & \ddots & & & & \\
\hat{\theta}_{E,N11} & \hat{\theta}_{E,N12} & \cdots & \hat{\theta}_{E,N1K} & z_N\hat{\theta}_{E,N11} & z_1\hat{\theta}_{E,N12} & \cdots & z_1\hat{\theta}_{E,N1K} \\
\hat{\theta}_{E,N21} & \hat{\theta}_{E,N22} & \cdots & \hat{\theta}_{E,N2K} & z_N\hat{\theta}_{E,N21} & z_1\hat{\theta}_{E,N22} & \cdots & z_1\hat{\theta}_{E,N2K} \\
\vdots & & & & \ddots & & & \\
\hat{\theta}_{E,Nt_N1} & \hat{\theta}_{E,Nt_N2} & \cdots & \hat{\theta}_{E,Nt_NK} & z_N\hat{\theta}_{E,Nt_N1} & z_N\hat{\theta}_{E,Nt_N2} & \cdots & z_1\hat{\theta}_{E,Nt_NK}
\end{bmatrix}$$

$\boldsymbol{A} =$

$$\begin{bmatrix} \boldsymbol{a}_{11} & \mathbf{0} & \mathbf{0} & \dots & \mathbf{0} & \boldsymbol{a}_{12} & \mathbf{0} & \mathbf{0} & \dots & \mathbf{0} & \boldsymbol{a}_{1K} & \mathbf{0} & \mathbf{0} & \dots & \mathbf{0} \\ \mathbf{0} & \boldsymbol{a}_{21} & \mathbf{0} & \dots & \mathbf{0} & \mathbf{0} & \boldsymbol{a}_{22} & \mathbf{0} & \dots & \mathbf{0} & \mathbf{0} & \boldsymbol{a}_{2K} & \mathbf{0} & \dots & \mathbf{0} \\ \vdots & \ddots & & & \vdots & & \ddots & & & & \vdots & \ddots & & & \vdots \\ \vdots & & & \ddots & \vdots & & & & \ddots & & \vdots & & & \ddots & \vdots \\ \mathbf{0} & \mathbf{0} & \mathbf{0} & \dots & \boldsymbol{a}_{N1} & \mathbf{0} & \mathbf{0} & \mathbf{0} & \dots & \boldsymbol{a}_{N2} & \mathbf{0} & \mathbf{0} & \mathbf{0} & \dots & \boldsymbol{a}_{NK} \end{bmatrix}$$

1.3 PDBP and TEDDY Consortia Overview

TEDDY

The TEDDY study recruited and followed infants at high risk for Type 1 Diabetes (T1D) based on Human Leukocyte Antigen (HLA) genotypes across six clinical centers in four countries: the U.S., Finland, Germany, and Sweden. In total, 8,676 infants were monitored from birth through infancy at three-month intervals for blood sampling and islet autoantibody (IAb) assessments, and then at intervals of three to six months based on their IAb status up to the age of 15 or until a T1D diagnosis was confirmed. This longitudinal cohort began exhibiting islet autoimmunity (IA), as indicated by consistent IAb presence, starting from 9 months of age, with a significant occurrence observed between the first and second years. For RNA-seq transcriptome profiling, subjects who developed IA by May 31, 2012, and their corresponding controls were selected for RNA-seq transcriptome profiling, using the longitudinal whole blood samples before IAbs onset in each case-control pair.

Transcripts per million (TPM) were used for the RNA-seq analysis. Due to the high-proportion of red blood cells, the whole blood mRNA contains abundance of hemoglobin mRNA (hgbRNA), which could interfere with the counts of other genes (Hwang et al. [2020]), and therefore, twelve hgbRNA genes ($HBA1$, $HBA2$, HBB, $HBBP1$, HBD, $HBE1$, $HBG1$, $HBG2$, HBM, $HBQ1$, HBZ, and $HBZP1$) were removed before TPM normalization (Hwang et al. [2020]). Poor quality samples were first removed based on QC metrics from FastQC.

The TEDDY RNA-seq samples were processed using the Illumina TruSeq Stranded mRNA Sample Prep Kit, and sequencing was carried out on the Illumina HiSeq4000 system with paired-end 2 x 101 bp reads, targeting 50 million reads per sample by the Broad Institute, Cambridge, MA. The alignment for the raw sequences was performed against the Gencode Genome Reference Consortium Build 38 (GRCh38.p12) Release 31 reference genome and comprehensive gene annotation. Sequencing reads were aligned using the STAR aligner (Dobin et al. [2013]) (version 2.6.1d) within the framework of the Trans-Omics for Precision Medicine (TOPMed) GTEx RNA-seq analysis pipeline. To ensure accuracy, ribosomal RNA (rRNA) sequences were detected and filtered out using the bbsplit tool (version 38.86) (`https://sourceforge.net/projects/bbmap/`) against the Ensembl rRNA database (Ensembl Archive Release 97). Following this, RNA-SeQC (version 2.1.0) (DeLuca et al. [2012]) was utilized to quantify gene expression levels. Quality control was further enforced by assessing RNA integrity through the qSVA scoring system (Jaffe et al. [2017]). Any samples that exhibited a qSVA score falling below three times the standard deviation from the mean were excluded from subsequent analysis.

PDBP

PD RNA was extracted and prepared from BioFIND, PDBP, and PPMI cohorts samples, which are part of the AMP-PD study, and sequenced at a length of 150 base pairs (bp) at Hudson Alpha (https://www.hudsonalpha.org/). Detail RNA preparation and processing protocol can be found from https://amp-pd.org/transcriptomics-data. RNA count data were generated through STAR aligner (Dobin et al. [2013]), Samtools (Li et al. [2009]), and featureCounts (Liao et al. [2014]) after quality control.

2 Simulation Details

2.1 Real Data Selection and Cell-Specific Underlying Parameters' Estimation

The gene expression specific to each cell type is obtained from an actual study (GSE60424), which investigates differences in the transcriptome among six immune cell types (Neutrophils, Monocytes, B-cells, CD4 T cells, CD8 T cells, and Natural Killer cells) between healthy individuals and patients with immune-associated diseases (specifically, Type 1 Diabetes, Amyotrophic Lateral Sclerosis, Sepsis, and Multiple Sclerosis patients) as reported in (Linsley et al. [2014]). The underlying expression parameters for a given cell type k and gene g are estimated using the *estParam* function from the *PROPER package* (Wu et al. [2015]). This estimation assumes that the RNA-seq read counts follow a negative binomial distribution ($NB(\mu_{gk}, \phi_{gk})$), where μ_{gk} and ϕ_{gk} represent the mean expression and biological dispersion for gene g, respectively.

The initial parameters specific to cells and genes are initially refined using Human Genome-Wide annotations. Genes lacking corresponding genetic symbols from org.HS.eg.db (Carlson [2021]) are excluded, resulting in 30,080 remaining genes. Subsequently, these gene-specific underlying estimators are further filtered based on specific thresholds ($\hat{\mu}_{gk} > 1$ and $\hat{\phi}_{gk} > 0.001$), resulting in a total of 1,540 underlying gene estimations that serve as references for simulations. Tables S1 and S2 present the empirical mean and covariance matrix for the cell-specific filtered gene expression parameters.

	B-Cell	CD4	CD8	Monocytes	Neutrophils	NK
$\bar{\boldsymbol{\mu}}_m$	4.53	4.48	4.56	4.72	4.49	4.53
$\bar{\boldsymbol{\mu}}_\phi$	-4.04	-4.06	-3.60	-4.00	-3.26	-3.82

Table S1: The averages for underlying gene expression parameters of the 1,540 genes that remain for each cell type in healthy samples.

$\hat{\boldsymbol{\Sigma}}_m$ ($\hat{\boldsymbol{\Sigma}}_\phi$)	B-Cell	CD4	CD8	Monocytes	Neutrophils	NK
B-Cell	2.19 (2.00)	1.73 (0.95)	1.69 (0.75)	1.50 (0.61)	1.30 (0.55)	1.47 (0.71)
CD4		2.09 (2.02)	1.98 (1.02)	1.38 (0.63)	1.26 (0.60)	1.63 (0.78)
CD8			2.02 (1.90)	1.42 (0.64)	1.25 (0.57)	1.73 (0.80)
Monocytes				2.16 (1.72)	2.03 (0.74)	1.41 (0.57)
Neutrophils					4.05 (2.32)	1.44 (0.56)
NK						1.80 (2.01)

Table S2: Variances and covariances among the underlying gene expression parameters of the 1,540 retained genes across various cell types in healthy samples.

Celltype1-celltype6 will mask cell type names for further simulations. *Multivariate Gaussian* distributions are used to simulate two matrices representing cell-specific gene expression parameters for each subject. The underlying cell-specific gene expression matrix is generated through a re-parameterized *Gamma Distribution* for each sample, as depicted in the equations below.

$$\boldsymbol{M} \sim MVN(\bar{\boldsymbol{\mu}}_m, \hat{\boldsymbol{\Sigma}}_m)$$
$$\boldsymbol{\Phi} \sim MVN(\bar{\boldsymbol{\mu}}_\phi, \hat{\boldsymbol{\Sigma}}_\phi)$$
$$\boldsymbol{P} \sim \Gamma(\frac{1}{\exp(\boldsymbol{\Phi})}, \exp(\boldsymbol{M}) \times \exp(\boldsymbol{\Phi}))$$

2.2 Cell mixture proportion generation

Cell proportion estimations rely on 16 different single-cell RNA sequencing labeling studies, each with varying numbers of cell types ranging from 3 to 13. Using bootstrapping and resampling techniques, we created a pooled sample of 80 cells, allowing us to empirically derive cell-type compositions from this cell-label pool. This procedure was repeated 200 times, resulting in a 200×6 cell composition matrix. The matrix was solved using the *Dirichlet Distribution* via the *dirichlet.mle* function from the *sirt* package (Robitzsch [2022]). This yielded a vector of cell proportion distributional parameters for control samples, denoted as $\boldsymbol{\alpha}_{ctrl} = [8.85, 6.49, 5.98, 5.28, 4.22, 3.85]$. For the case samples, the *Dirichlet* parameters were generated by redistributing the sum of α values ($\alpha_{Total} = \sum_{k=1}^{6} \alpha_{ctrl}$). The cell proportion distributional parameters for cases are: $\boldsymbol{\alpha}_{case} = [1.90, 2.25, 2.10, 5.72, 7.33, 15.37]$.

3 Evaluation Metric

Lin's concordance correlation coefficient was designed for univariate outcome but not specifically for high-dimension compositional measurements, where the relative abundances within a subject could be dependent and subject to the positiveness and unit-sum constraints. To address these aspects, Cui et al. (Cui et al. [2021]) propose a general formulation of CCC by substituting the expected squared difference to Euclidean distance (shown in the main text) or Aitchison distance (Aitchison et al. [2000]), which is a popular distance measure for paired compositional data, defined below:

$$d_A^2(\boldsymbol{\Theta}, \hat{\boldsymbol{\Theta}}) = \sum_{k=1}^{K} \left[\log \left(\frac{\hat{\boldsymbol{\Theta}}^{(k)}}{h(\hat{\boldsymbol{\Theta}})} \right) - \log \left(\frac{\boldsymbol{\Theta}^{(k)}}{h(\boldsymbol{\Theta})} \right) \right]^2$$

where $h(\boldsymbol{\Theta}) = \left(\prod_{l=1}^{K} \boldsymbol{\Theta}^{(l)} \right)^{\frac{1}{K}}$ indicates the geometric mean of the elements of $\boldsymbol{\Theta}$. If the proposed method leads to increased concordance, we can expect to observe positive differences in Lin's CCC and its adapted versions when comparing the improved cell proportion estimation with the initial estimation.

4 Simulation Results

Additional simulation results are presented here in the supplementary section, including six evaluation metrics: absolute bias difference (ABD)/ cell-type-specific ABD (Section 4.1), relative absolute bias difference ($rABD\%$)/ cell-type-specific $rABD\%$ (Section 4.2), correlation difference (CD, Section 4.3), differences of Lin's CCC ($\Delta\rho_{\mathrm{C}}$, Section 4.4), differences of Euclidean-based CCC ($\Delta\rho_{\mathrm{C,E}}$, Section 4.5), and differences of Aitchison-based CCC ($\Delta\rho_{\mathrm{C,A}}$, Section 4.6) across different scenarios. In these scenarios, subject-specific variation (SSV) is set at levels of 0-5% and 10-20%. The effect sizes and sample sizes per group are respectively reflected by: $LFC = 0$, 0.5, 1.5, and $N = 25$, 75, 100. Each plot shows a particular sample size with various combinations of LFC and SSV.

For both ABD and $rABD\%$, if they are less than zero, it signifies that the evaluated method decrease the estimation bias. Furthermore, lower values additionally suggest better models' performances. If the evaluated method improve estimation concordance, we would anticipate observing positive values in $\Delta\rho_{\mathrm{C}}$, $\Delta\rho_{\mathrm{C,E}}$ and $\Delta\rho_{\mathrm{C,A}}$.

4.1 Absolute Bias Difference (ABD)

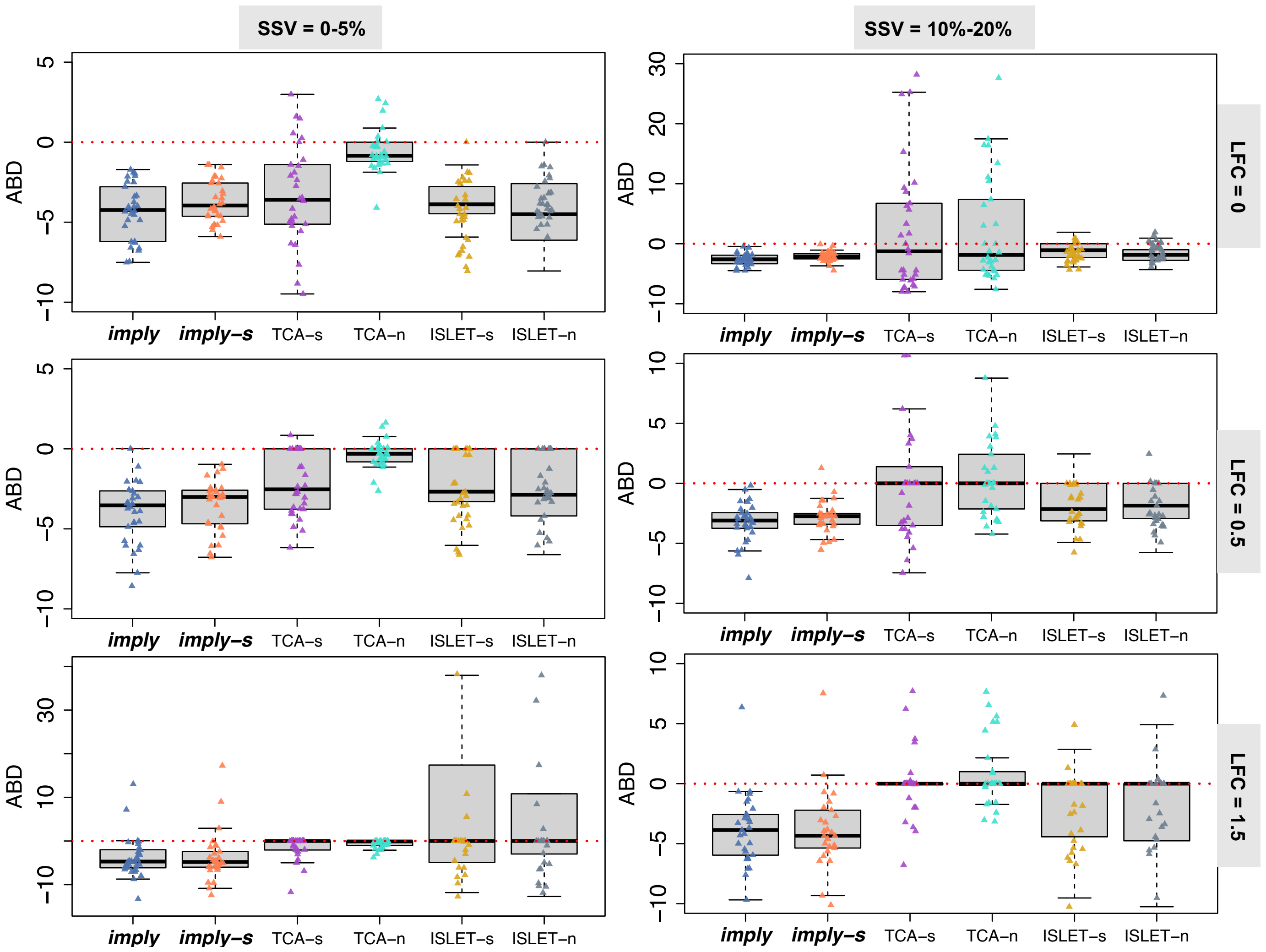

Figure S1: The absolute bias difference (ABD) is examined across various levels of Subject-Specific Variations (SSVs) represented in columns and effect sizes depicted in rows, focusing specifically on a sample size (N) of 25.

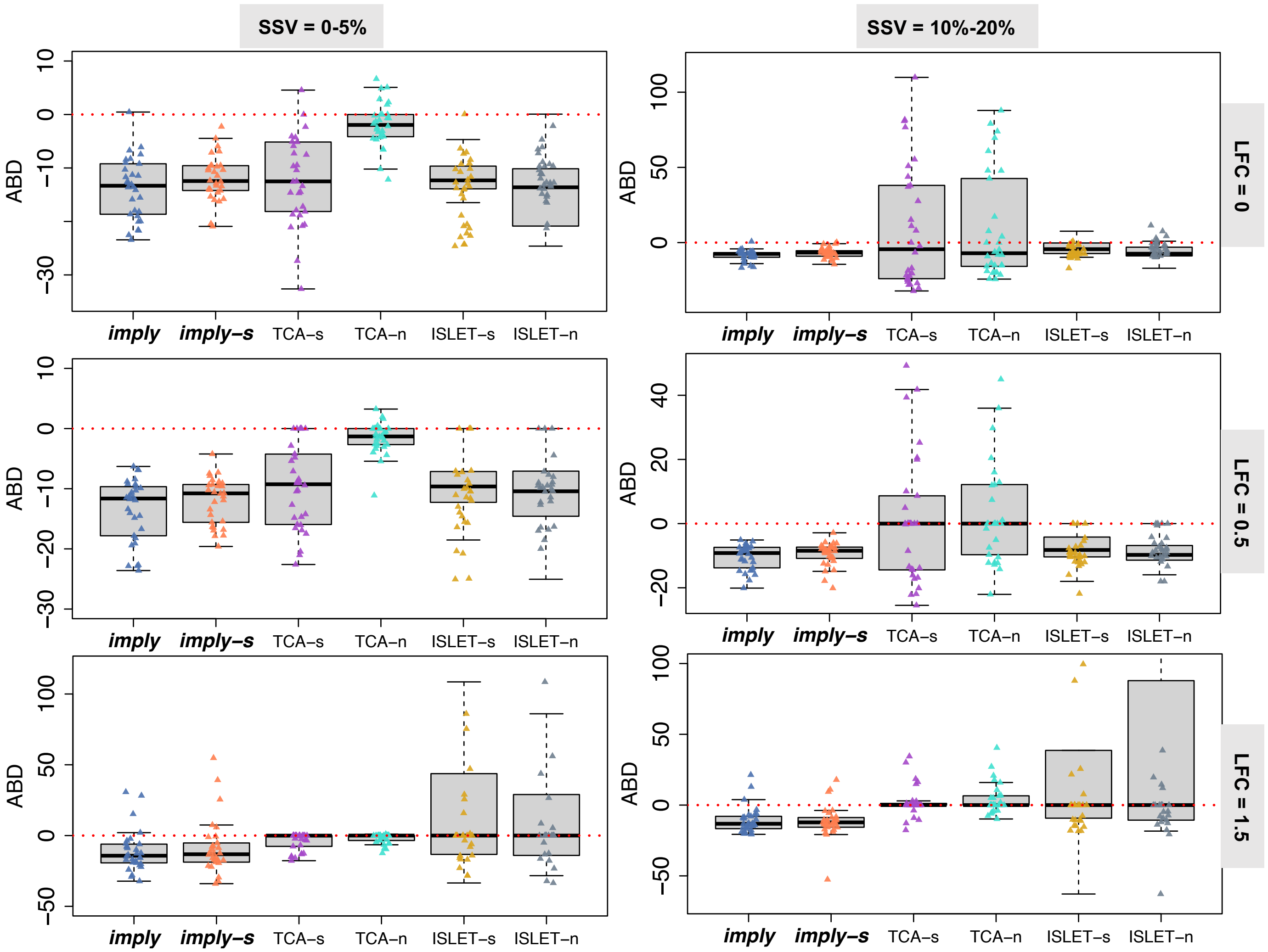

Figure S2: The absolute bias difference (ABD) is examined across various levels of Subject-Specific Variations (SSVs) represented in columns and effect sizes depicted in rows, focusing specifically on a sample size (N) of 75.

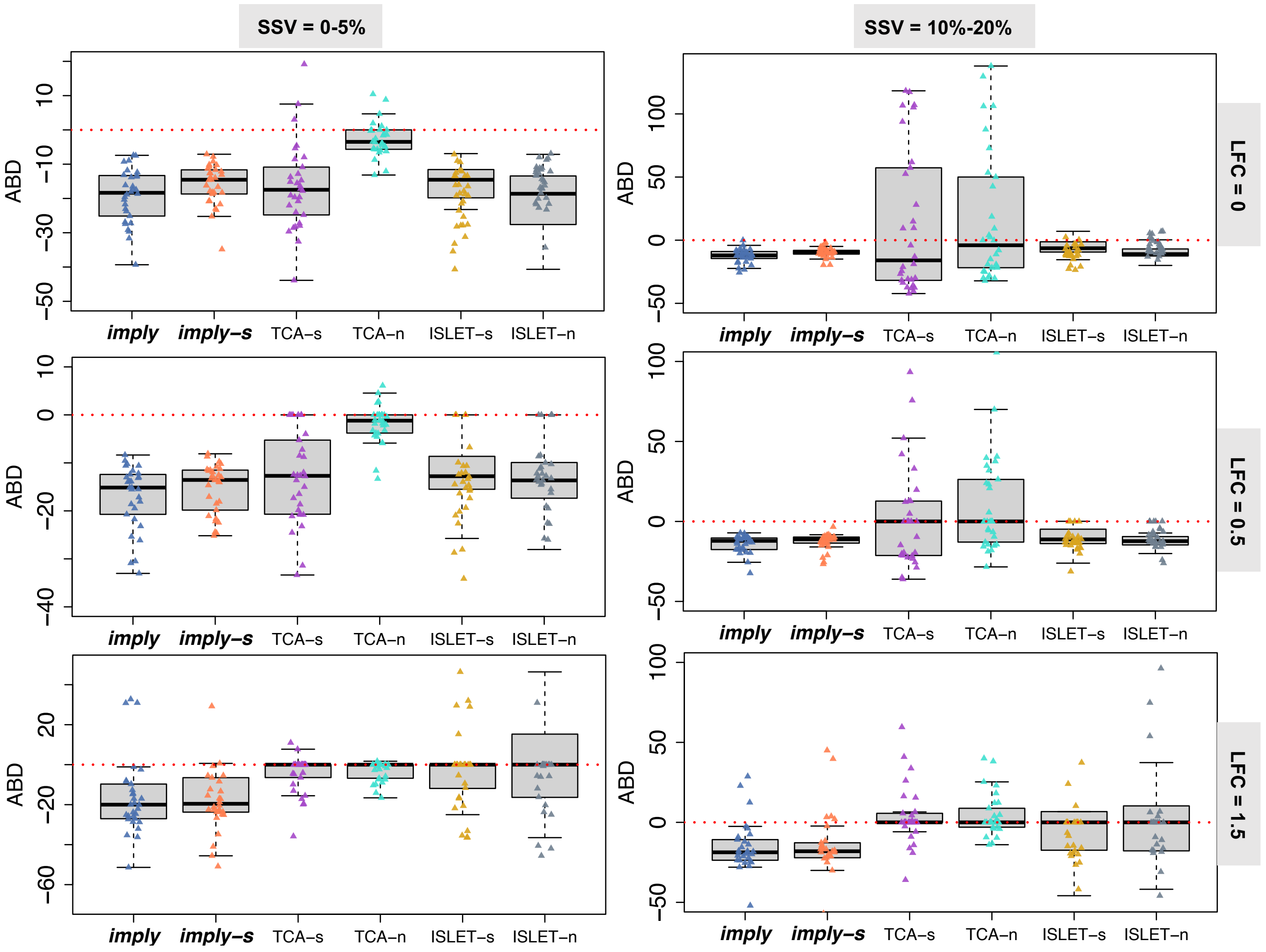

Figure S3: The absolute bias difference (ABD) is examined across various levels of Subject-Specific Variations (SSVs) represented in columns and effect sizes depicted in rows, focusing specifically on a sample size (N) of 100.

4.1.1 Cell-type-specific ABD

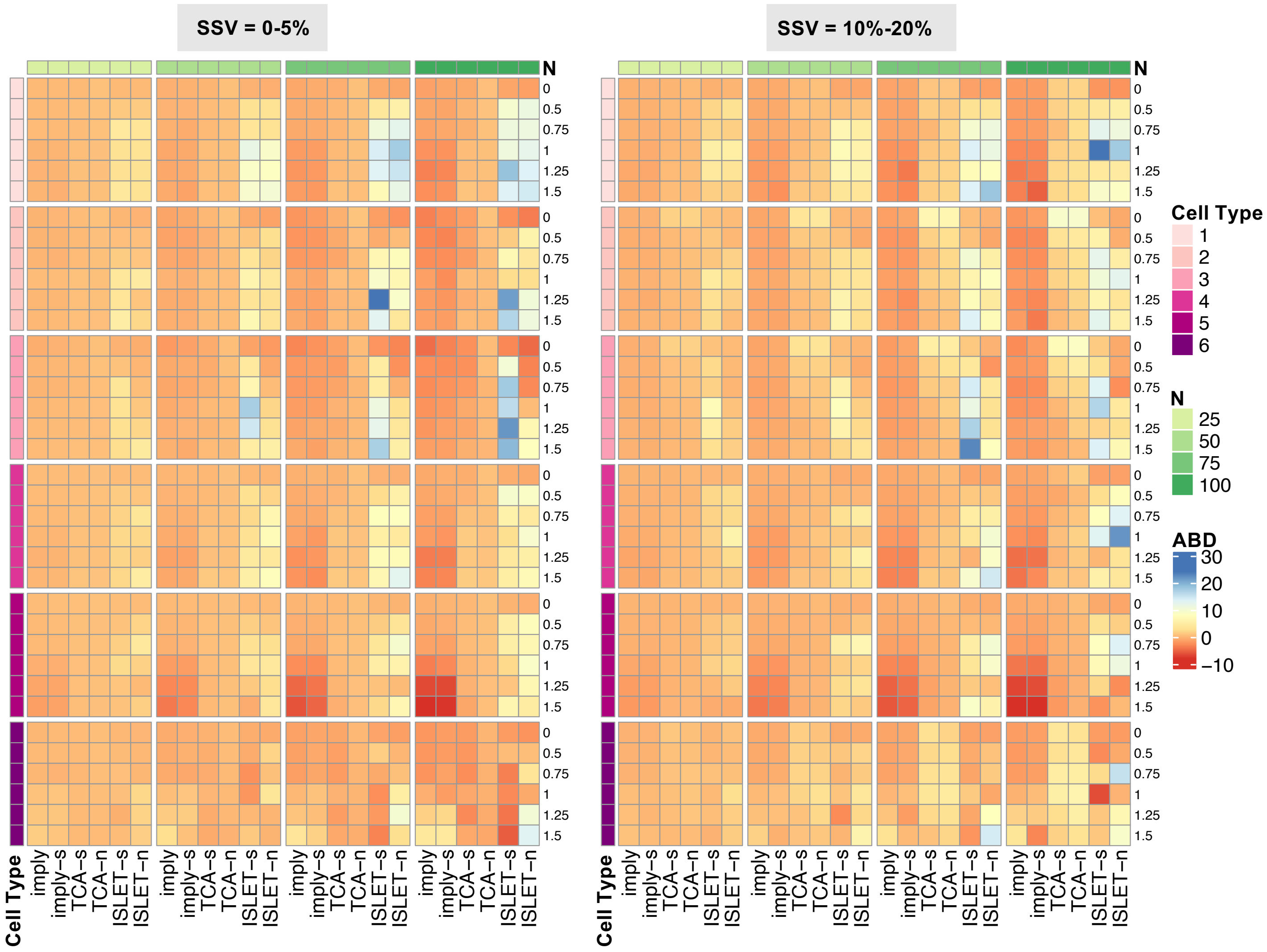

Figure S4: This heatmap displays the cell-type-specific absolute bias difference (ABD) evaluated across different Subject-Specific Variation (SSV) levels. The SSV levels are segregated into distinct panels. Within each panel, numbers on the right side of the heatmap indicate effect sizes. The legend at the top corresponds to the sample size (N), ranging from $N = 25$ to $N = 100$. On the left side of the heatmap, colors represent the six distinct cell types. The color intensity within the heatmap cells corresponds to the magnitude of ABD, with the scale provided on the right.

4.2 Relative absolute bias difference ($rABD\%$)

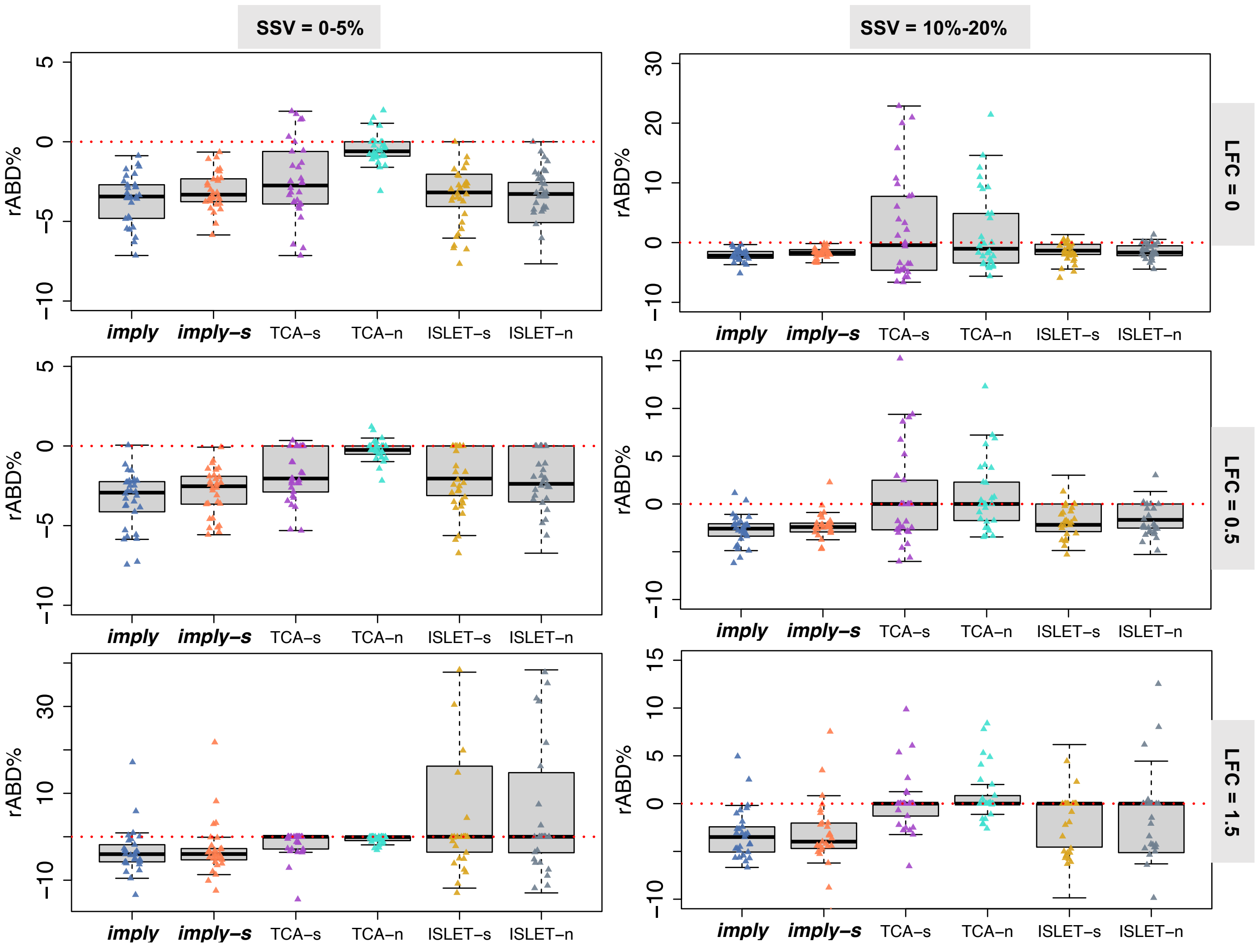

Figure S5: The relative absolute bias difference ($rABD\%$) is examined across various levels of Subject-Specific Variations (SSVs) represented in columns and effect sizes depicted in rows, focusing specifically on a sample size (N) of 25.

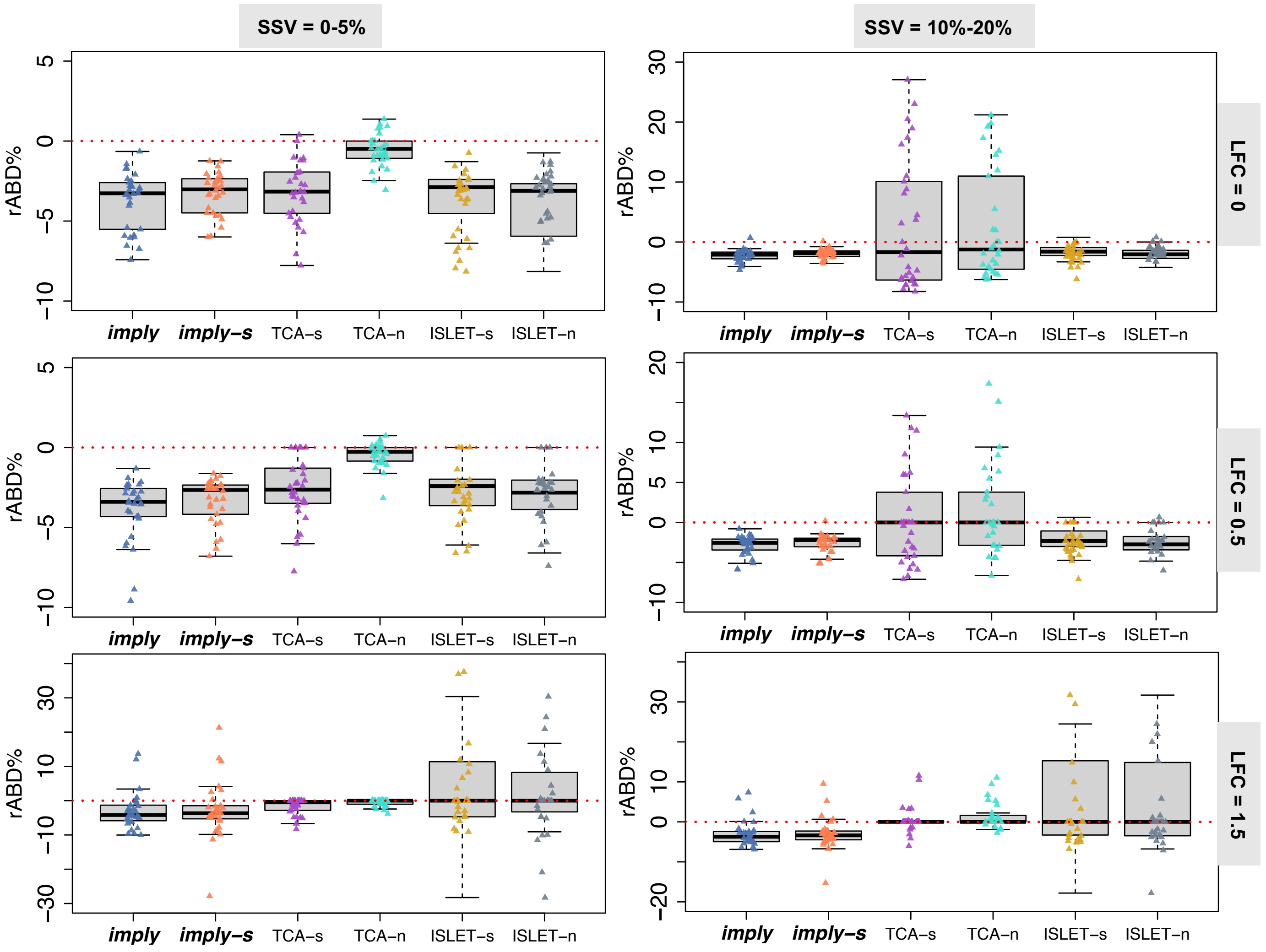

Figure S6: The relative absolute bias difference ($rABD\%$) is examined across various levels of Subject-Specific Variations ($SSVs$) represented in columns and effect sizes depicted in rows, focusing specifically on a sample size (N) of 75.

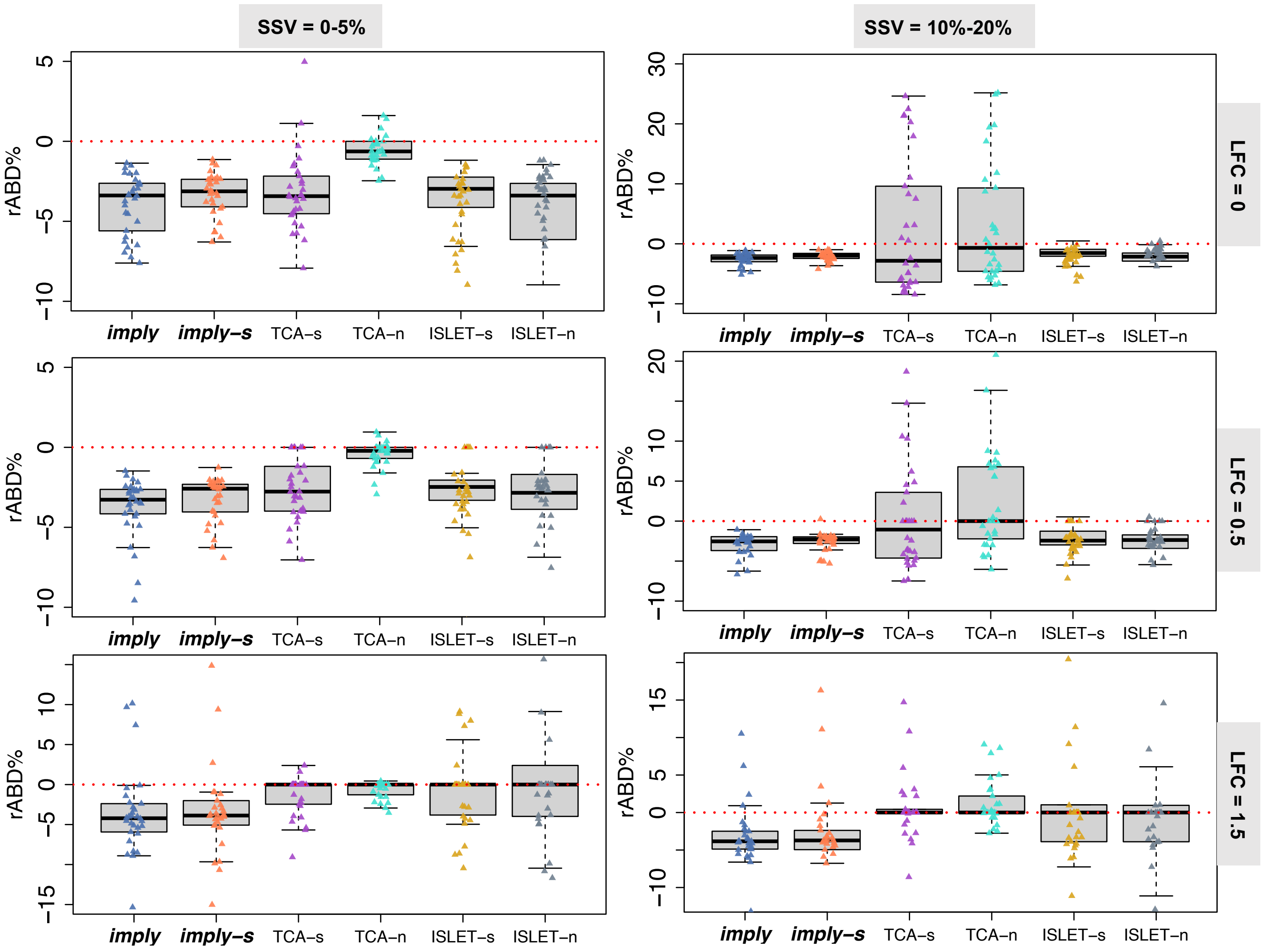

Figure S7: The relative absolute bias difference ($rABD\%$) is examined across various levels of Subject-Specific Variations (SSVs) represented in columns and effect sizes depicted in rows, focusing specifically on a sample size (N) of 100.

4.2.1 Cell-type-specific $rABD\%$

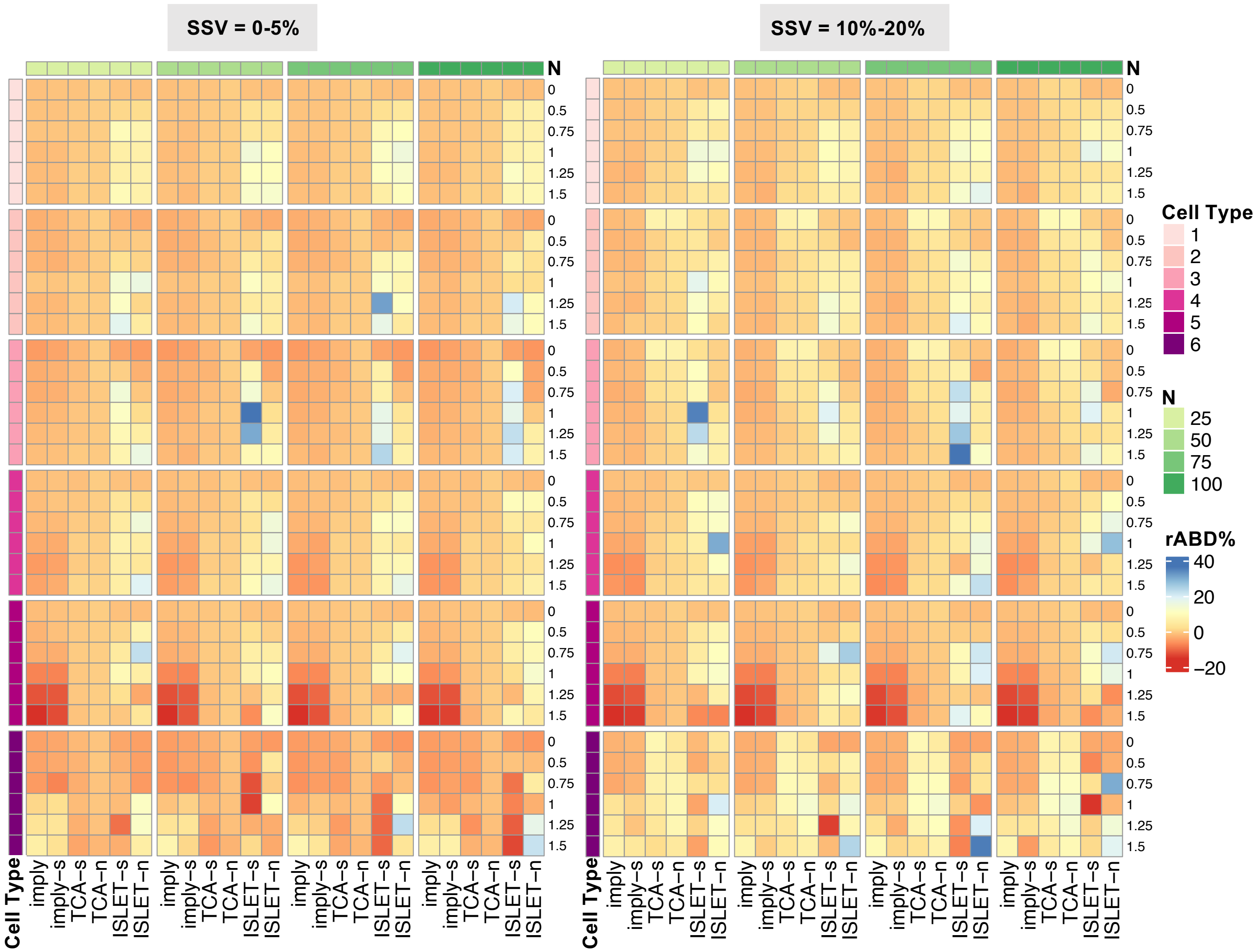

Figure S8: This heatmap displays the cell-type-specific relative absolute bias difference ($rABD\%$) evaluated across different Subject-Specific Variation (SSV) levels. The SSV levels are segregated into distinct panels. Within each panel, numbers on the right side of the heatmap indicate effect sizes. The legend at the top corresponds to the sample size (N), ranging from $N = 25$ to $N = 100$. On the left side of the heatmap, colors represent the six distinct cell types. The color intensity within the heatmap cells corresponds to the magnitude of $rABD\%$, with the scale provided on the right.

4.3 Correlation Differences (CD)

When $CD > 0$, it implies that the evaluated method enhances the correlation between estimated cell type proportions and the ground truth. A greater value indicates a more promising performance.

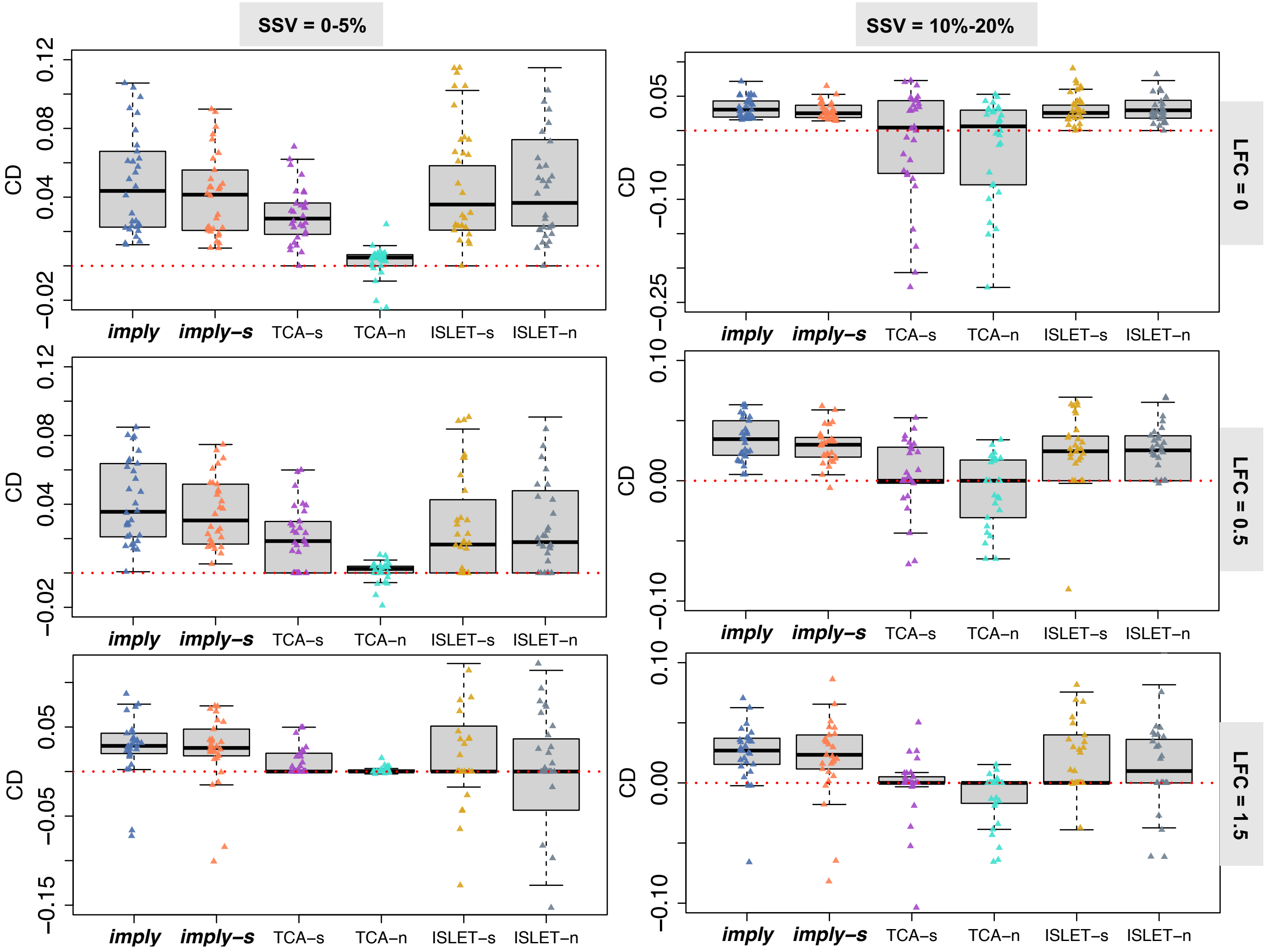

Figure S9: The correlation difference (CD) is examined across various levels of Subject-Specific Variations (SSVs) represented in columns and effect sizes depicted in rows, focusing specifically on a sample size (N) of 25.

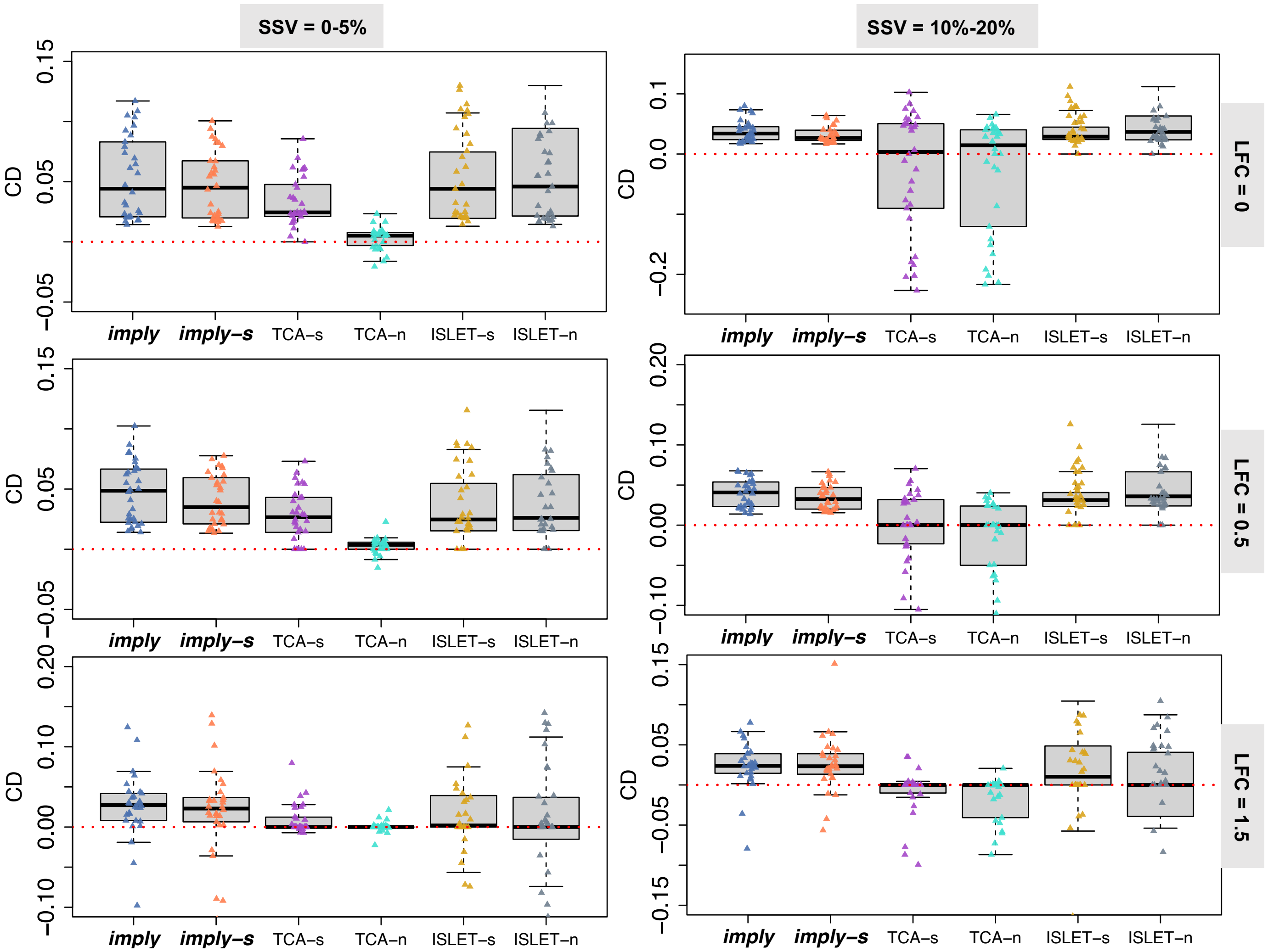

Figure S10: The correlation difference (CD) is examined across various levels of Subject-Specific Variations (SSVs) represented in columns and effect sizes depicted in rows, focusing specifically on a sample size (N) of 75.

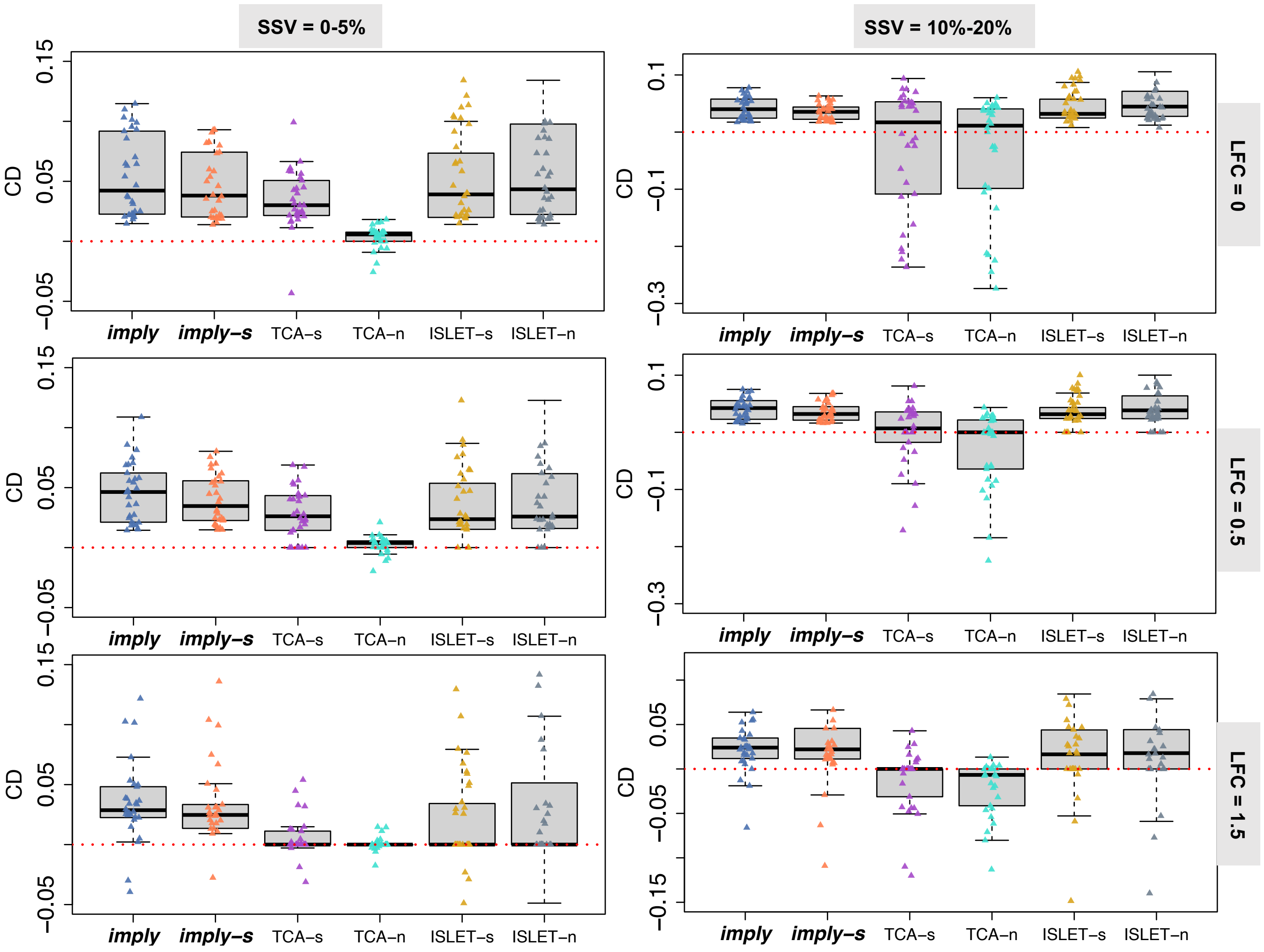

Figure S11: The correlation difference (CD) is examined across various levels of Subject-Specific Variations (SSVs) represented in columns and effect sizes depicted in rows, focusing specifically on a sample size (N) of 100.

4.4 Lin's CCC ($\Delta\rho_C$)

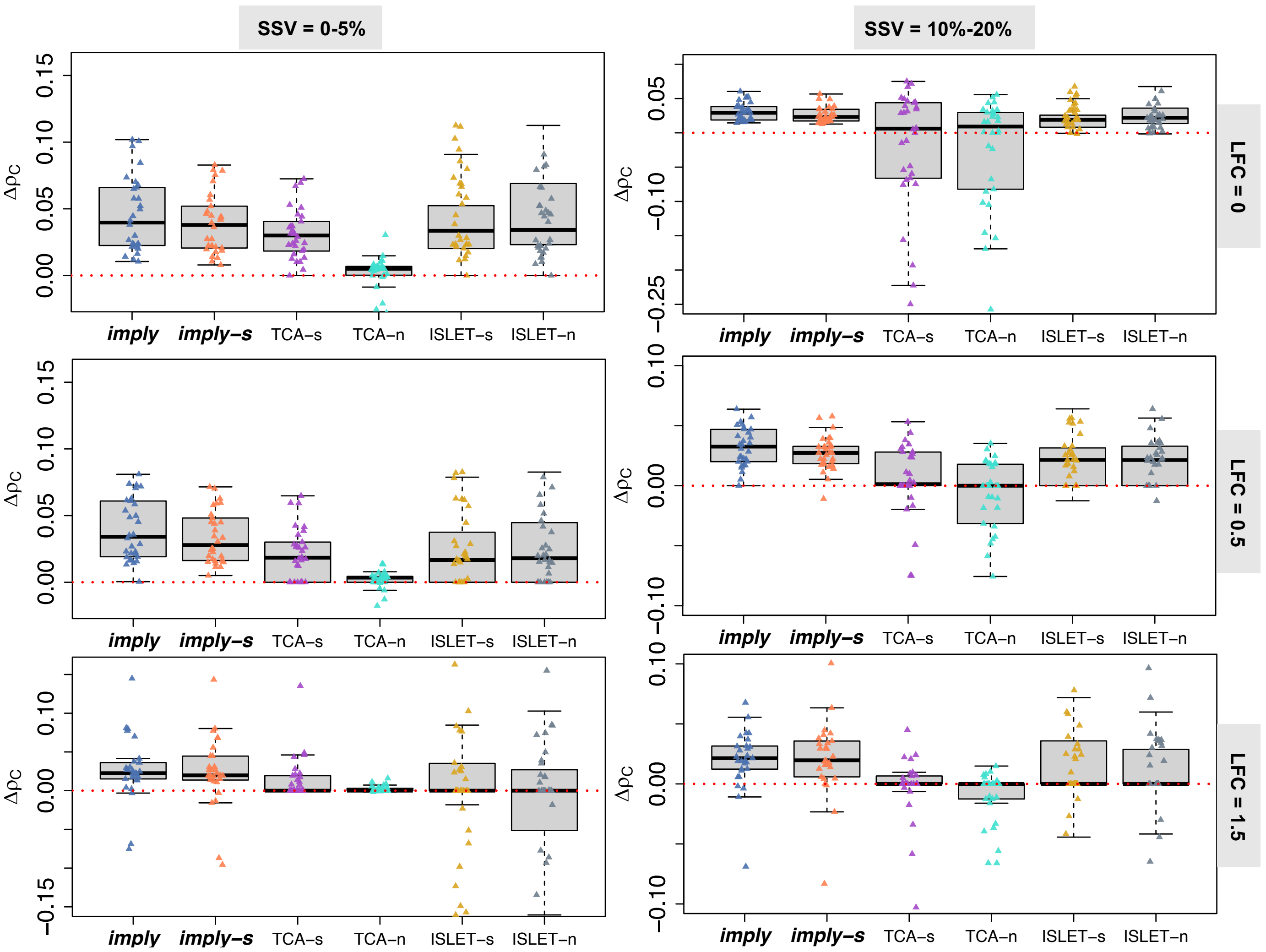

Figure S12: The difference of Lin's CCC ($\Delta\rho_C$) is examined across various levels of Subject-Specific Variations (SSVs) represented in columns and effect sizes depicted in rows, focusing specifically on a sample size (N) of 25.

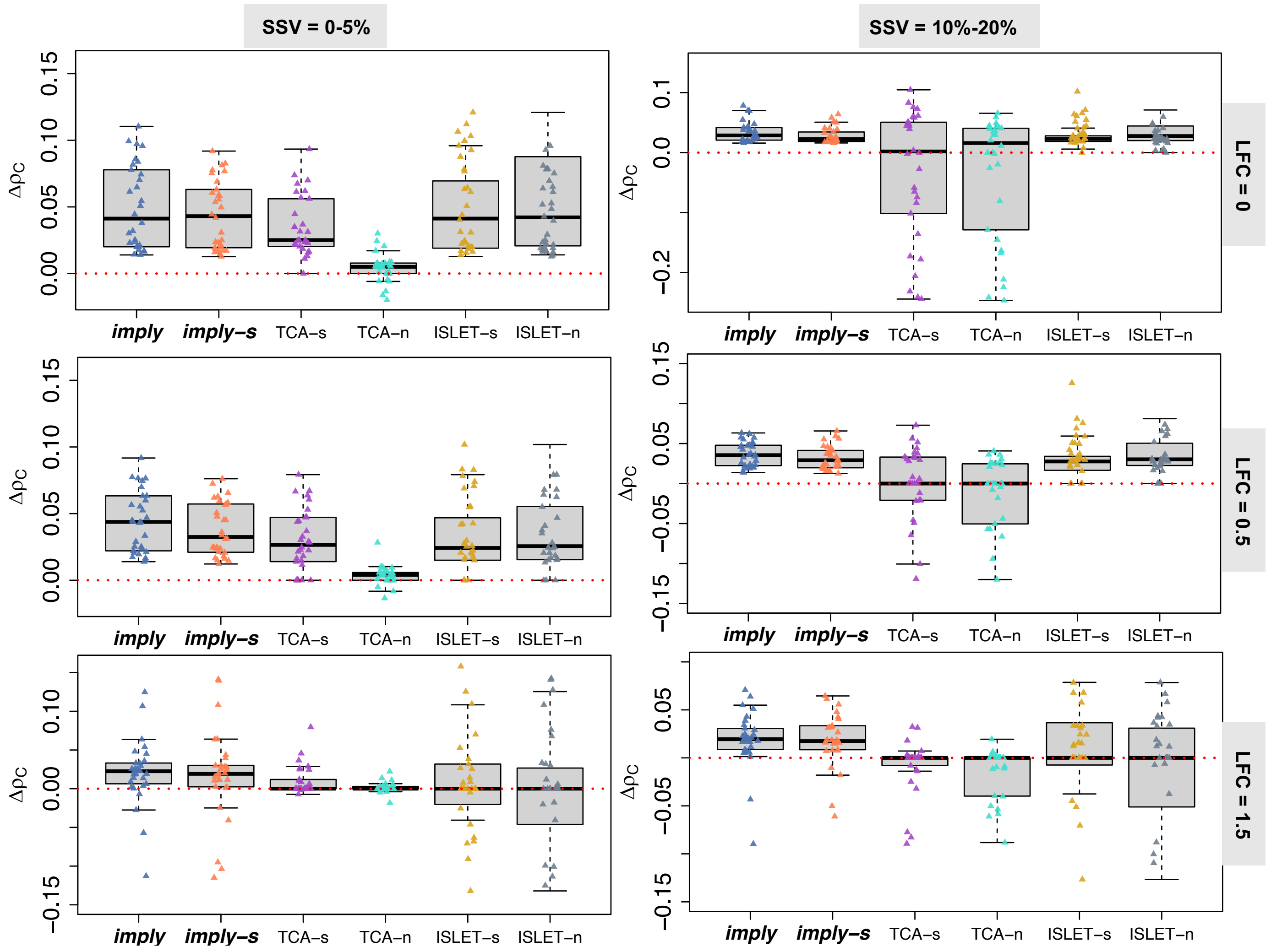

Figure S13: The difference of Lin's CCC ($\Delta\rho_C$) is examined across various levels of Subject-Specific Variations (SSVs) represented in columns and effect sizes depicted in rows, focusing specifically on a sample size (N) of 75.

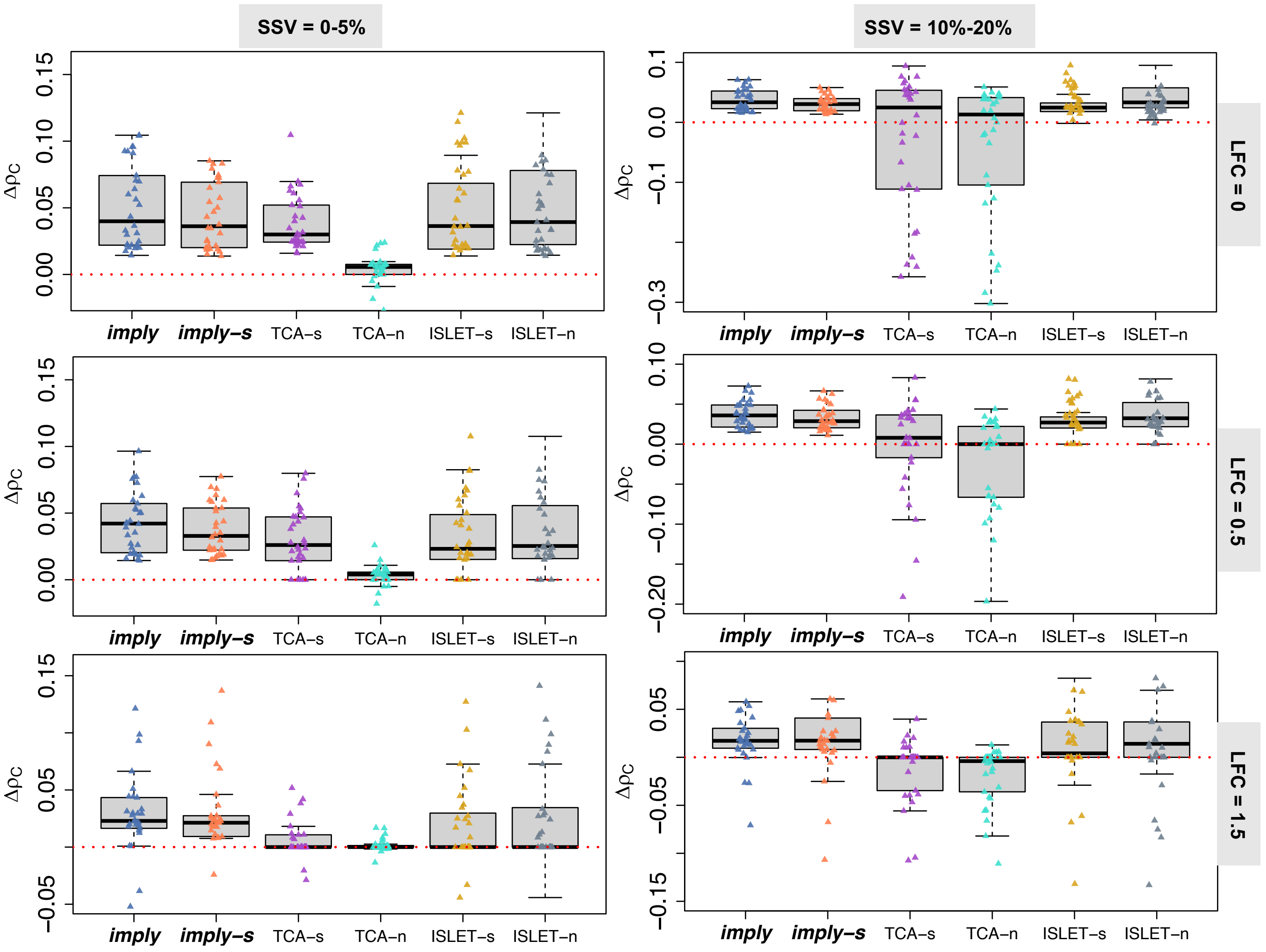

Figure S14: The difference of Lin's CCC ($\Delta\rho_C$) is examined across various levels of Subject-Specific Variations (SSVs) represented in columns and effect sizes depicted in rows, focusing specifically on a sample size (N) of 100.

4.5 Euclidean Based-CCC ($\Delta\rho_{C,E}$)

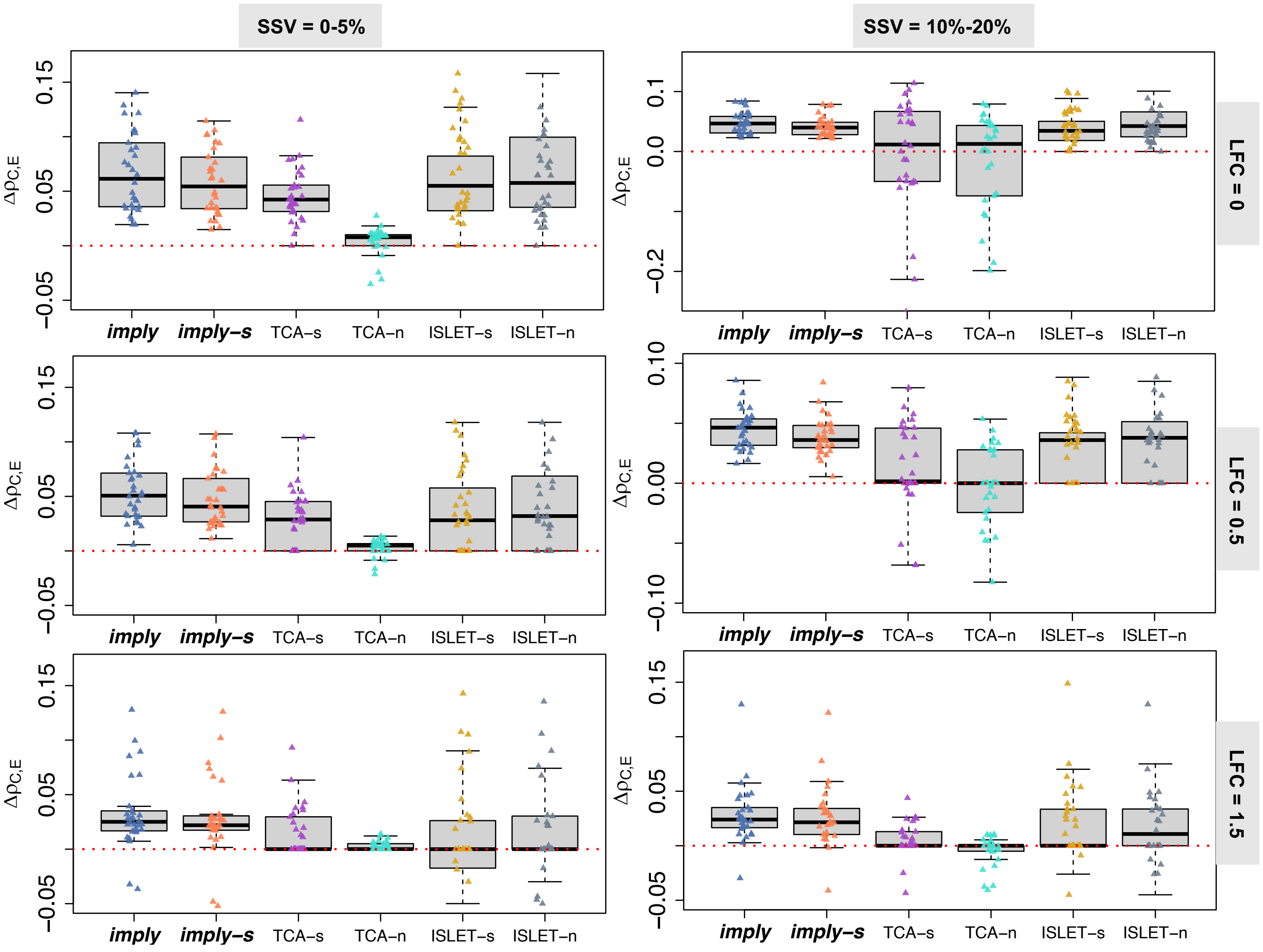

Figure S15: The difference of Euclidean-based CCC ($\Delta\rho_{C,E}$) is examined across various levels of Subject-Specific Variations (SSVs) represented in columns and effect sizes depicted in rows, focusing specifically on a sample size (N) of 25.

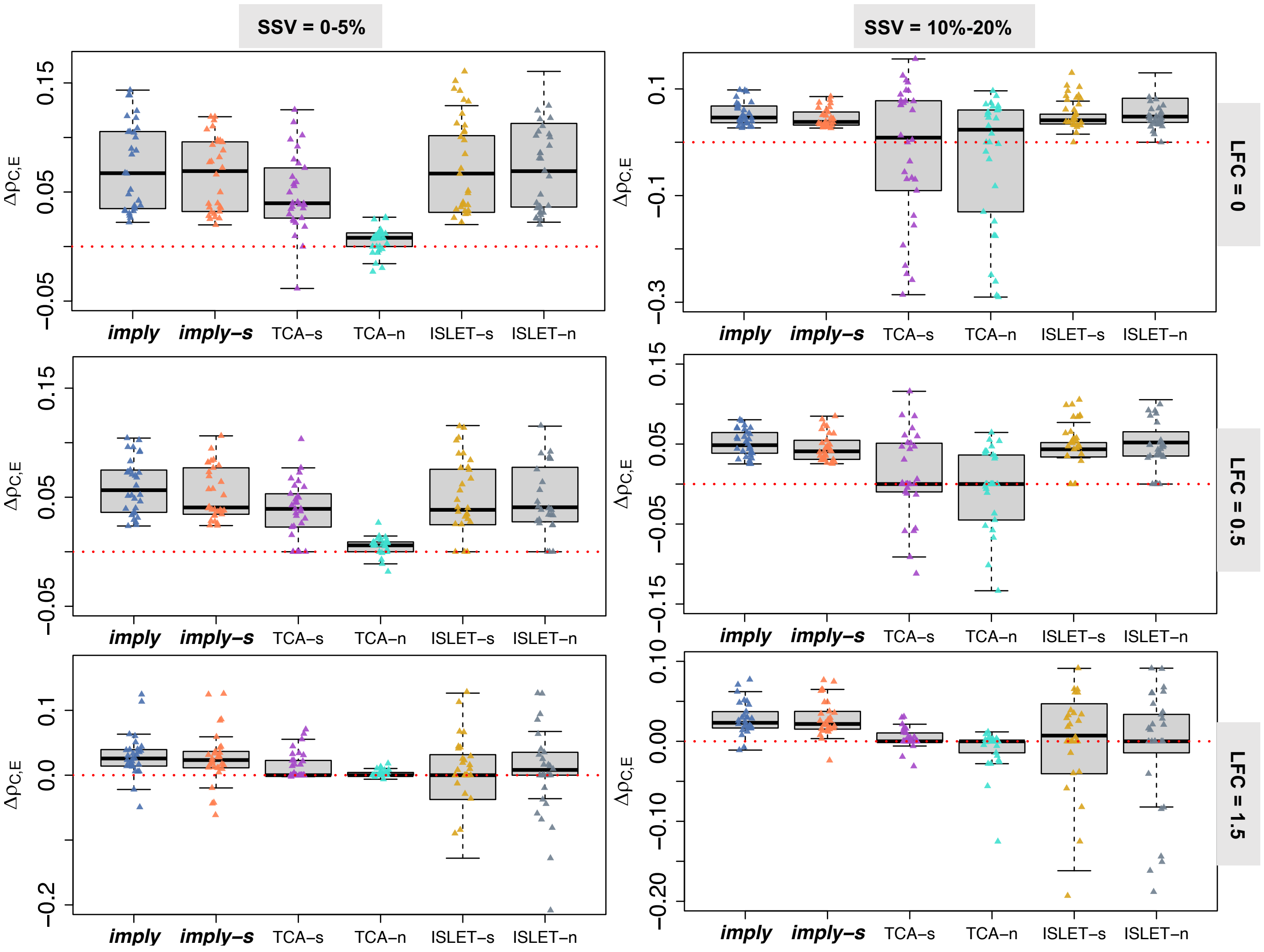

Figure S16: The difference of Euclidean-based CCC ($\Delta\rho_{\mathrm{C,E}}$) is examined across various levels of Subject-Specific Variations (SSVs) represented in columns and effect sizes depicted in rows, focusing specifically on a sample size (N) of 75.

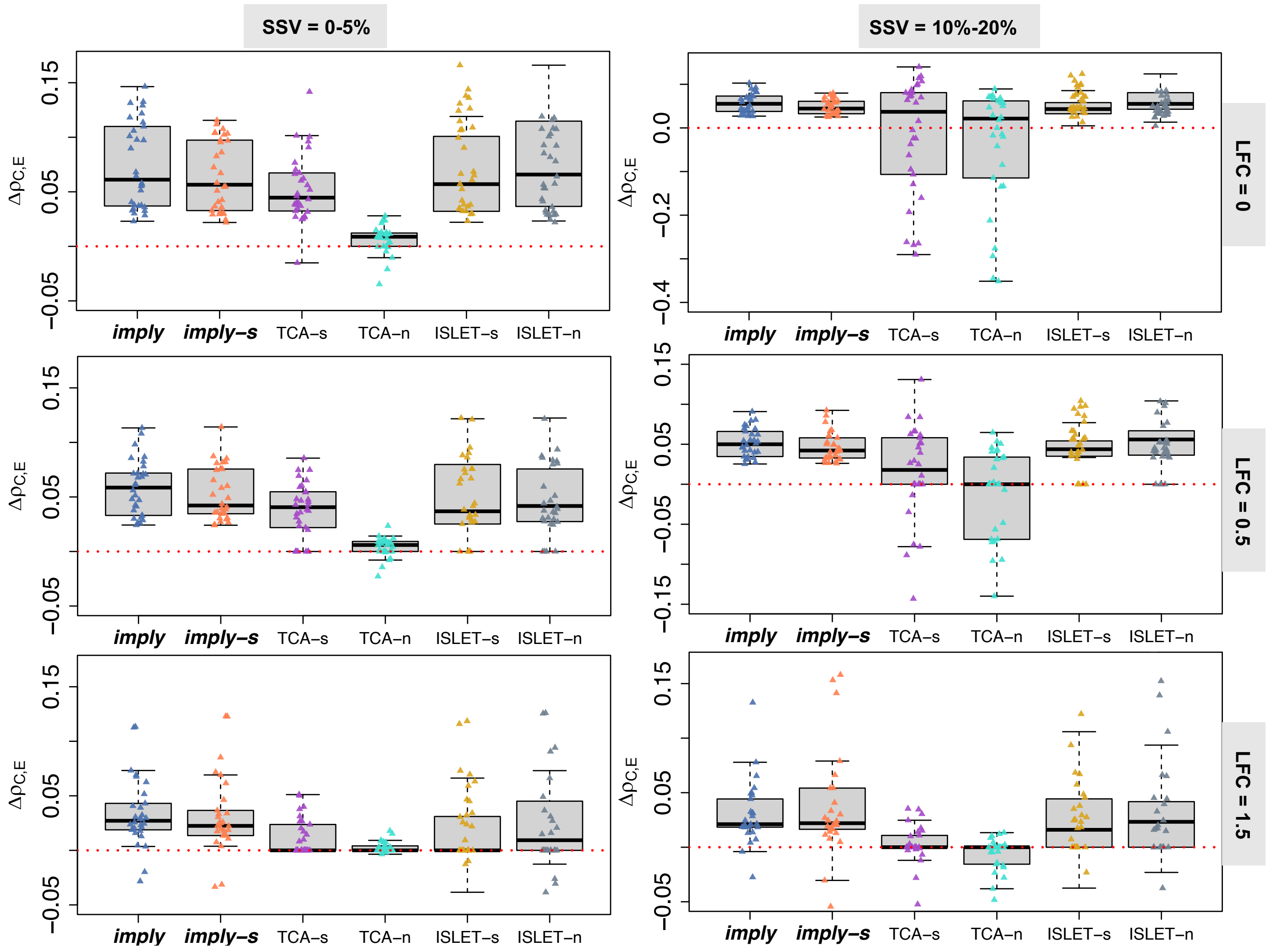

Figure S17: The difference of Euclidean-based CCC ($\Delta\rho_{\mathrm{C,E}}$) is examined across various levels of Subject-Specific Variations (SSVs) represented in columns and effect sizes depicted in rows, focusing specifically on a sample size (N) of 100.

4.6 Aitchison Based-CCC ($\Delta\rho_{C,A}$)

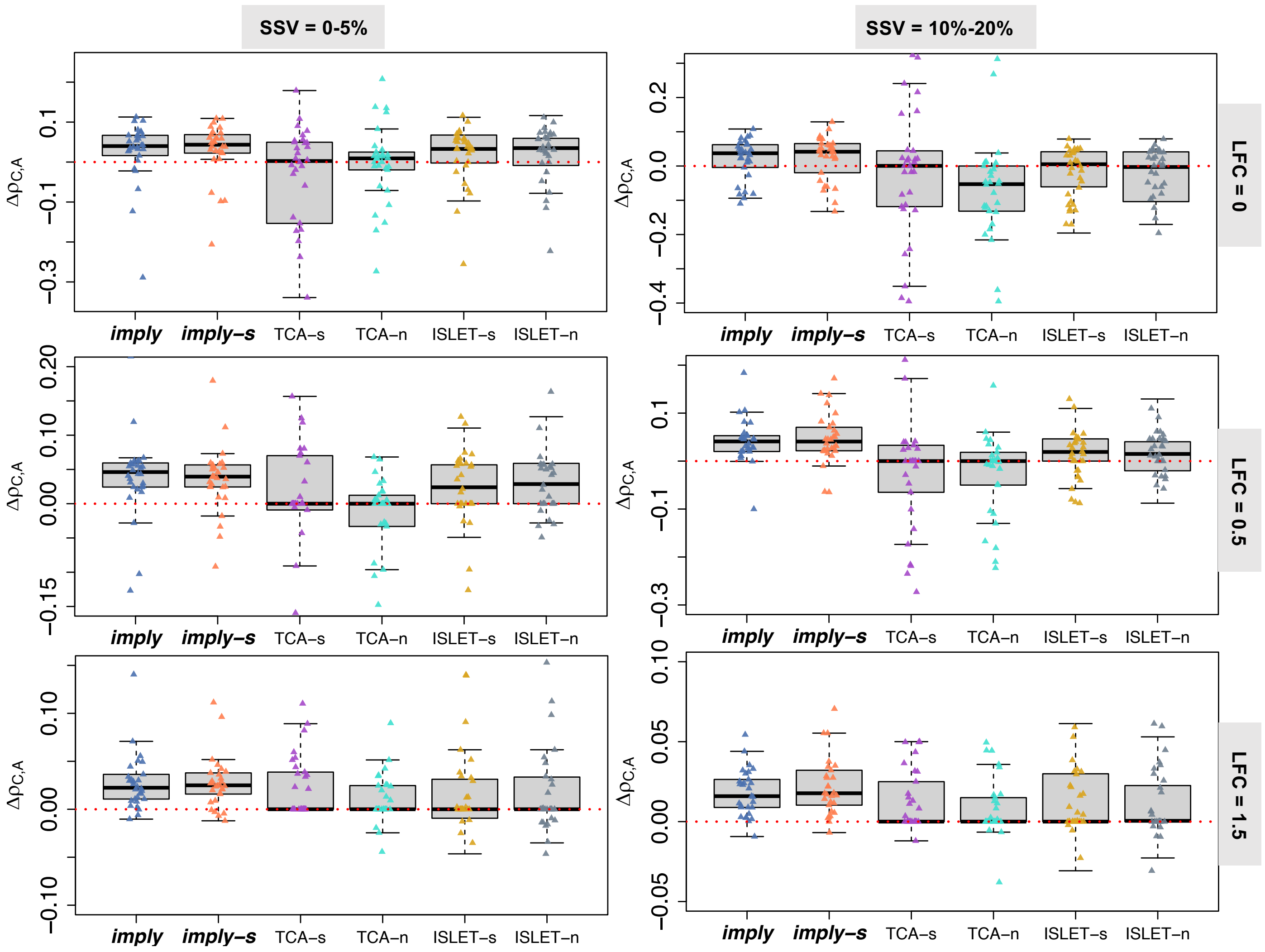

Figure S18: The difference of Aitchison-based CCC ($\Delta\rho_{C,A}$) is examined across various levels of Subject-Specific Variations (SSVs) represented in columns and effect sizes depicted in rows, focusing specifically on a sample size (N) of 25.

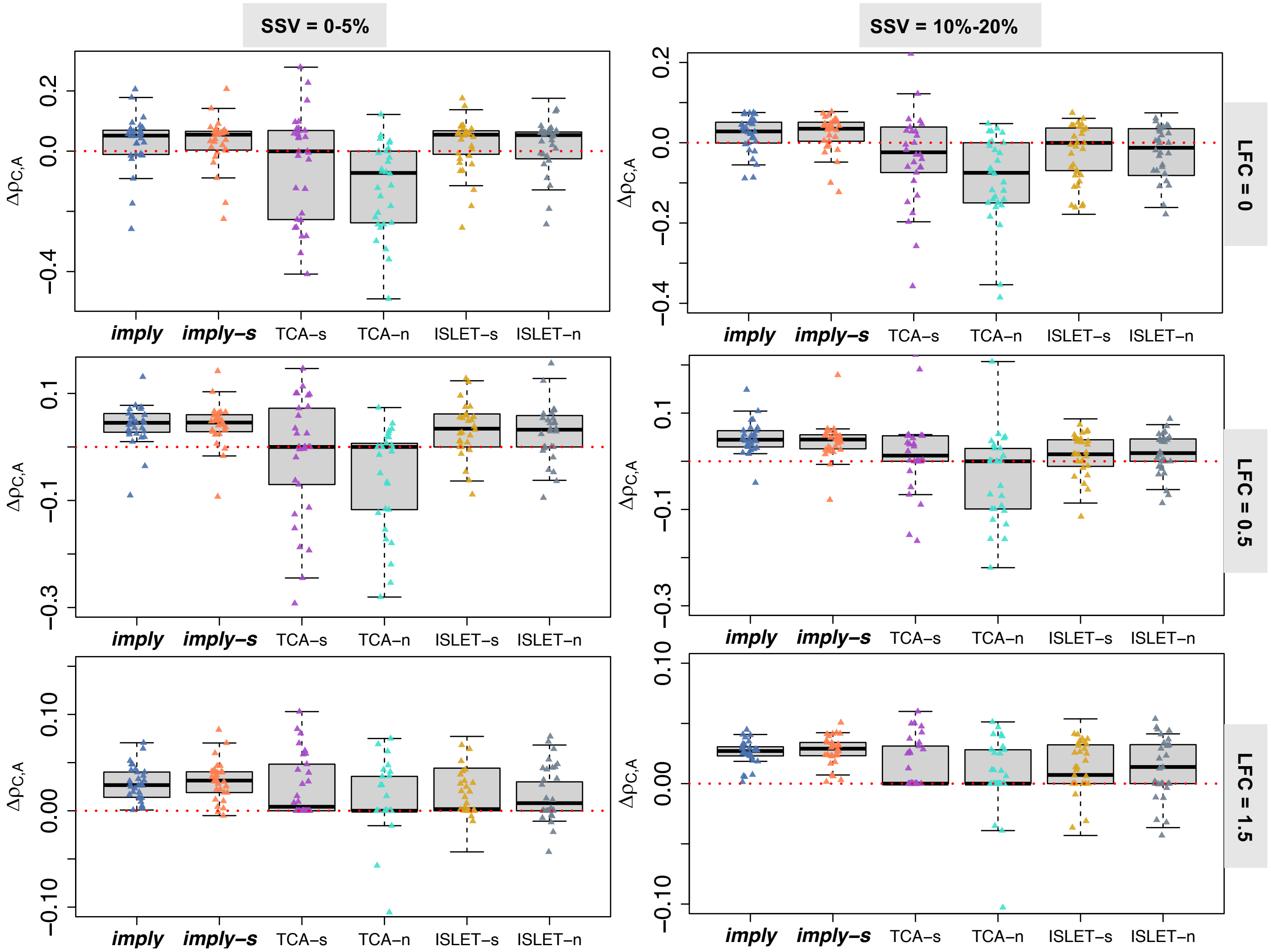

Figure S19: The difference of Aitchison-based CCC ($\Delta\rho_{\mathrm{C,A}}$) is examined across various levels of Subject-Specific Variations (SSVs) represented in columns and effect sizes depicted in rows, focusing specifically on a sample size (N) of 75.

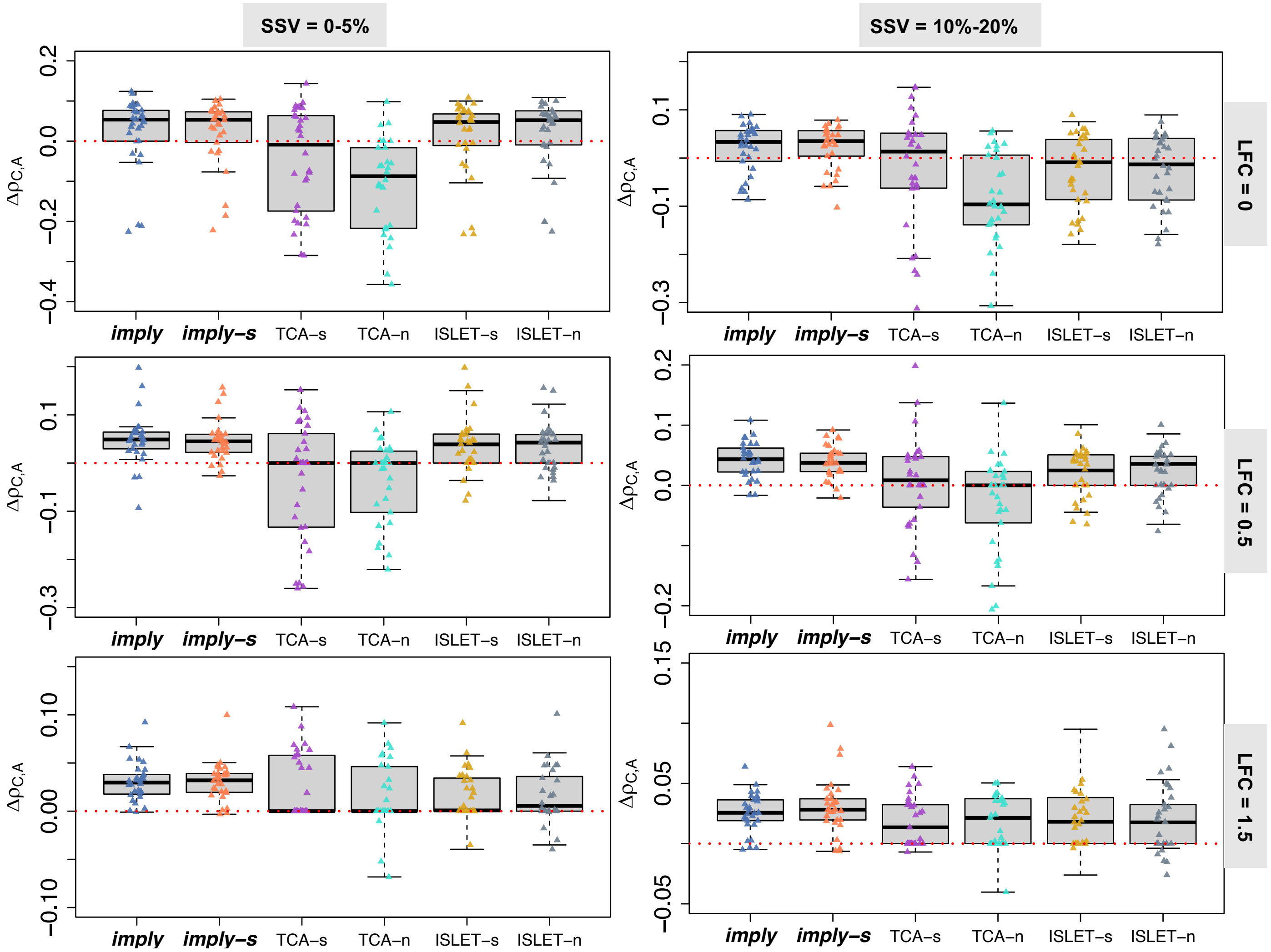

Figure S20: The difference of Aitchison-based CCC ($\Delta\rho_{\mathrm{C,A}}$) is examined across various levels of Subject-Specific Variations (SSVs) represented in columns and effect sizes depicted in rows, focusing specifically on a sample size (N) of 100.

5 Real Data Analysis

5.1 PDBP: Parkinson's Disease Biomarker Program

Here, we investigate the correlation between different cell type proportions and clinical indicators.

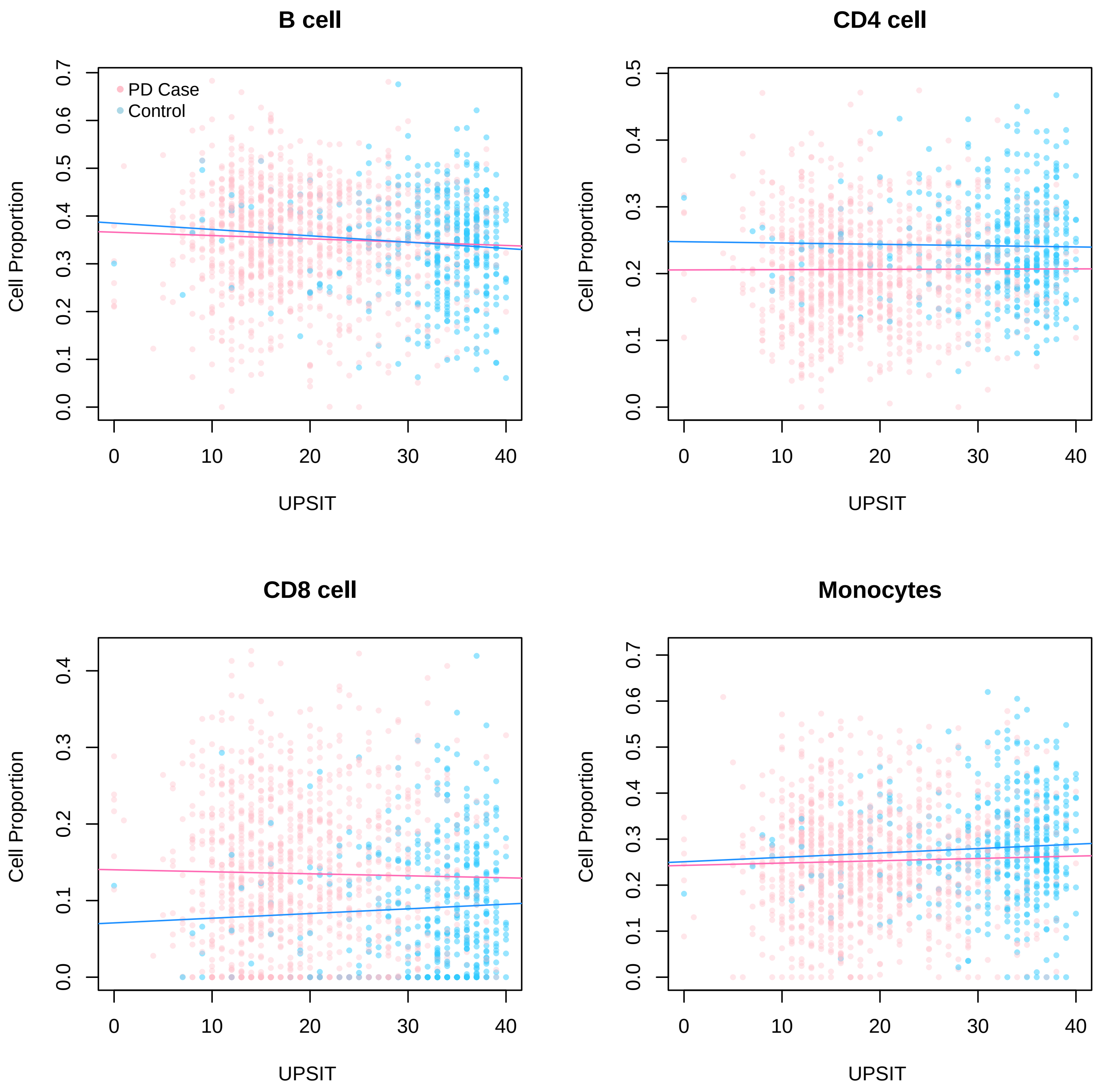

Figure S21: Relationship between total Smell Identification Test Score (UPSIT) and cell type proportion of B cells, CD4 cells, CD8 cells, and Monocytes, respectively. Different colors illustrate PD cases and healthy controls.

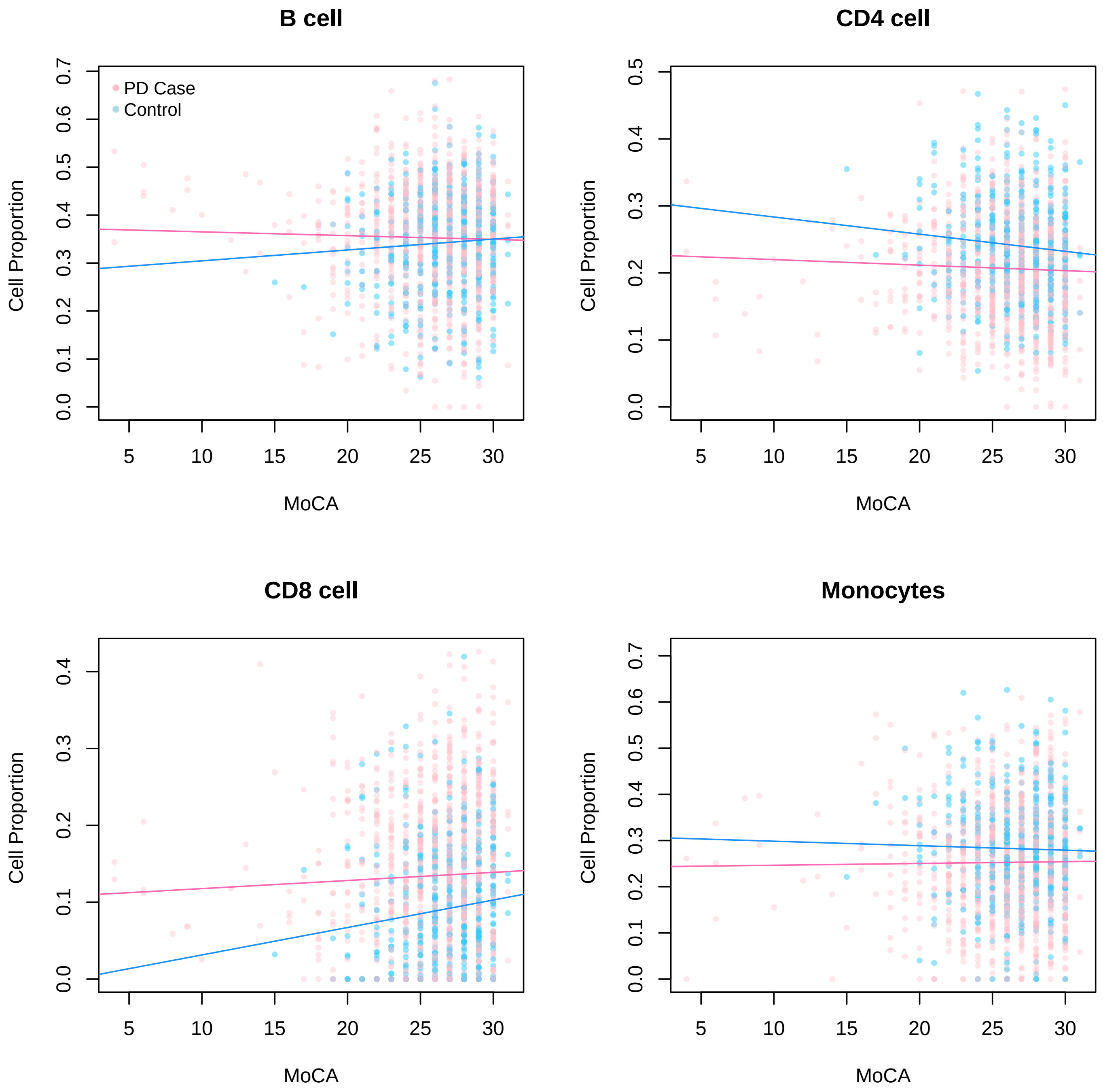

Figure S22: Relationship between total Montreal Cognitive Assessment Score (MoCA) and cell type proportion of B cells, CD4 cells, CD8 cells, and Monocytes, respectively. Different colors illustrate PD cases and healthy controls.

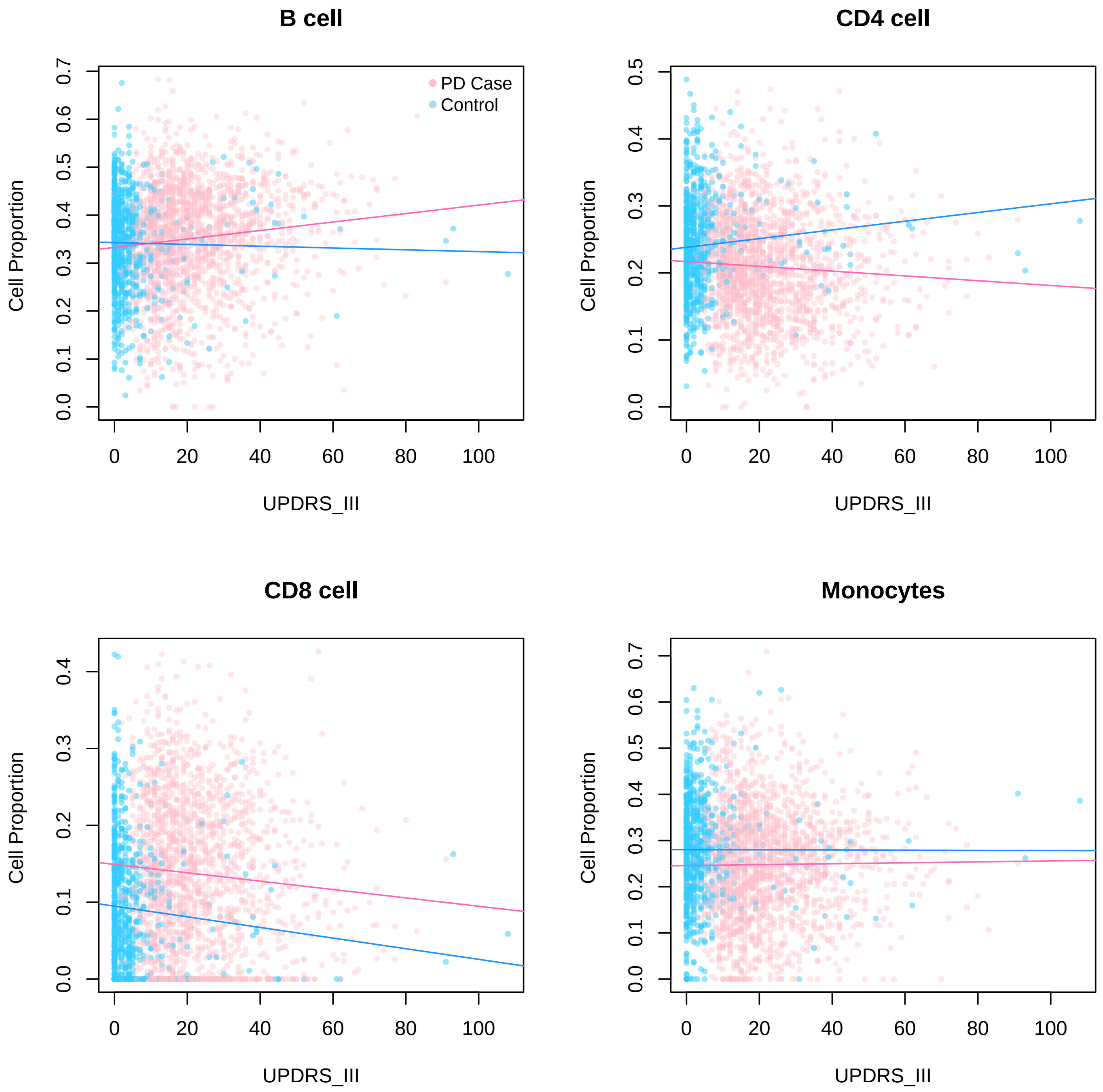

Figure S23: Relationship between total Unified Parkinson's Disease Rating Scale Part III (UPDRS III) and cell type proportion of B cells, CD4 cells, CD8 cells, and Monocytes, respectively. Different colors illustrate PD cases and healthy controls.

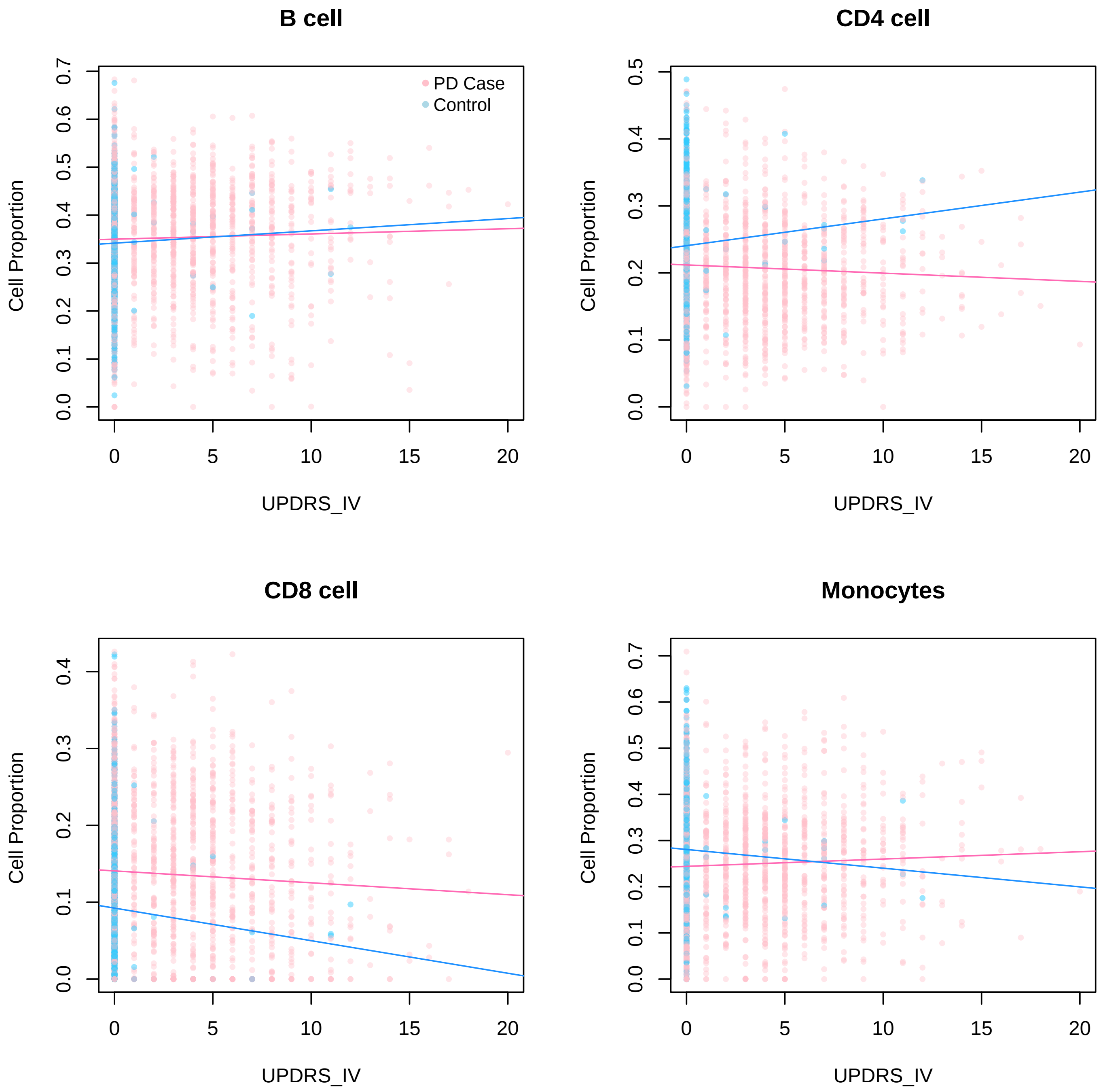

Figure S24: Relationship between total Unified Parkinson's Disease Rating Scale Part IV (UPDRS IV) and cell type proportion of B cells, CD4 cells, CD8 cells, and Monocytes, respectively. Different colors illustrate PD cases and healthy controls.

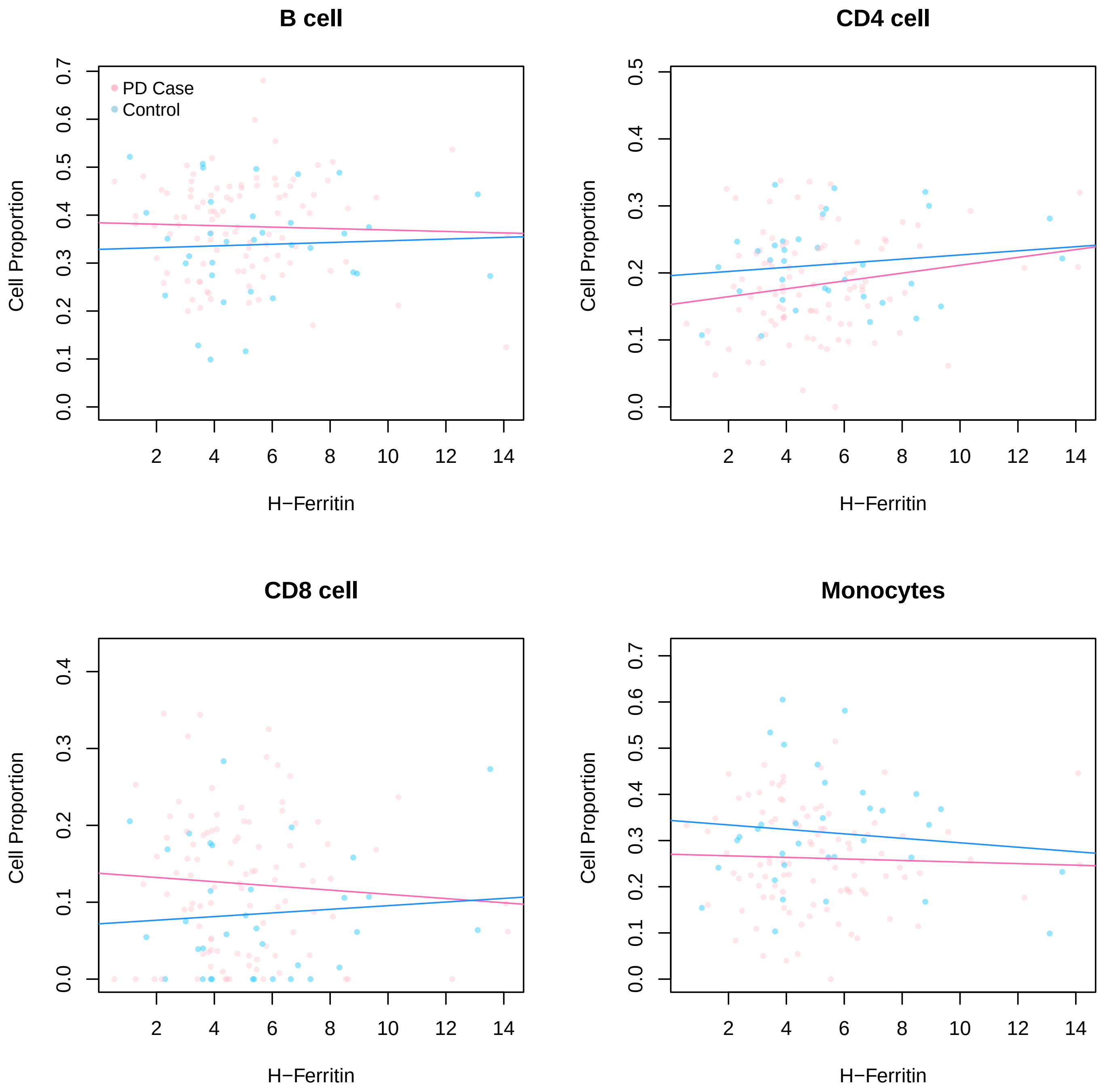

Figure S25: Relationship between Cerebrospinal fluid ferritin levels (H-Ferritin) and cell type proportion of B cells, CD4 cells, CD8 cells, and Monocytes, respectively. Different colors illustrate PD cases and healthy controls.

5.2 TEDDY: The Environmental Determinants of Diabetes in the Young.

This plots shows that the NK cell proportions, solved by CIBERSORT, among participants who developed IA at a young age compared to controls. There is no discernible difference in the proportion trends between cases and controls.

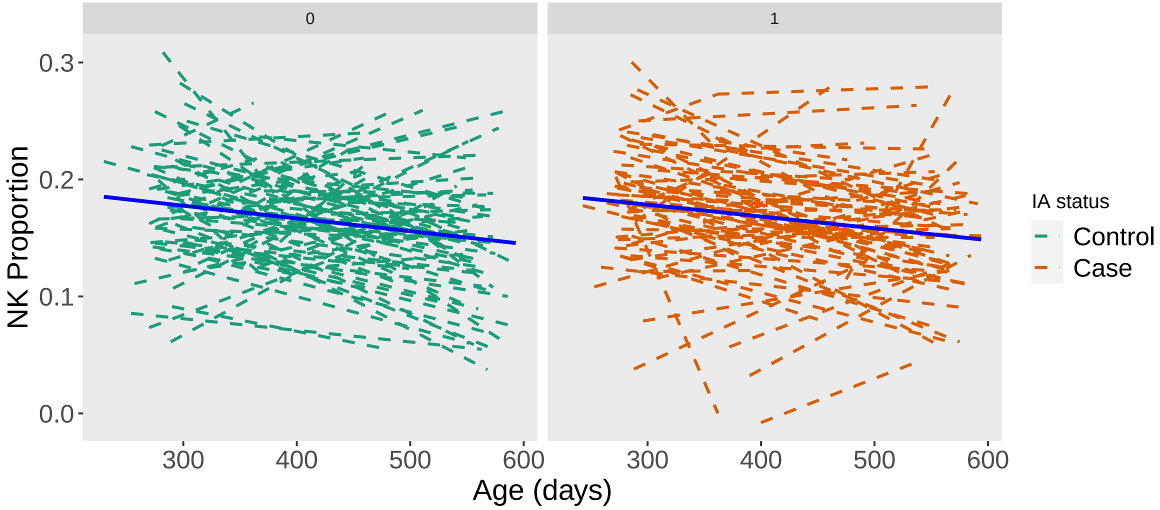

Figure S26: NK cell proportions along infant's age (in days) at sample collection, by case and control status. Average fitted lines (solid) overlay individual-specific lines (dashed).

6 Additional Benchmark

We have conducted additional benchmarking by comparing ***imply*** with both statistical modeling methods (CIBERSORTx (Newman et al. [2019]) and MuSiC (Wang et al. [2019])), and deep learning-based models (Scaden (Menden et al. [2020]) and TAPE (Chen et al. [2022])). It is not straightforward to benchmark ***imply***, MuSiC, Scaden, and TAPE because of different input requirements. MuSiC, Scaden, and TAPE require scRNA-sequencing data for their algorithms. Nevertheless, we proceeded with a comprehensive benchmark study, setting up slightly different simulations to assess ***imply***, Scaden, TAPE, and MuSiC (**Figure S27**), as well as ***imply*** and CIBERSORTx (**Figure S28**).

We employed a scRNA-seq dataset from PBMC (GSE149689) (Lee et al. [2020], Choi et al. [2022]) to simulate bulk data. We simulated two groups for comparison, each comprising 50 subjects. Six distinct cell types (B cell, CD4, CD8, Monocyte, NK, and others) were retained from this dataset for analysis, resulting in a total of 17,590 cells. We selected 3,000 genetic features based on coefficient of variation (CV) ranking, computed as the expression sum for each feature across all observations for each cell type. The simulation generated 100 personalized reference panels. Subsequent procedures closely followed the simulation method outlined in the main manuscript. Notably, Scaden and TAPE were executed on a Python 3.9.12 platform, utilizing an Intel Core i9 processor with 64.0 GB of RAM. For TAPE, we set the normalized parameters by specifying the datatype as 'TPM' and providing a gene length file. Scaden was implemented within the TAPE module using default settings. Input data, including both scRNA-seq and bulk sequencing data, were formatted according to the specific requirements of each algorithm. To assess the performance of these models, we conducted a comparative analysis by evaluating the estimated cell proportions against the true simulation values.

In **Figure S27 A**, a scatterplot depicts the estimated cell-type proportions against the true proportions, with ***imply*** results overlaid on top of the Scaden, TAPE, and MuSiC's estimates. **Figure S27 B** and **C** show the sum of absolute bias and correlation with the ground true simulated proportions, respectively, for ***imply***, Scaden, TAPE, and MuSiC. The red dashed lines at zero and one denote perfect agreements between the estimates and the truth. ***imply*** consistently outperforms other methods.

Figure S28 focuses on benchmarking ***imply*** and CIBERSORTx. **Figure S28 A** displays estimated cell-type proportions versus true proportions for a specific iteration, with ***imply*** estimated results overlaid on CIBERSORTx's estimates. **Figure S28 B** and **C** show the sum of absolute bias and correlation with the ground truth proportions for ***imply*** and CIBERSORTx. ***imply*** exhibits slightly superior and stable performance compared to CIBERSORTx.

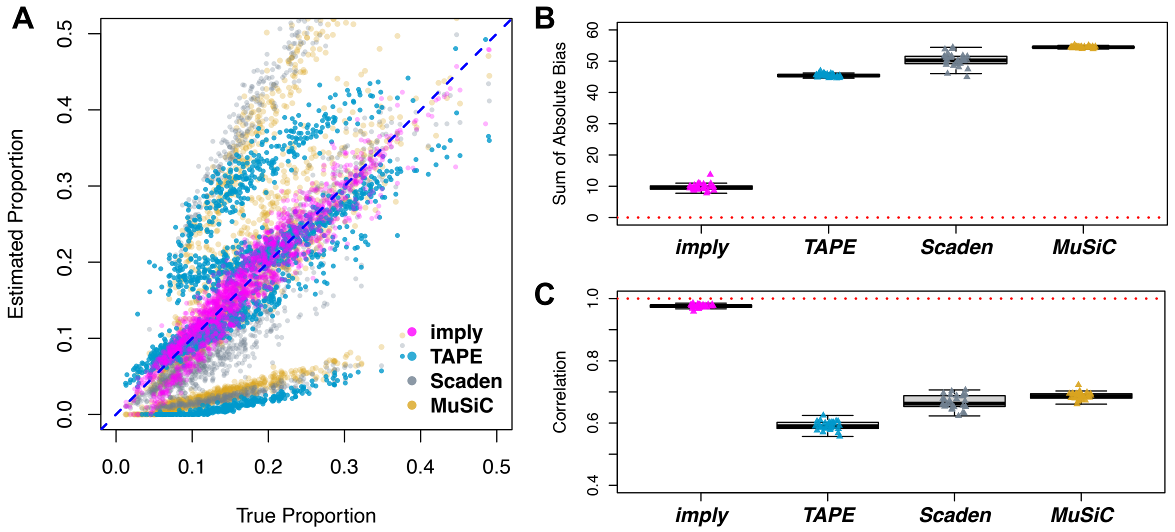

Figure S27: Benchmark ***imply*** against TAPE, Scaden, and MuSiC. The simulated bulk data is generated from a real single-cell PBMC data, with deconvolution for TAPE, Scaden, and MuSiC performed using the same single-cell dataset. (A) Superimposed scatterplot displaying the cell proportions estimated by ***imply*** overlaid on TAPE, Scaden, and MuSiC estimates. (B) Comparison of the sum of absolute bias with respect to the true proportion for ***imply***, TAPE, Scaden, and MuSiC. (C) Comparison of the estimated proportions' correlation with the ground truth for ***imply***, TAPE, Scaden, and MuSiC.

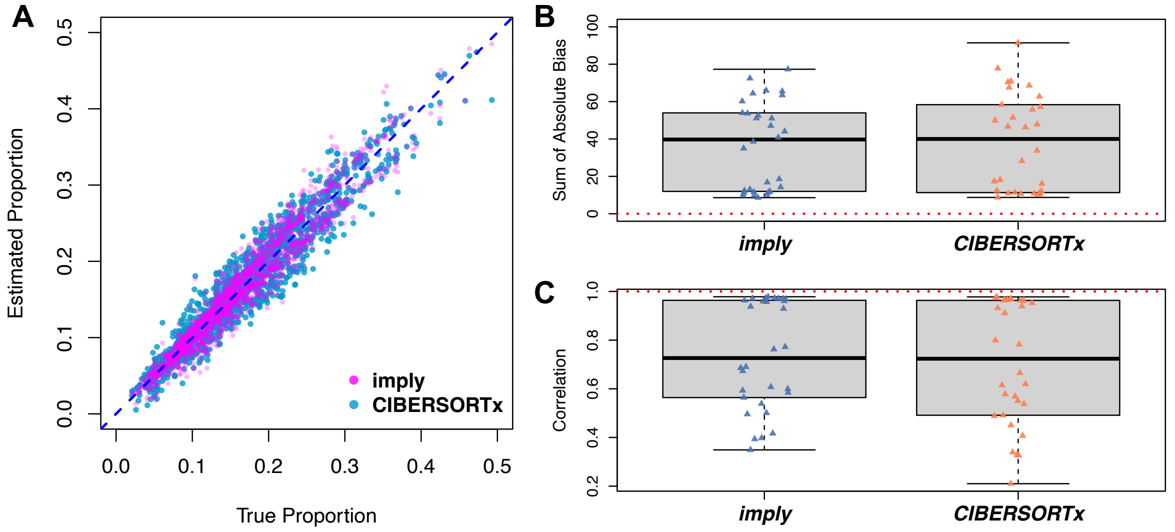

Figure S28: Benchmark ***imply*** against CIBERSORTx. The simulated bulk data is from the original simulation pipeline mentioned in the main manuscript. (A) Superimposed scatterplot shows the cell proportions estimated by ***imply*** overlaid on CIBERSORTx estimates. (B) Comparison of the sum of absolute bias with respect to the true proportion for ***imply*** and CIBERSORTx. (C) Comparison of the estimated proportions' correlation with the ground truth for ***imply*** and CIBERSORTx.

7 Statistical Test

Statistical tests were performed to see whether ***imply*** is significantly superior than other models. The tables in this section illustrate the pair-wise Wilcoxon signed-rank test p-values of ***imply*** and ***imply-s*** versus TCA-s, TCA-n, ISLET-s, and ISLET-n, respectively. These tests were conducted in a setting where the number of subjects per group is fixed at 200, and the number of marker genes equals 300.

Table S3: Pair-wise Wilcoxon signed-rank test p values for Absolute Bias Differences (ABD) between ***imply*/*imply-s*** and other methods under different effect sizes, where the Log-Fold Change (LFC) is set to 0-1.5.

LFC	Method	TCA-s	TCA-n	ISLET-s	ISLET-n
0	***imply***	0.935	1.863e-09	3.790e-06	4.265e-02
	imply-s	0.371	5.588e-09	4.971e-02	1.061e-05
0.75	***imply***	0.026	9.313e-09	0.000	0.919
	imply-s	0.067	9.313e-09	0.271	0.416
1	***imply***	3.663e-05	2.287e-06	0.002	0.008
	imply-s	1.765e-05	8.196e-07	0.002	0.0005
1.25	***imply***	0.001	1.542e-06	0.000	0.001
	imply-s	0.005	6.735e-06	0.005	0.005
1.5	***imply***	0.002	0.002	3.904e-05	0.009
	imply-s	0.009	0.003	1.886e-04	0.006

Table S4: Pair-wise Wilcoxon signed-rank test p values for relative Absolute Bias Differences ($rABD$) between ***imply*/*imply-s*** and other methods under different effect sizes, where the Log-Fold Change (LFC) is set to 0-1.5.

LFC	Method	TCA-s	TCA-n	ISLET-s	ISLET-n
0	***imply***	0.670	3.725e-09	7.994e-06	7.672e-02
	imply-s	0.404	3.725e-09	3.387e-01	7.994e-065
0.75	***imply***	0.001	9.313e-09	0.001	0.887
	imply-s	0.01	2.608e-08	0.490	0.516
1	***imply***	1.765e-05	9.395e-06	0.001	0.006
	imply-s	2.754e-05	7.965e-06	0.008	0.001
1.25	***imply***	0.004	2.459e-07	0.001	0.006
	imply-s	0.014	5.677e-06	0.010	0.036
1.5	***imply***	0.008	0.001	6.287e-05	0.010
	imply-s	0.064	0.005	2.188e-03	0.014

Table S5: Pair-wise Wilcoxon signed-rank test p values for Correlation Difference (CD) between ***imply***/***imply-s*** and other methods under different effect sizes, where the Log-Fold Change (LFC) is set to 0-1.5.

LFC	Method	TCA-s	TCA-n	ISLET-s	ISLET-n
0	***imply***	0.124	1.863e-09	9.220e-06	1.397e-05
	imply-s	0.824	3.725e-09	6.666e-02	2.608e-08
0.75	***imply***	1.419e-06	1.863e-09	0.000	0.984
	imply-s	8.705e-03	1.863e-09	0.871	0.903
1	***imply***	4.098e-07	3.725e-08	0.001	0.011
	imply-s	7.965e-06	2.235e-08	0.017	0.006
1.25	***imply***	0.000	1.490e-08	0.001	0.114
	imply-s	0.014	9.395e-06	0.099	0.339
1.5	***imply***	0.000	0.000	0.052	0.3280
	imply-s	0.047	0.003	0.428	0.700

Table S6: Pair-wise Wilcoxon signed-rank test p values for Euclidean distance-based CCC difference ($\Delta\rho_{\mathrm{C,E}}$) between ***imply***/***imply-s*** and other methods under different effect sizes, where the Log-Fold Change (LFC) is set to 0-1.5.

LFC	Method	TCA-s	TCA-n	ISLET-s	ISLET-n
0	***imply***	0.229	1.863e-09	2.349e-06	7.057e-05
	imply-s	0.655	3.725e-09	4.645e-01	6.147e-08
0.75	***imply***	2.349e-06	1.863e-09	7.911e-05	0.537
	imply-s	1.583e-03	1.863e-09	8.078e-01	0.630
1	***imply***	7.451e-08	1.490e-08	0.001	0.019
	imply-s	2.287e-06	7.451e-09	0.009	0.015
1.25	***imply***	3.330e-06	7.451e-09	0.001	0.015
	imply-s	7.162e-04	1.885e-06	0.048	0.099
1.5	***imply***	0.000	7.994e-06	0.001	0.369
	imply-s	0.005	3.450e-04	0.035	0.536

8 Impact of Absent Cell Type on *imply*'s Performance

We additionally investigate the model's performance when a cell type is missing from the bulk during deconvolution. We followed the existing approach to simulate bulk data from six cell types. However, we masked the cell type 6 from the signature matrix. The figure below shows the baseline scenario mentioned in the main text: two groups with 100 subjects per group, each having three replicates. **Figure S29 A** displays the scatterplot of the estimated cell-type proportions versus the true proportions. ***imply*** estimated results are overlaid on top of the CIBERSORT estimated result. ***imply*** yields more accurate deconvolution results because its estimates aggregate closer to the diagonal line. **Figure S29 B-E** show bias reductions, which are quantitatively assessed by metrics introduced in the main manuscript: Absolute Bias Difference (*ABD*), relative ABD (*rABD*), Correlation Differences (*CD*), and Euclidean-distance based CCC differences ($\Delta\rho_{\text{C,E}}$). Each point in **Figure S29 B-E** represents one iteration, and the red dashed lines at zero serve as a reference for not using personalized reference panels. For *ABD* and *rABD*, lower values indicate a greater increase in deconvolution accuracy, but for *CD* and $\Delta\rho_{\text{C,E}}$, higher values indicate improved concordance with the true values. Notably, ***imply*** consistently provides the most substantial reduction in deconvolution bias and the highest concordance with the truth, even when a cell type is missing.

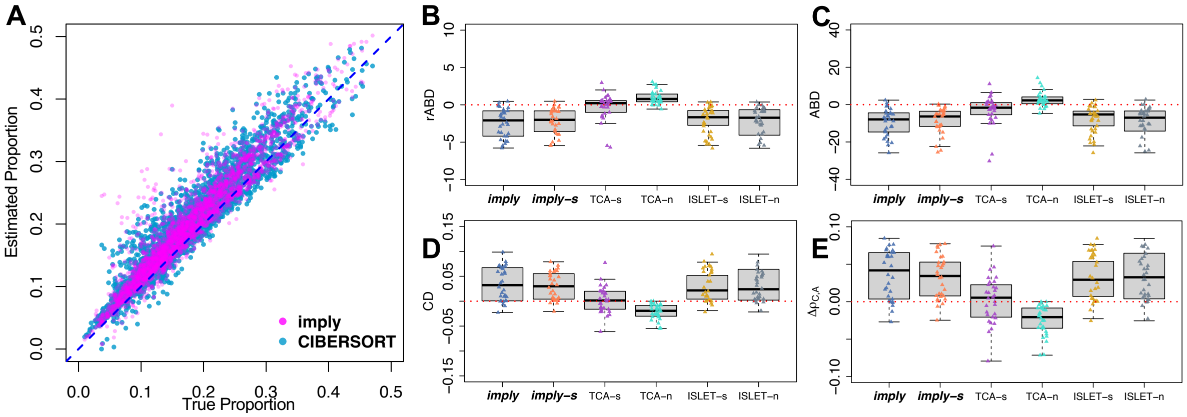

Figure S29: ***imply*** can maintain the cell type deconvolution accuracy when a cell type is missing from the bulk. The simulated bulk data is generated from six cell types, while the deconvolution is performed on only five cell types. (A) Superimposed scatterplot of the ***imply***-estimated cell type proportions over the CIBERSORT estimates. Deconvolutions for both methods are based on five cell types. (B)-(E) Boxplots displaying evaluation metrics (*ABD*, *rABD*, *CD*, and $\Delta\rho_{\text{C,E}}$) when the deconvolution is performed with one cell type masked. Each point represents one simulation iteration, and the red dashed line represents no improvement in deconvolution. For (B) and (C), lower values indicate better deconvolution accuracy. For (E) and (F), higher values demonstrate better performance.

9 Iterate *imply* to Further Reduce Bias

We could iterate ***imply*** by using the results in stage 3 as the initial deconvoluted matrix in stage 1, and then conduct an iteration to repeat stages 2 and 3. We conducted a small scale simulation to investigate the improvements of iterations. The figure below shows the simulation results for ***imply*** under the baseline scenarios mentioned in the main manuscript. We use the ***imply***-solved cell proportion as the input for stage 1 and iteratively apply ***imply*** five times. *ABD* is adapted to quantitatively evaluate estimation improvements, where lower *ABD* values indicate a greater increase in deconvolution accuracy.

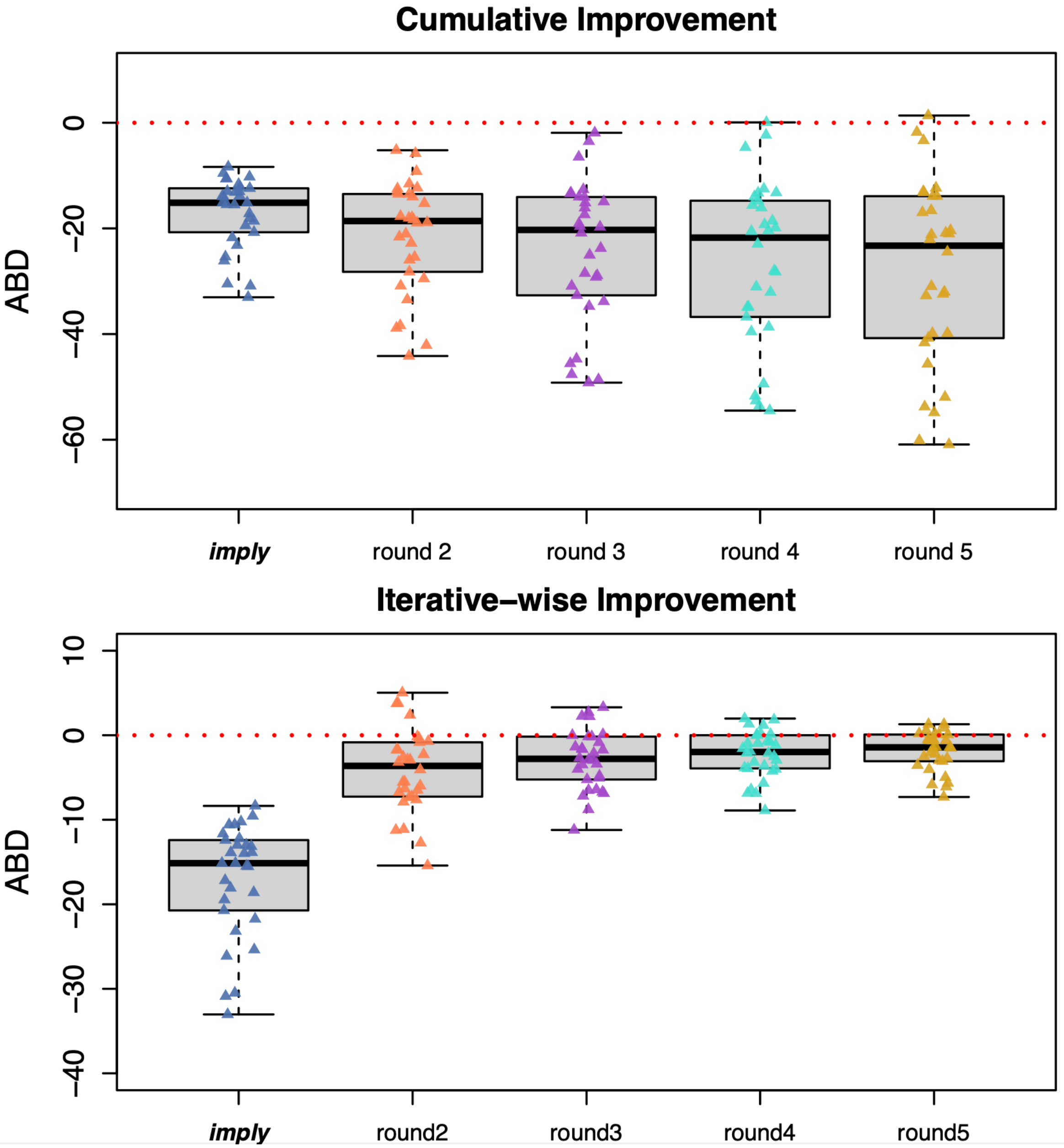

Figure S30: Deconvolution bias reduction with iterative ***imply*** implementation: ***imply*** is iterated up to five times, and bias reductions are evaluated by *ABD* values. The upper panel shows the cumulative improvement, and the lower panel displays the iterative-wise improvement. A lower *ABD* values indicate enhanced deconvolution accuracy, with the red dashed line denoting no improvement.

10 Impact of Cellular Abundance on *imply*'s Performance

To investigate the impact of cellular abundance, especially for cell types with minimal proportions, we adjusted the Dirichlet distribution by setting one of the six cell types to approximately 5%. Simulations was applied within the framework of the same baseline scenario detailed in the main manuscript. Figures below illustrate the impact of cellular abundance on the performance of ***imply*** and ***imply-s***.

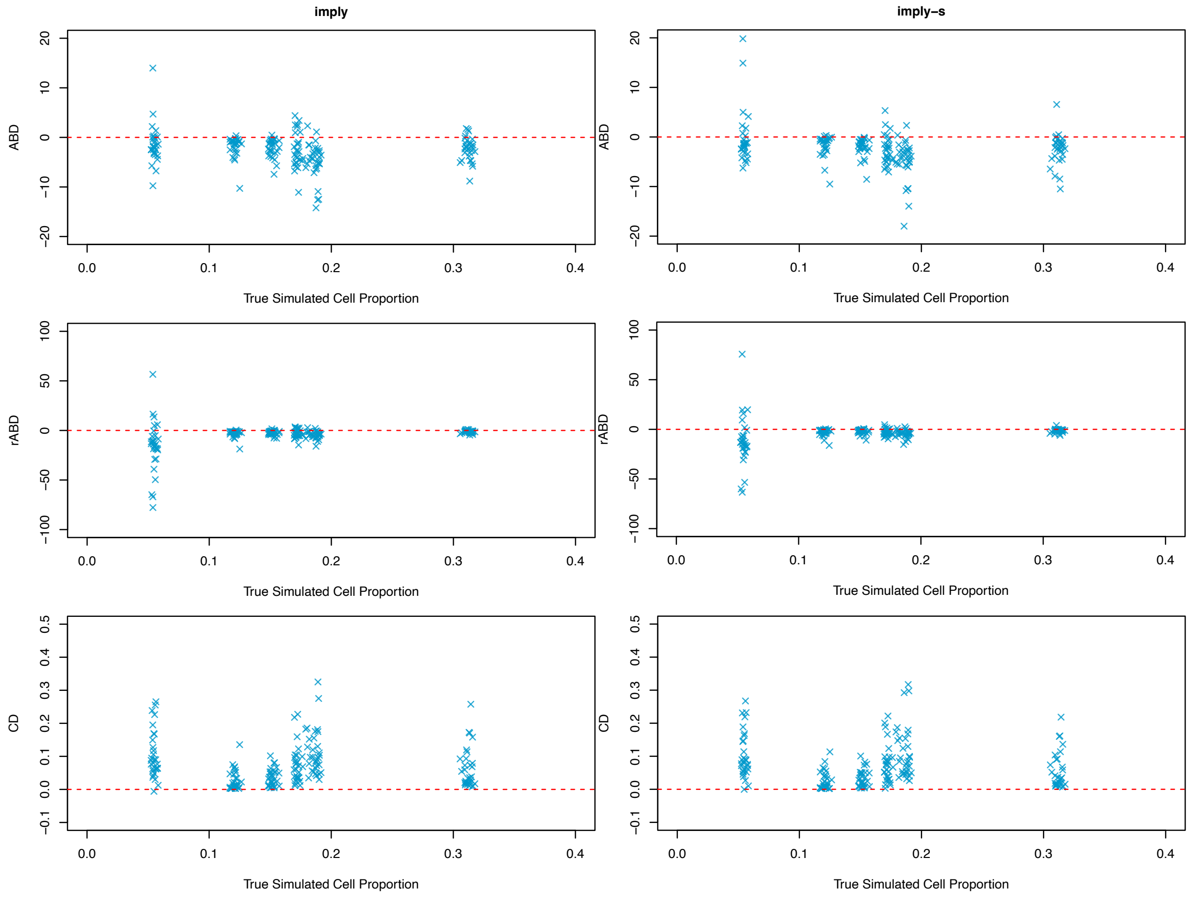

Figure S31: Deconvolution improvements examined across levels of cellular abundance, ranging from 5% up to 30% of true simulated cell type proportions, depicted on the horizontal axis. Both ***imply*** (left), and ***imply-s*** (right) are evaluated using *ABD* (top), *rABD* (middle), and *CD* (bottom) metrics.

11 Impact of Subject Sample Size on *imply*'s Performance

We investigate if the number of samples per subject would also influence the deconvolution accuracy and how many samples per subject there should be at least. The evaluation metrics, *ABD*, *rABD*, and *CD*, are employed. We initially generated 10 longitudinal samples for each subject in the data simulation step. Subsequently, during the model implementation step, we selected 2, 4, 6, 8, and 10 samples per subject (as depicted on the horizontal axis) from the simulated pool, with larger sample sizes inclusive of the previous samples.

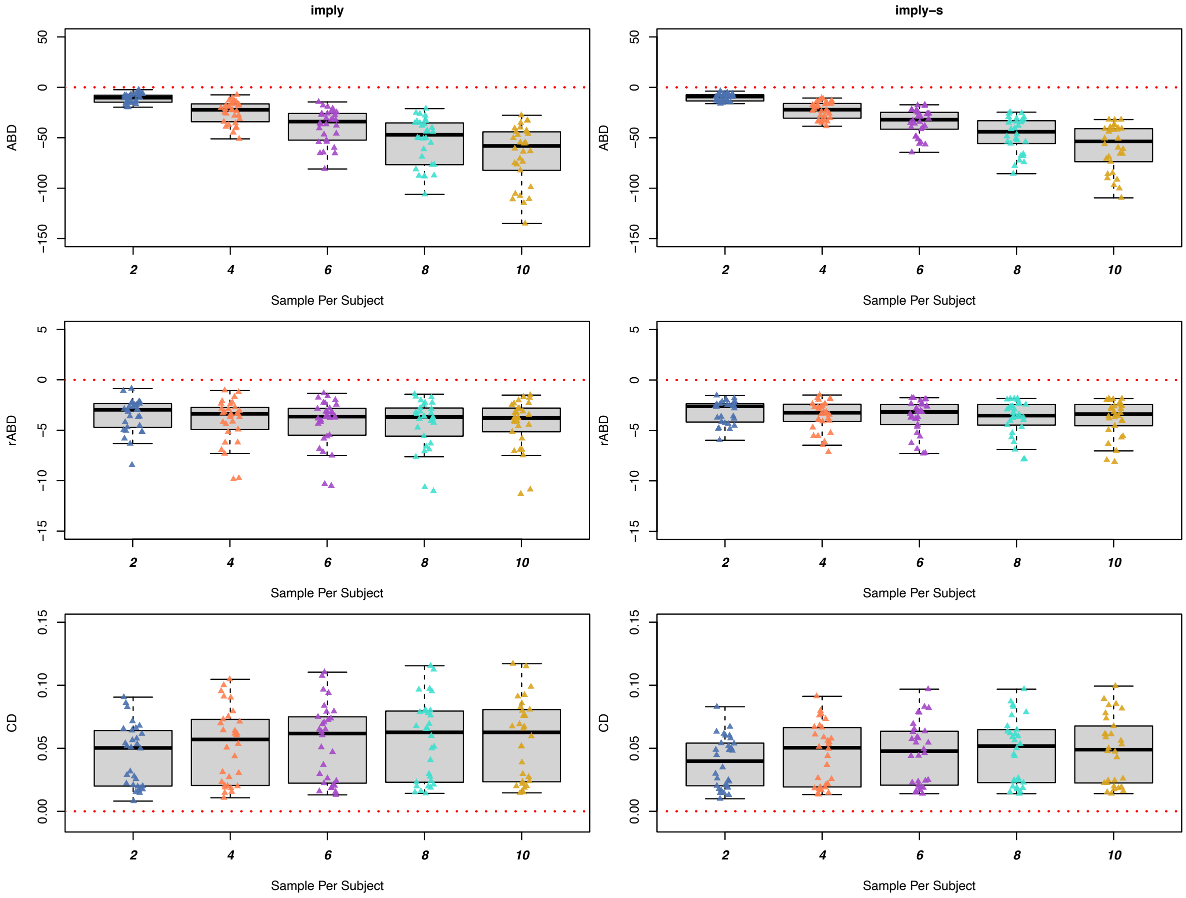

Figure S32: Boxplots depicting evaluation metrics, including *ABD* (top), *rABD* (middle), and *CD* (bottom), for ***imply*** (left) and ***imply-s*** (right). Each point represents one iteration, and the red dashed line indicates no improvement in deconvolution. The horizontal axis shows the number of simulated samples for each subject.

12 Benchmark Algorithms on PBDP and TEDDY Data

We incorporated the findings obtained from TCA-s, ISLET-n, and ISLET-s into the datasets of the Parkinson's Disease Biomarker Program (PDBP) and The Environmental Determinants of Diabetes in the Young (TEDDY). For TEDDY dataset, given the potential sex disparities in gene expression and NK cell abundance, we used both disease status (islet autoimmunity) and sex as cell-type-specific covariates in the design matrix to directly adjust for these additional covariates.

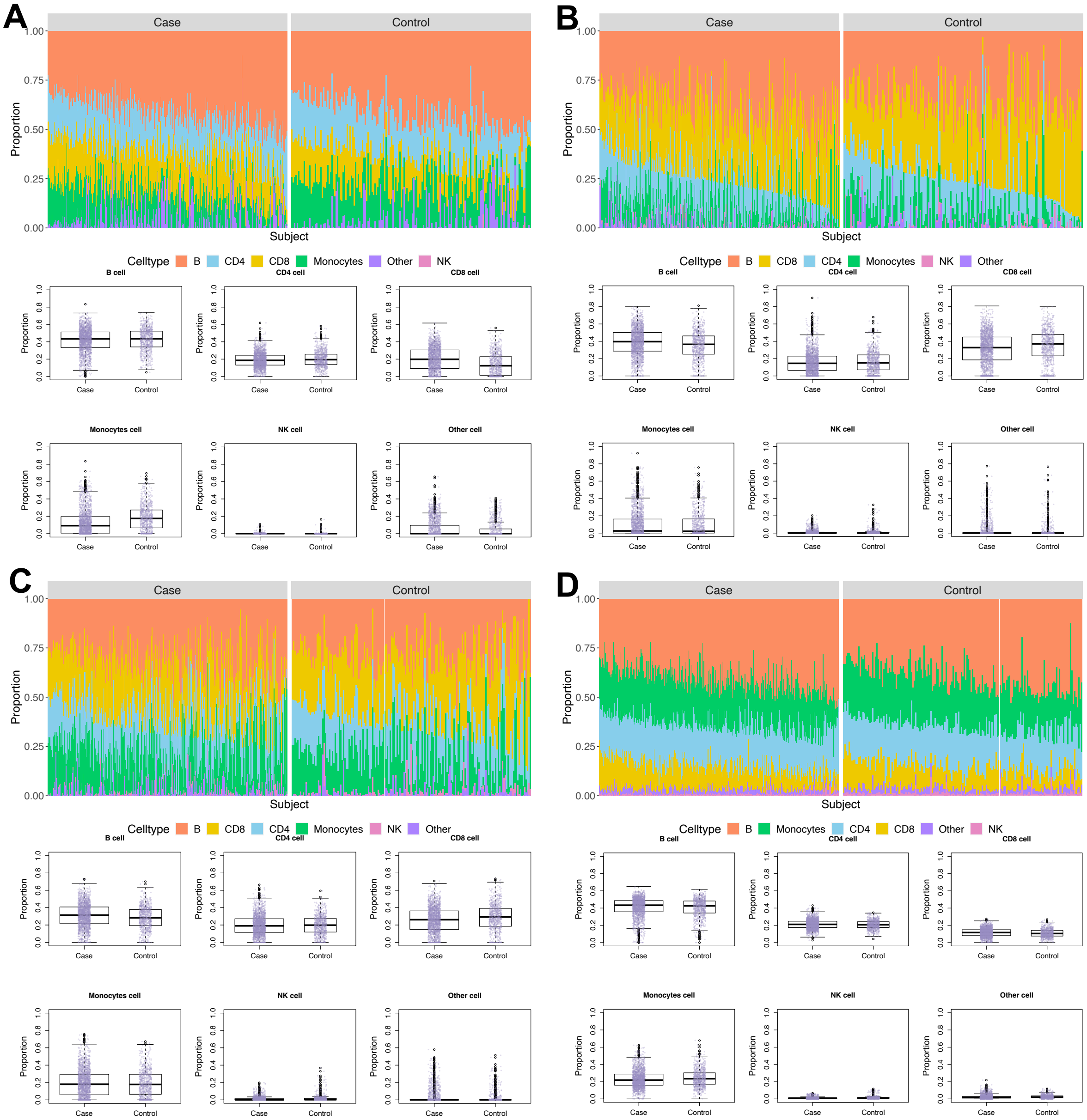

Figure S33: Phenotype-associated cell type disparities observed in the Parkinson's Disease Biomarker Program (PDBP) consortia. Panels (A)-(D) respectively show cell proportion deconvolution results implemented by ***imply***, ISLET-n, ISLET-s, and TCA-s. Within each indexed panel, the top figures show the concatenated mean proportions across all visit times for six cell types. The bottom figures display the direct proportion comparisons between cases and controls for each specific cell type.

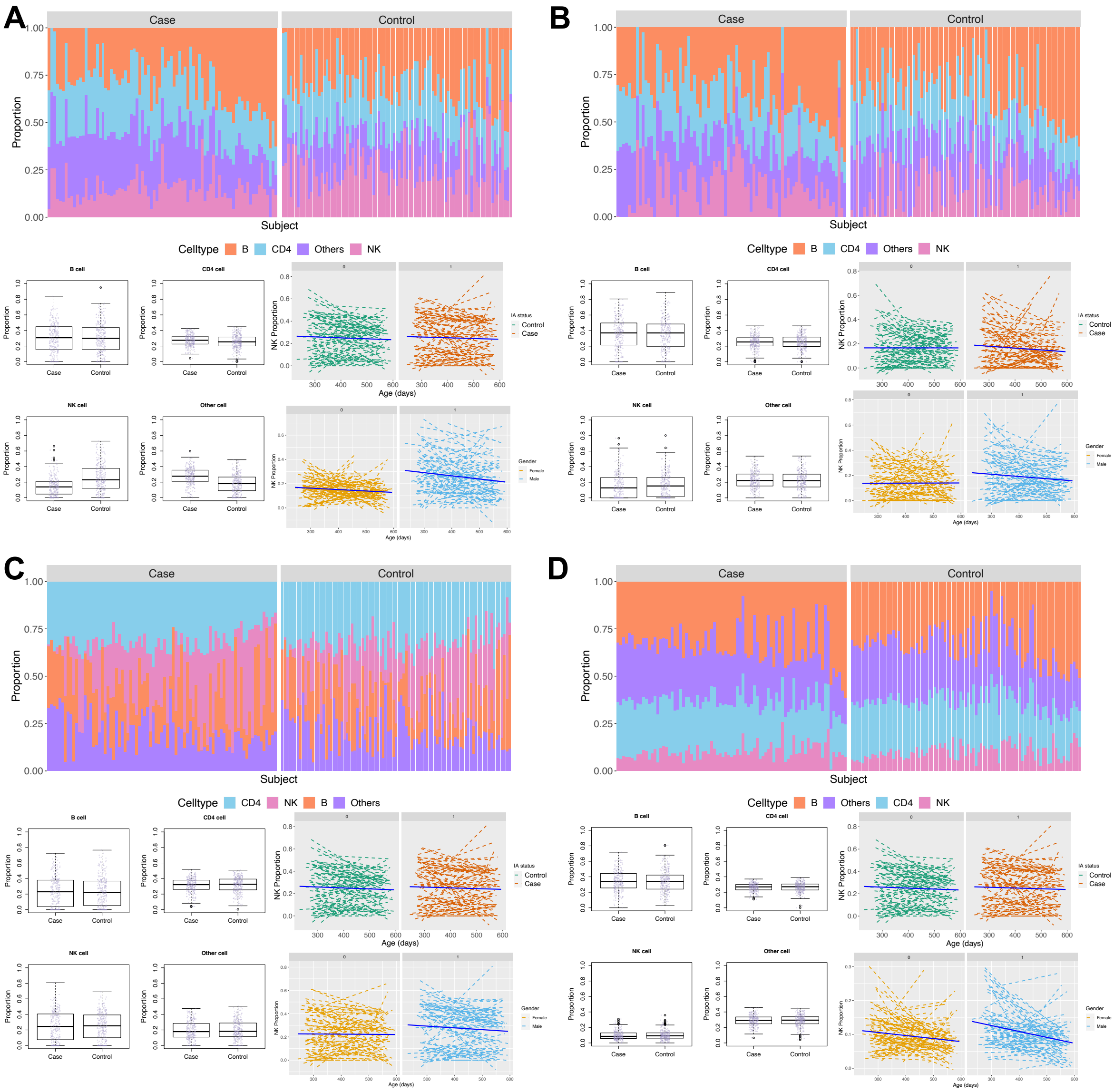

Figure S34: Phenotype-associated cell type disparities observed in The Environmental Determinants of Diabetes in the Young (TEDDY) consortia. Panels (A)-(D) respectively show cell proportion deconvolution results implemented by ***imply***, ISLET-n, ISLET-s, and TCA-s. Within each indexed panel, the top figures show the concatenated mean proportions across all visit times for six cell types. The bottom left figures provide direct proportion comparisons between case and control for each cell type. The bottom right figures within each panel show the NK cell proportions along the infant's age (in days) at sample collection, comparing cases versus controls and female versus male subjects. The average fitted lines (solid) overlay individual-specific lines (dashed).

13 Benchmark Computational Time and Resources

The figure below represents the baseline scenarios. The run time, measured in seconds, is shown on the vertical axis, while the model names are denoted on the horizontal axis. The simulations were executed on a MacBook Pro laptop equipped with 32 GB of memory and an M1 Max Apple Chip. The boxplots below illustrate the computational times for each method across iterations.

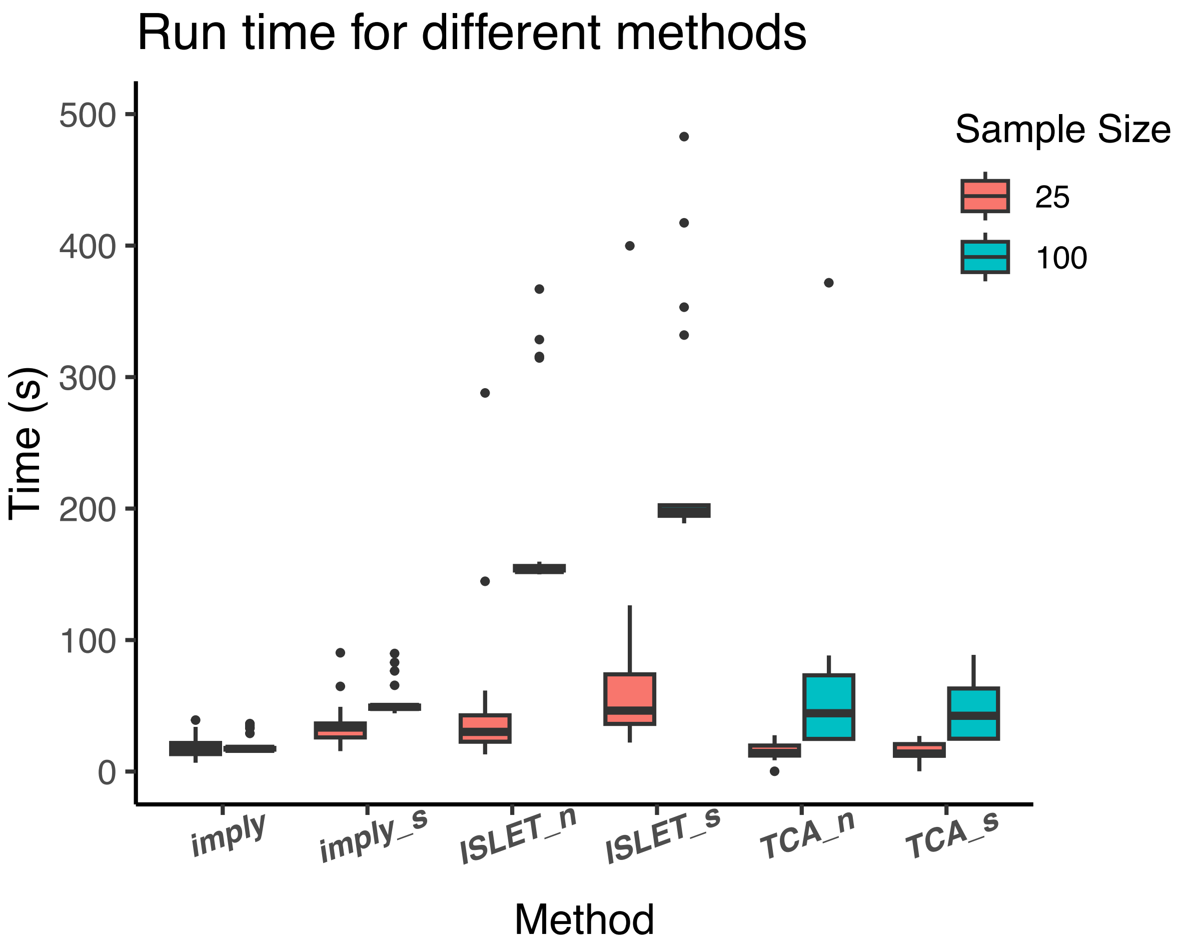

Figure S35: Computational times (seconds) for each model under a different number of subjects per group.

References

J. Aitchison, C. Barceló-Vidal, J. A. Martín-Fernández, and V. Pawlowsky-Glahn. Logratio analysis and compositional distance. *Mathematical geology*, 32:271–275, 2000.

M. Carlson. *org.Hs.eg.db: Genome wide annotation for Human*, 2021. R package version 3.14.0.

Y. Chen, Y. Wang, Y. Chen, Y. Cheng, Y. Wei, Y. Li, J. Wang, Y. Wei, T.-F. Chan, and Y. Li. Deep autoencoder for interpretable tissue-adaptive deconvolution and cell-type-specific gene analysis. *Nature Communications*, 13(1):6735, 2022.

B. Choi, C. K. Kang, S. Park, D. Lee, A. J. Lee, Y. Ko, S.-J. Kang, K. Kang, S. Kim, Y. Koh, et al. Single-cell transcriptome analyses reveal distinct gene expression signatures of severe covid-19 in the presence of clonal hematopoiesis. *Experimental & molecular medicine*, 54(10):1756–1765, 2022.

Y. Cui, L. Peng, Y. Hu, and H. J. Lai. Assessing the reproducibility of microbiome measurements based on concordance correlation coefficients. *Journal of the Royal Statistical Society Series C: Applied Statistics*, 70(4):1027–1048, 2021.

D. S. DeLuca, J. Z. Levin, A. Sivachenko, T. Fennell, M.-D. Nazaire, C. Williams, M. Reich, W. Winckler, and G. Getz. Rna-seqc: Rna-seq metrics for quality control and process optimization. *Bioinformatics*, 28(11):1530–1532, 2012.

A. Dobin, C. A. Davis, F. Schlesinger, J. Drenkow, C. Zaleski, S. Jha, P. Batut, M. Chaisson, and T. R. Gingeras. Star: ultrafast universal rna-seq aligner. *Bioinformatics*, 29(1):15–21, 2013.

J. Hwang, D. Choi, S. Han, S. Y. Jung, J. Choi, and J. Hong. Potential toxicity of polystyrene microplastic particles. *Scientific reports*, 10(1):7391, 2020.

A. E. Jaffe, R. Tao, A. L. Norris, M. Kealhofer, A. Nellore, J. H. Shin, D. Kim, Y. Jia, T. M. Hyde, J. E. Kleinman, et al. qsva framework for rna quality correction in differential expression analysis. *Proceedings of the National Academy of Sciences*, 114(27):7130–7135, 2017.

J. S. Lee, S. Park, H. W. Jeong, J. Y. Ahn, S. J. Choi, H. Lee, B. Choi, S. K. Nam, M. Sa, J.-S. Kwon, et al. Immunophenotyping of covid-19 and influenza highlights the role of type i interferons in development of severe covid-19. *Science immunology*, 5(49):eabd1554, 2020.

H. Li, B. Handsaker, A. Wysoker, T. Fennell, J. Ruan, N. Homer, G. Marth, G. Abecasis, R. Durbin, and . G. P. D. P. Subgroup. The sequence alignment/map format and samtools. *bioinformatics*, 25 (16):2078–2079, 2009.

Y. Liao, G. K. Smyth, and W. Shi. featurecounts: an efficient general purpose program for assigning sequence reads to genomic features. *Bioinformatics*, 30(7):923–930, 2014.

P. S. Linsley, C. Speake, E. Whalen, and D. Chaussabel. Copy number loss of the interferon gene cluster in melanomas is linked to reduced t cell infiltrate and poor patient prognosis. *PloS one*, 9 (10):e109760, 2014.

K. Menden, M. Marouf, S. Oller, A. Dalmia, D. S. Magruder, K. Kloiber, P. Heutink, and S. Bonn. Deep learning–based cell composition analysis from tissue expression profiles. *Science advances*, 6 (30):eaba2619, 2020.

A. M. Newman, C. L. Liu, M. R. Green, A. J. Gentles, W. Feng, Y. Xu, C. D. Hoang, M. Diehn, and A. A. Alizadeh. Robust enumeration of cell subsets from tissue expression profiles. *Nature methods*, 12(5):453–457, 2015.

A. M. Newman, C. B. Steen, C. L. Liu, A. J. Gentles, A. A. Chaudhuri, F. Scherer, M. S. Khodadoust, M. S. Esfahani, B. A. Luca, D. Steiner, et al. Determining cell type abundance and expression from bulk tissues with digital cytometry. *Nature biotechnology*, 37(7):773–782, 2019.

A. Robitzsch. *sirt: Supplementary Item Response Theory Models*, 2022. URL `https://CRAN.R-project.org/package=sirt`. R package version 3.13-1.

B. Schölkopf, A. J. Smola, R. C. Williamson, and P. L. Bartlett. New support vector algorithms. *Neural computation*, 12(5):1207–1245, 2000.

X. Wang, J. Park, K. Susztak, N. R. Zhang, and M. Li. Bulk tissue cell type deconvolution with multi-subject single-cell expression reference. *Nature communications*, 10(1):380, 2019.

H. Wu, C. Wang, and Z. Wu. Proper: comprehensive power evaluation for differential expression using rna-seq. *Bioinformatics*, 31(2):233–241, 2015.
